# Metabolomic profiles predict individual multidisease outcomes

**DOI:** 10.1038/s41591-022-01980-3

**Published:** 2022-09-22

**Authors:** Thore Buergel, Jakob Steinfeldt, Greg Ruyoga, Maik Pietzner, Daniele Bizzarri, Dina Vojinovic, Julius Upmeier zu Belzen, Lukas Loock, Paul Kittner, Lara Christmann, Noah Hollmann, Henrik Strangalies, Jana M. Braunger, Benjamin Wild, Scott T. Chiesa, Joachim Spranger, Fabian Klostermann, Erik B. van den Akker, Stella Trompet, Simon P. Mooijaart, Naveed Sattar, J. Wouter Jukema, Birgit Lavrijssen, Maryam Kavousi, Mohsen Ghanbari, Mohammad A. Ikram, Eline Slagboom, Mika Kivimaki, Claudia Langenberg, John Deanfield, Roland Eils, Ulf Landmesser

**Affiliations:** 1 Center for Digital Health, Berlin Institute of Health at Charité – Universitätsmedizin Berlin, Berlin, Germany; 2 Department of Cardiology, Campus Benjamin Franklin, Charité – Universitätsmedizin Berlin and Berlin Institute of Health, Berlin, Germany; 3 Computational Medicine, Berlin Institute of Health at Charité – Universitätsmedizin Berlin, Berlin, Germany; 4 MRC Epidemiology Unit, Institute of Metabolic Science, University of Cambridge, Cambridge, UK; 5 Molecular Epidemiology, LUMC, Leiden, the Netherlands; 6 Leiden Computational Biology Center, LUMC, Leiden, The Netherlands; 7 Department of Epidemiology, Erasmus MC University Medical Center, Rotterdam, the Netherlands; 8 Molecular Epidemiology, Department of Biomedical Data Sciences, Leiden University Medical Center, Leiden, the Netherlands; 9 Institute of Cardiovascular Sciences, University College London, London, UK; 10 Department of Endocrinology & Metabolism, Charité – Universitätsmedizin Berlin and Berlin Institute of Health, Berlin, Germany; 11 Center for Cardiovascular Research, Charité – Universitätsmedizin Berlin and Berlin Institute of Health, Berlin, Germany; 12 Department of Neurology, Humboldt-Universität zu Berlin and Berlin Institute of Health, Charité-Universitätsmedizin Berlin, Berlin, Germany; 13 School of Mind and Brain, Humboldt-Universität zu Berlin, Berlin, Germany; 14 Delft Bioinformatics Lab, TU Delft, Delft, the Netherlands; 15 Department of Internal Medicine, Division of Gerontology and Geriatrics, Leiden University Medical Center, Leiden, the Netherlands; 16 Department of Cardiology, Leiden University Medical Center, Leiden, the Netherlands; 17 Institute of Cardiovascular and Medical Sciences, Cardiovascular Research Centre, University of Glasgow, Glasgow, UK; 18 Netherlands Heart Institute, Utrecht, the Netherlands; 19 Department of Surgery, Erasmus MC University Medical Center, Rotterdam, the Netherlands; 20 Max Planck Institute for the Biology of Ageing, Cologne, Germany; 21 Department of Epidemiology and Public Health, University College London, London, UK; 22 Clinicum, Faculty of Medicine, University of Helsinki, Helsinki, Finland; 23 Health Data Science Unit, Heidelberg University Hospital and BioQuant, Heidelberg, Germany

**Keywords:** Metabolomics, Predictive markers, Machine learning, Epidemiology

## Abstract

Risk stratification is critical for the early identification of high-risk individuals and disease prevention. Here we explored the potential of nuclear magnetic resonance (NMR) spectroscopy-derived metabolomic profiles to inform on multidisease risk beyond conventional clinical predictors for the onset of 24 common conditions, including metabolic, vascular, respiratory, musculoskeletal and neurological diseases and cancers. Specifically, we trained a neural network to learn disease-specific metabolomic states from 168 circulating metabolic markers measured in 117,981 participants with ~1.4 million person-years of follow-up from the UK Biobank and validated the model in four independent cohorts. We found metabolomic states to be associated with incident event rates in all the investigated conditions, except breast cancer. For 10-year outcome prediction for 15 endpoints, with and without established metabolic contribution, a combination of age and sex and the metabolomic state equaled or outperformed established predictors. Moreover, metabolomic state added predictive information over comprehensive clinical variables for eight common diseases, including type 2 diabetes, dementia and heart failure. Decision curve analyses showed that predictive improvements translated into clinical utility for a wide range of potential decision thresholds. Taken together, our study demonstrates both the potential and limitations of NMR-derived metabolomic profiles as a multidisease assay to inform on the risk of many common diseases simultaneously.

## Abstract (web summary)

In a study involving more than 100,000 individuals in the UK Biobank, a neural network model trained on metabolomic data can predict disease risk for over 20 conditions and adds predictive information over clinical variables for eight common diseases.

## Main

Risk stratification is central to disease prevention^1,2^. Over the past decade, increasingly complex information on an individual’s phenotype has become available beyond conventional demographic and laboratory information. While blood metabolites such as cholesterols are established clinical predictors for cardiovascular disease risk^3^, many more have been linked to common disease phenotypes^4–8^. In recent years, studies have moved beyond associations of individual markers by linking metabolomic profiles to aging^9^, disease onset^10^ and mortality^11^, appreciating the human blood metabolome as a direct reflection of the physiological state.

Proton nuclear magnetic resonance (1H-NMR) spectroscopy enables a standardized assessment of a multitude of small circulating molecules in the blood simultaneously. NMR differs from other techniques in metabolomics, such as mass spectrometry, by its virtual absence of batch effects, minimal requirements of expensive reagents and high throughput at comparatively low cost^12^. In the current assay >150 original markers are quantified, including amino and fatty acids and metabolites related to carbohydrate metabolism and fluid balance, partly overlapping with conventional clinical predictors including glucose, albumin and creatinine^13–15^. Further, the assay has a high resolution of lipoprotein particles, measuring their components, sizes and concentrations^13,14^. This high-throughput NMR metabolomics platform has been explored in multiple studies investigating all-cause mortality^11,16^, cardiovascular disease^13,17^, type 2 diabetes (T2D)^18,19^, Alzheimer’s disease^8^ and COVID-19 (ref. ^20^). Importantly, recent work has indicated a broad metabolic basis across diseases, suggesting a shared etiology^21^. This systemic information contained in metabolomic profiles has been insufficiently considered in the risk prediction of common diseases.

**Fig. 1:**
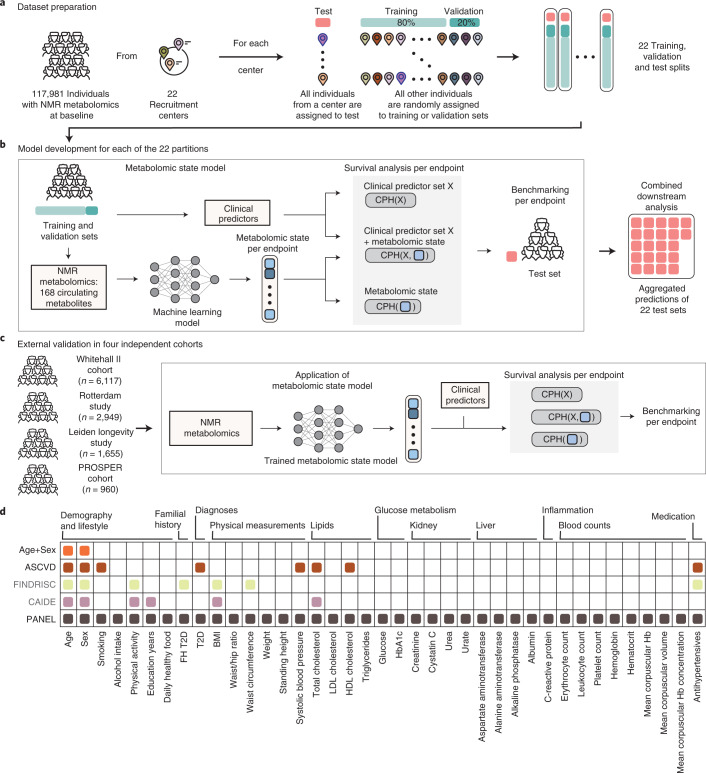
Study overview. **a**, To learn metabolomic states from circulating blood metabolites, the eligible UK Biobank population (with NMR blood metabolomics and valid consent) was split into training, validation and test sets with 22-fold nested cross-validation based on the assigned UK Biobank assessment center. **b**, For each of the 22 partitions, the metabolomic state model was trained on the 168 metabolomic markers to predict metabolomic risk against 24 common disease endpoints. Subsequently, for each endpoint, CPH models were developed on the metabolomic state in combination with sets of commonly available clinical predictors to model disease risk. Predictions of the CPH model on the test set were aggregated for downstream analysis. **c**, The metabolomic state model was externally validated in four independent cohorts—the Whitehall II cohort and three from the BBMRI-NL consortium: the Rotterdam Study, the Leiden Longevity Study and the PROSPER cohort. **d**, In this study we consider clinical predictors from scores commonly applied in primary prevention. We additionally integrate variables into a comprehensive predictor set (PANEL) to investigate overlapping information with the metabolomic state. FH, family history.

Here we exploited the potential of NMR-based blood profiling as a single-domain assay to simultaneously predict multidisease onset. We developed, trained and validated a deep residual multitask neural network to simultaneously learn disease-specific metabolomic states for 24 conditions, including common metabolic, vascular, respiratory, musculoskeletal and neurological disorders and cancers (Fig. 1). The scalar metabolomic states, contained in a 24-dimensional vector, were derived from 168 circulating metabolomic markers measured in ~120.000 individuals in the UK Biobank population cohort^22^. We extensively investigated the learned metabolomic states by integrating them in Cox proportional hazard (CPH) models^23^, modeling the risk for individual endpoints and demonstrating that information gained through NMR metabolomic profiling is additive to known clinical predictors. Moreover, we externally validated the metabolomic states in four independent cohorts, the Whitehall II cohort^24^, the Rotterdam Study^25^, the Leiden Longevity Study^26^ and the PROspective Study of Pravastatin in the Elderly at Risk^27^ (Fig. 1c), and investigated their clinical utility.

## Results

### Study population and the metabolomic state model

Based on the UK Biobank cohort^22,28^, we derived an integrated metabolomic state capturing information on incident disease risk in a general population sample (Fig. 1a,b). We extracted clinical predictors and disease endpoints for 117,981 individuals with serum NMR profiling at the time of cohort recruitment (Methods and Supplementary Tables 1–3). The study population had a median age of 58 years (interquartile range (IQR) 50, 63), of whom 54.2% were female, 11% current smokers and 5.2% diagnosed with T2D (Table 1). Median body mass index (BMI) was 26.8 (IQR 24.2, 29.9), systolic blood pressure was 136 mmHg (IQR 124, 149), total cholesterol was 5.65 mmol l^–1^ (IQR 4.90, 6.42) and glucose was 4.93 mmol l^–1^ (IQR 4.60, 5.32). Median follow-up was 12.2 years with ~1,435,340 overall person-years. To maximize the generalizability and transferability of our results, we partitioned the data spatially by the 22 recruitment centers. For each center, all individuals from a single center were retained for testing of models that were trained on individuals pooled from the 21 remaining recruitment centers and then randomly split into training and validation sets to develop the models. After model selection on the validation datasets and obtaining the selected models’ final predictions on the individual test sets, test set predictions were aggregated for downstream analysis (Fig. 1b).

**Table 1:** The study population

Characteristic	Male, *n* = 54,078^a^	Female, *n* = 63,903^a^	Overall, *n* = 117,981^a^
Age at recruitment	58 (50, 64)	57 (50, 63)	58 (50, 63)
Education years	15.00 (11.00, 15.00)	13.00 (11.00, 15.00)	13.00 (11.00, 15.00)
Current smoker	6,724 (12%)	5,747 (9.0%)	12,471 (11%)
Daily alcohol intake	13,651 (25%)	10,191 (16%)	23,842 (20%)
Daily moderate to vigorous physical activity	50 (15, 105)	45 (10, 90)	45 (10, 90)
Daily healthy food	52,974 (98%)	63,290 (99%)	116,264 (99%)
Family history of diabetes	8,827 (16%)	11,266 (18%)	20,093 (17%)
T2D	3,882 (7.2%)	2,295 (3.6%)	6,177 (5.2%)
Weight (kg)	84 (76, 94)	69 (62, 79)	76 (66, 88)
Standing height (cm)	176 (171, 180)	162 (158, 167)	168 (162, 175)
BMI	27.3 (25.0, 30.1)	26.1 (23.5, 29.7)	26.8 (24.2, 29.9)
Waist/hip ratio	0.93 (0.89, 0.98)	0.81 (0.77, 0.86)	0.87 (0.80, 0.94)
Waist circumference (cm)	96 (89, 103)	83 (76, 92)	90 (80, 99)
Systolic blood pressure (mmHg)	139 (128, 152)	133 (121, 147)	136 (124, 149)
Total cholesterol (mmol l^–1^)	5.45 (4.70, 6.21)	5.80 (5.07, 6.58)	5.65 (4.90, 6.42)
LDL cholesterol (mmol l^–1^)	3.46 (2.87, 4.05)	3.56 (3.00, 4.17)	3.52 (2.94, 4.12)
HDL cholesterol (mmol l^–1^)	1.24 (1.06, 1.45)	1.55 (1.32, 1.82)	1.40 (1.17, 1.67)
Triglycerides (mmol l^–1^)	1.69 (1.18, 2.44)	1.33 (0.96, 1.89)	1.48 (1.04, 2.14)
Glucose (mmol l^–1^)	4.96 (4.61, 5.37)	4.91 (4.59, 5.28)	4.93 (4.60, 5.32)
Glycated hemoglobin (%)	35.3 (32.8, 38.1)	35.2 (32.7, 37.7)	35.2 (32.8, 37.9)
Creatinine (umol l^–1^)	80 (72, 88)	63 (57, 70)	70 (61, 81)
Cystatin C (mg l^–1^)	0.92 (0.84, 1.01)	0.86 (0.78, 0.95)	0.88 (0.80, 0.98)
Urea (mmol l^–1^)	5.45 (4.68, 6.33)	5.10 (4.33, 5.95)	5.26 (4.49, 6.13)
Urate (umol l^–1^)	350 (305, 399)	264 (225, 309)	303 (250, 361)
Aspartate aminotransferase (U l^–1^)	26 (23, 31)	23 (20, 27)	24 (21, 29)
Alanine aminotransferase (U l^–1^)	24 (18, 32)	18 (14, 23)	20 (15, 27)
Alkaline phosphatase (U l^–1^)	79 (67, 93)	82 (67, 98)	80 (67, 96)
Albumin (g l^–1^)	45.52 (43.80, 47.24)	44.91 (43.21, 46.63)	45.20 (43.47, 46.93)
C-reactive protein (mg l^–1^)	1.29 (0.67, 2.55)	1.38 (0.65, 2.95)	1.33 (0.66, 2.76)
Erythrocytes (10^12^ cells l^–1^)	4.74 (4.51, 4.98)	4.32 (4.10, 4.54)	4.50 (4.23, 4.79)
Leukocytes (10^9^ cells l^–1^)	6.68 (5.66, 7.89)	6.61 (5.60, 7.81)	6.64 (5.62, 7.85)
Platelets (10^9^ cells l^–1^)	234 (202, 269)	261 (226, 301)	248 (214, 287)
Hemoglobin (g dl^–1^)	15.00 (14.37, 15.64)	13.50 (12.90, 14.10)	14.15 (13.31, 15.02)
Hematocrit (%)	43.3 (41.4, 45.2)	39.2 (37.5, 41.0)	41.0 (38.7, 43.5)
Mean corpuscular volume (fl)	91.4 (88.8, 94.1)	91.1 (88.4, 93.7)	91.2 (88.6, 93.9)
Mean corpuscular hemoglobin (pg)	31.69 (30.70, 32.70)	31.37 (30.33, 32.37)	31.50 (30.50, 32.50)
Mean corpuscular hemoglobin (g dl^–1^)	34.60 (34.00, 35.22)	34.36 (33.80, 35.00)	34.48 (33.90, 35.10)
Antihypertensives	1,090 (2.0%)	680 (1.1%)	1,770 (1.5%)

^a^Median (IQR); *n* (%)

We externally validated disease-specific metabolomic states in four independent cohorts analyzed with the same 1H-NMR metabolomics assay, the Whitehall II cohort^24^, and three independent cohorts of the BBMRI-NL consortium (Fig. 1c). The Whitehall II cohort^24^ is an ongoing prospective cohort study, including metabolomics for 6,197 participants aged 44–69 years. The Rotterdam Study is a prospective, population-based cohort study among individuals living in the Ommoord district in the city of Rotterdam (the Netherlands)^25^, offering metabolomics for 2,949 participants with a median age of 74 years (IQR 70–79). The Leiden Longevity PAROFF Study (LLS)^26^ comprises offspring and spouses of long-lived individuals, with metabolomics available for 1,655 individuals with a mean age of 59 years (IQR 54–63). Finally, the PROspective Study of Pravastatin in the Elderly at Risk (PROSPER) is a clinical trial investigating pravastatin effects^27^, of which 960 samples with a median age of 76 years (IQR 73–78) are included in the BBMRI-NL platform. Detailed characteristics of the four replication cohorts are presented in Supplementary Data and Supplementary Table 4.

**Extended Data Fig. 1:**
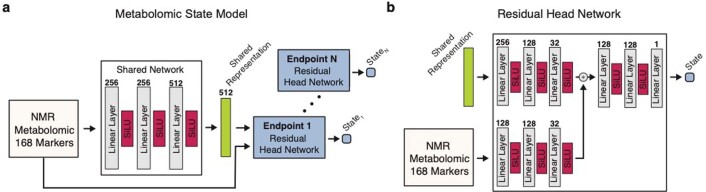
Details of the metabolomic state model. **a)** Overview of the residual architecture of the metabolomic state model. 168 circulating metabolomic markers are fed to the shared trunk network to learn a common shared representation. Endpoint-specific head networks then predict the metabolomic state for each endpoint from the shared representation and the input using a residual connection. **b)** Details of the residual head network. The model architecture is described in detail in (Methods Section ‘Metabolomic state model’).

**Extended Data Fig. 2:**
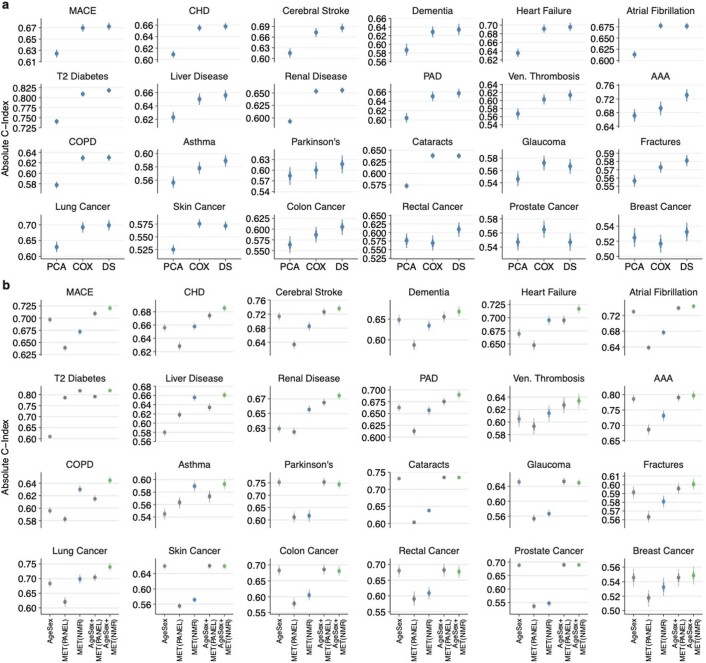
The metabolomic state model outperforms linear baselines on NMR-derived metabolite profiles, and NMR-derived metabolite profiles are more predictive than PANEL metabolites. **a)** Displayed are C-indices for the Cox Proportional Hazards models trained on the metabolomic state (MET), the 168 metabolites (CPH) as well as on the first ten components of a PCA-reduction of the 168 metabolites (PCA) for each of the 24 investigated endpoints. The metabolomic state performs comparably or better than both the CPH and PCA models for all endpoints, except prostate cancer. **b)** Displayed are C-indices for Cox Proportional Hazards models trained on Age+Sex (Age+Sex), the metabolomic states derived from NMR metabolomics (MET(NMR)), the metabolomic states derived from the PANEL metabolites (MET(PANEL)) and combinations of Age+Sex and the metabolomic states respectively. NMR profiles provide predictive information comparable or superior to the PANEL metabolites for all investigated endpoints, also reflected in the predictive performance over the Age+Sex covariates. The MET(PANEL) set included albumin, cholesterol, HDL and LDL cholesterol, triglycerides, glucose, and creatinine. Statistical measures were derived from n = 117.981 individuals. Individuals with prior events were excluded (Supplementary Table 1). Data are presented as median (center of error bar) and 95% CI (line of error bar) determined by bootstrapping over 1000 iterations. PAD - Peripheral Artery Disease, AAA - Abdominal Aortic Aneurysm, COPD - Chronic Obstructive Pulmonary Disease.

The metabolomic state model is a multitask residual neural network trained on the entire set of 168 original metabolomic markers to model the integrative metabolomic state for all 24 endpoints simultaneously (Fig. 1b, Extended Data Fig. 1 and Metabolomic state model). This allowed us to leverage the shared metabolite profiles while retaining flexibility in fitting endpoint-specific variations, outperforming endpoint-specific linear models and linear models on principal components (Extended Data Fig. 2).

To test whether multidisease states could be equally informative from readily accessible information from study participants at baseline, we investigated three different scenarios with increasingly comprehensive predictor sets. First, we considered age and sex only, both highly predictive for common diseases and available at no cost. Second, we investigated cardiovascular predictors from well-validated primary prevention scores, the American Heart Association (ASCVD)^3^, which are easily accessible at minimal cost and are predictive beyond cardiovascular disease, including neurological and neoplastic conditions^29–31^. Third, we extended these predictors with a comprehensive set of clinical predictors beyond what is typically available in primary care. These included >30 predictors with information on lifestyle factors, physical measurements and laboratory values, as well as further validated disease-specific predictors from FINDRISC^32^ (T2D) and CAIDE^33^ (dementia) scores (Fig. 1d and Supplementary Table 2).

### Metabolomic state stratifies the risk of disease onset

**Fig. 2:**
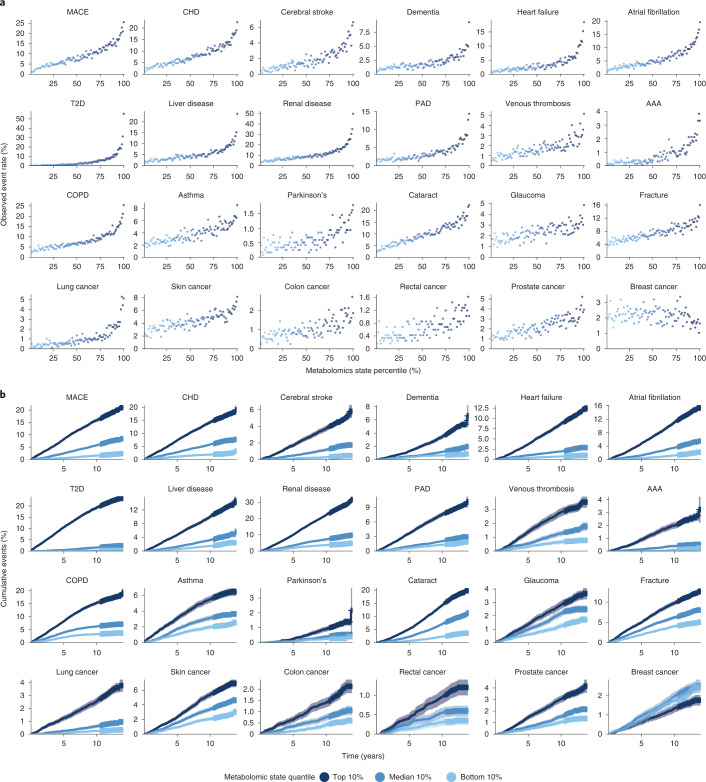
Metabolomic state is associated with ORs and stratifies survival. **a**, Observed event frequency for incident disease plotted against metabolomic state percentiles over the entire study population for all 24 endpoints. **b**, Cumulative event rates over the observation time for all assessed endpoints, stratified by metabolomic state quantiles (light blue, bottom 10%; blue, median 10%; dark blue, top 10%), with 95% CIs indicated. PAD, peripheral artery disease.

A critical component of prevention is identification of individuals at high risk of developing a disease, often at an early subclinical stage. To investigate whether the NMR-derived metabolomic state informs disease risk, we assessed the link with incident event rates in the observation period (Fig. 2a). To allow comparison between the endpoints despite the large differences in event rates (Supplementary Table 7; for example, Parkinson’s disease, 0.6%; major adverse cardiac event (MACE), 8.7%), we also calculated the observed event rate ratio between individuals in the top and bottom 10% of metabolomic states (Fig. 2 and Supplementary Table 7) with 95% confidence intervals (CIs).

We observed increasing event rates over metabolomic state percentiles for all 24 investigated diseases, except breast cancer. For 15 of the 24 diseases, the top 10% of the metabolomic state corresponded to a rate more than fivefold higher compared with the bottom 10%. For conditions such as T2D (top 10%, 21.87%; bottom 10%, 0.36%; odds ratio (OR) 61.45, 95% CI 47.00, 86.12), abdominal aortic aneurysm (AAA) (top 10%, 2.46%; bottom 10%, 0.18%; OR 14.1, 95% CI 9.93, 24.45) and heart failure (top 10%, 10.80%; bottom 10%, 0.96%; OR 11.27, 95% CI 9.43, 13.50) the ratio was >10. Ratios for most other diseases were lower—for example, cerebral stroke 9.66 (95% CI 7.64, 12.14), MACE 9.25 (95% CI 8.12, 10.53), atrial fibrillation 8.13 (95% CI 6.95, 9.37), all-cause dementia 6.39 (95% CI 5.40, 8.09) or chronic obstructive pulmonary disease (COPD) 4.98 (95% CI 4.37, 5.62). In contrast, we observed much smaller ratios for some diseases—for example, glaucoma (top 10%, 3.47%; bottom 10%, 1.57%; OR 2.19, 95% CI 1.91, 2.62) or asthma (top 10%, 5.52%; bottom 10%, 2.48%; OR 2.22, 95% CI 2.01, 2.57), thus suggesting less information contained in the respective metabolomic states. In summary, the disease-specific metabolomic state stratified risk trajectories for all investigated endpoints except breast cancer (Fig. 2b), separating the rates of cumulative events most notably for T2D, renal disease and heart failure but also, to a much lesser extent, for glaucoma or asthma.

### Information is shared with clinical predictors

Many clinical predictors are readily available in primary care and commonly used to stratify the risk of common diseases such as cardiovascular disease^3^, kidney disease^34^ or diabetes^32^. While more complex risk scores have been proposed^35^, the trade-off between the added predictive information and resources in time and cost required to collect the new data has limited clinical adoption^36^. We therefore investigated the predictive information of the relatively affordable and standardized NMR metabolomics assay against common clinical variables in the UK Biobank and in four independent validation cohorts.

**Fig. 3:**
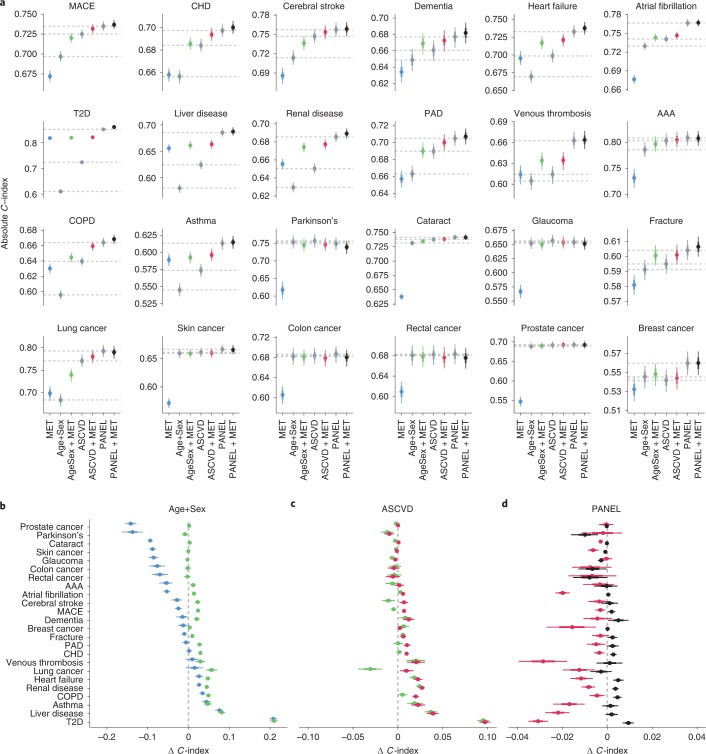
Predictive value of the metabolomic state is endpoint dependent. **a**, Comparison of discriminative performance of CPH models trained on the metabolomic state only (MET), the three clinical predictor sets (Age+Sex, ASCVD and PANEL) and the sets’ combinations with the metabolomic state. Horizontal dashed lines indicate the median performance of the three clinical predictor sets. **b**, Differences in discriminative performance between the Age+Sex baseline (dashed line), metabolomic state only (blue) and the combination of Age+Sex and metabolomic state (green). **c**, Differences in discriminative performance between ASCVD predictors (dashed line), the combination of Age+Sex and the metabolomic state (green) and the combination of metabolomic state and ASCVD predictors (red). **d**, Difference in discriminative performance between comprehensive PANEL predictors (dashed line), ASCVD + MET (red) and PANEL + MET (black). **a**–**d**, Statistical measures were derived from *n* = 117.981 individuals; those with previous events were excluded (Supplementary Table 1). Data are presented as median (center of error bar) and 95% CI (line of error bar) determined by bootstrapping of with 1,000 iterations. **b**–**d**, The *x-*axis range differs across panels; vertical grid lines indicate differences of 0.02 *C*-index.

**Extended Data Fig. 3:**
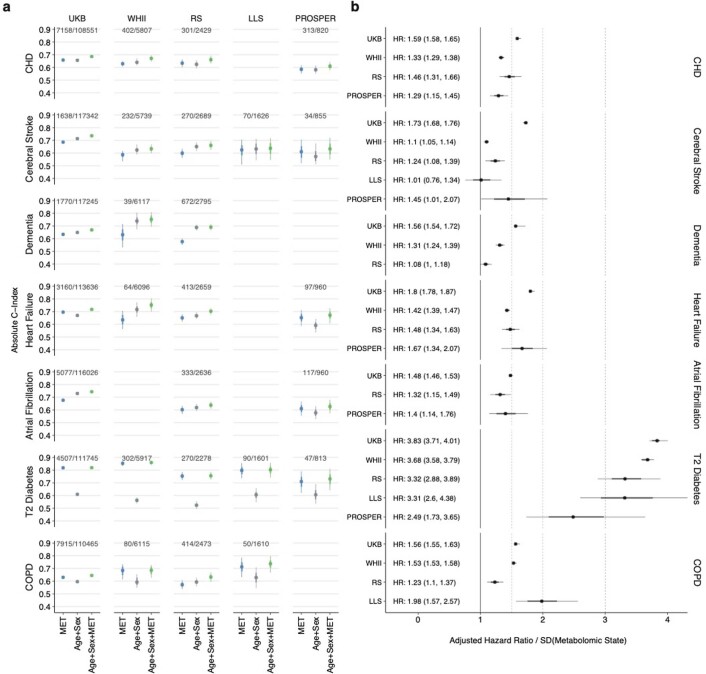
External validation in four independent cohorts. **a)** Displayed are discriminative performances described by the C-index for UK Biobank and the four external validation cohorts, Whitehall II (WHII), Rotterdam Study (RS), Leiden Longevity Study (LLS), and the PROSPER trial (PROSPER). CPH models were trained on the metabolomic state model (MET) as fitted on UK Biobank and applied to each cohort, as well as on Age+Sex and Age+Sex+MET. The metabolomic state is predictive in the replication cohorts for all assessed endpoints. Dots indicate the median performance, while whiskers indicate the 95% confidence interval (CI) determined by bootstrapping over 1000 iterations. **b)** Age+Sex adjusted hazard ratios (HRs) for the metabolomic state in all five cohorts. A unit standard deviation increase in the metabolomic state corresponds to an HR increase in predicted risk. Statistical measures were derived from n = 6.117 (Whitehall II), n = 2949 (Rotterdam Study), n = 1655 (Leiden Longevity Study), and n = 960 (PROSPER) individuals as indicated. Data are presented as median (center of error bar) and 95% CI (line of error bar) determined by bootstrapping over 1000 iterations.

**Extended Data Fig. 4:**
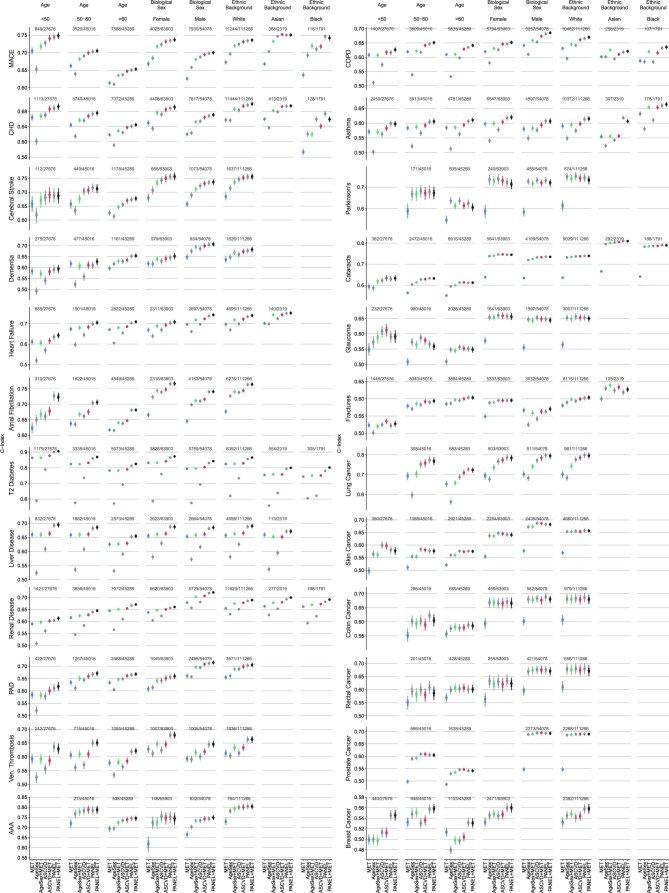
The discriminative performance is largely comparable over multiple subgroups. Discriminative performance is stratified by endpoint, age at recruitment, biological sex, and self-reported ethnic background. As the concordance index is only reliable if a sufficient number of events are recorded, subgroups with < 100 events were excluded. The number of events and eligible individuals is indicated at the top of each panel. Data are presented as median (center of error bar) and 95% CI (line of error bar) determined by bootstrapping with 1000 iterations.

First, we modeled disease risk for each endpoint in the UK Biobank using CPH models for three clinical predictor sets with increasing complexity: Age+Sex, highly predictive and available ahead of any test; ASCVD, a set of readily available cardiovascular predictors; and PANEL, a comprehensive selection of clinical predictors including in-depth blood measurements (Fig. 1d) exceeding those typically available in primary care. For all sets, the performance of CPH models was benchmarked against those based on the sets’ combinations with the metabolomic state. As quantified by Harrell’s *C*-index, the discriminative performances of all models at 10 years after baseline are shown in Fig. 3a. Subsequently, to validate metabolomic states, we applied the trained metabolomic state model to the external validation cohorts and replicated the CPH models with and without metabolomic state addition for the Age+Sex predictor set for all endpoints available. The results of the external validation are shown in Extended Data Fig. 3. We noted the discriminative performance of the metabolomic state to be highly disease dependent: while the metabolomic state contained significantly less predictive information than clinical predictors for cataract, glaucoma and skin, colon, rectal and prostate cancers, this was not the case for renal disease, liver disease and T2D. Here, the metabolomic state contained a greater predictive value than Age+Sex and even ASCVD. Generally, we observed an increase in discriminative performance with the addition of more comprehensive clinical predictors across all endpoints, and performances were stable over different age groups, biological sexes and ethnic backgrounds (Extended Data Fig. 4).

To better assess the predictive value of the metabolomic state (MET) in comparison with clinical variables, we calculated *C*-index deltas (Fig. 3b–d). We noted that CPH models fit solely on the metabolomic state performed competitively or better than Age+Sex for ten of the 24 endpoints, including T2D and COPD, but also for heart failure, liver disease and renal disease (Fig. 3b). The competitive performance compared with Age+Sex was replicated in the validation cohorts for T2D, COPD, heart failure, coronary heart disease (CHD) and all-cause dementia (Extended Data Fig. 3 and Supplementary Table 10).

**Extended Data Fig. 5:**
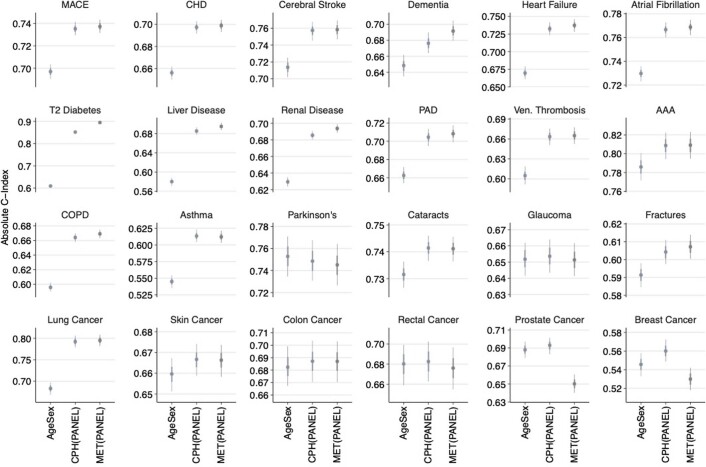
Comparison of the predictive performance of the PANEL predictors in a Cox proportional hazard model and the neural network. Comparison of discriminative performances of the CPH models and Metabolomic State Model (MSM) trained on the PANEL covariates. The discriminative performance of the PANEL predictors is either similar or can be further improved by modeling with the same architecture as the metabolomic state model for most (non-cancer) endpoints. Statistical measures were derived from n = 117.981 individuals. Individuals with prior events were excluded (Supplementary Table 1). Data are presented as median (center of error bar) and 95% CI (line of error bar) determined by bootstrapping with 1000 iterations.

Interestingly, CPH models fit on the combination of the metabolomic state with Age+Sex (Age+Sex + MET) performed comparably to, or better than, the ASCVD predictors for 15 of the 24 endpoints, including T2D, liver disease, renal disease, heart failure, venous thrombosis and dementia (Fig. 3c). While the comprehensive PANEL score generally contained the most predictive information, surprisingly we observed only modest gains over the combination of ASCVD and the metabolomic state, and Age+Sex and the metabolomic state (Fig. 3d). Applying the complex metabolomic state model architecture to the predictors of the PANEL, we did not observe systematic performance improvements (Extended Data Fig. 5).

### Discriminative improvements over clinical predictors

In addition to investigating the shared information, we were interested in quantifying the additive predictive value of metabolomic state over readily available clinical variables. To understand how the information is distributed over the PANEL predictors, we first assessed the aggregated coefficients of the CPH model and found that basic demographic information, medical history and physical measurements provided the most predictive information over all endpoints (Supplementary Table 5). In addition, apart from shared measures (for example, glucose, albumin or creatinine), lipids and creatinine/cystatin c, we did not observe strong correlations (|*r*| > 0.5) between the PANEL predictors and NMR metabolites (Supplementary Table 6). Therefore, we continued assessment of performance differences between the CPH models’ fit on clinical predictors and those with the added metabolomic state by calculating differences in the *C*-index (Supplementary Table 9).

In the UK Biobank, the metabolomic state significantly added predictive information over age and sex for 18 of the 24 endpoints; in contrast, endpoints with a comparably low predictive value of the metabolomic state, such as Parkinson’s disease, skin cancer, colon cancer, rectal cancer, glaucoma and cataract, did not benefit from the addition of the metabolomic state. Results from four external cohorts independently confirmed significant discriminative improvements over Age+Sex for CHD, heart failure, atrial fibrillation, T2D and COPD (for detailed results and event counts for the independent cohorts, see Extended Data Fig. 3a and Supplementary Table 10).

Beyond basic demographic predictors, addition of the metabolomic state to cardiovascular predictors further significantly improved discriminative performance for 15 of the 24 endpoints (Fig. 3c). Even when added to the comprehensive PANEL set, the metabolomic state provided significant additional discriminatory value for eight of the 24 endpoints (Fig. 3d) as quantified by *C*-index, including T2D (0.009, 95% CI 0.007, 0.012), dementia (0.005, 95% CI 0, 0.009), heart failure (0.005, 95% CI 0.003, 0.007), COPD (0.005, 95% CI 0.003, 0.006), renal disease (0.004, 95% CI 0.002, 0.005), CHD (0.003, 95% CI 0.001, 0.004) and MACE (0.002, 95% CI 0, 0.004).

**Extended Data Fig. 6:**
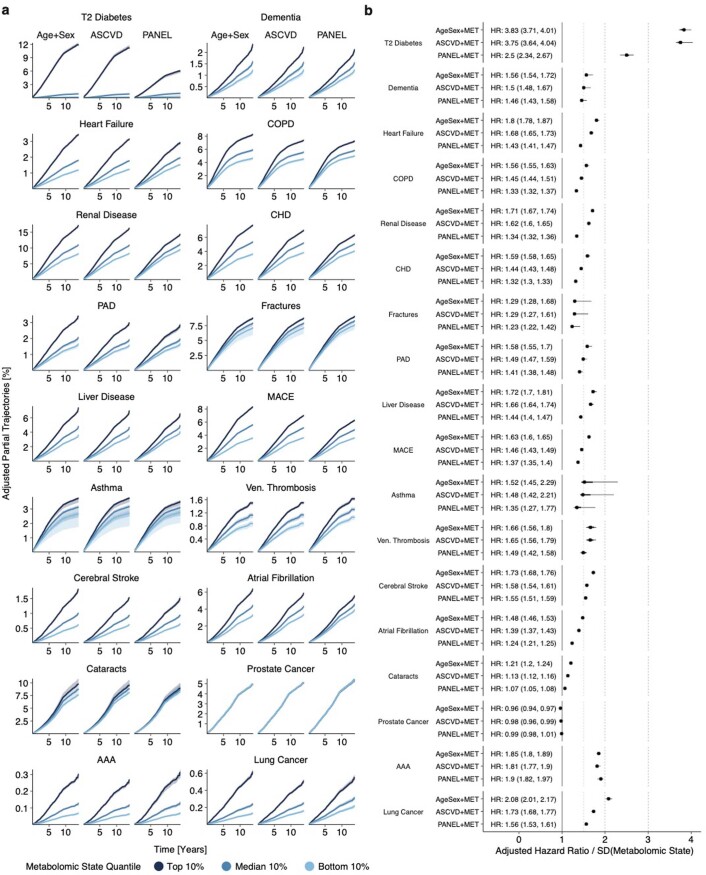
Adjusted effect of the metabolomic state is endpoint dependent. **a)** Adjusted trajectories representing the partial cumulative risk dependent on the metabolomic state over time for the endpoints where the metabolomic state added information to the Age+Sex baseline (see Fig. 3b) for the bottom (light blue), median (blue), and top (dark blue) 10% metabolomic state quantiles. The shaded area indicates the 95% confidence interval as estimated by bootstrapping over 1000 iterations. **b)** Adjusted hazard ratios (HRs) for the metabolomic state in combination with the three clinical predictor sets. A unit standard deviation increase in the metabolomic state corresponds to an HR increase in predicted risk. Statistical measures were derived from n = 117.981 individuals. Individuals with prior events were excluded (Supplementary Table 1). Data are presented as median (center of error bar) and 95% CI (line of error bar) determined by bootstrapping with 1000 iterations.

We further sought to understand the potential of the metabolomic state in regard to individual risk under consideration of established clinical predictors. Therefore, we examined the partial effects and hazard ratios (HRs, per s.d. metabolomic state, with 95% CI) of the CPH models trained on the combinations of the metabolomic state and predictor sets Age+Sex, ASCVD and PANEL (Extended Data Fig. 6a) for those 18 endpoints with discrimination benefits over the Age+Sex set. We observed a notable separation between the top, median and bottom 10% of the metabolomic state in 14 of the 18 endpoints when adjusted for more comprehensive clinical predictors (for HRs, see Extended Data Fig. 6b). A change of 1 s.d. in the metabolomic state for T2D resulted in substantially adjusted HRs (HR_Age+Sex_ 3.83 (95% CI 3.71–4.01), HR_PANEL_ 2.5 (95% CI 2.34–2.67)), which were replicated with adjustment for Age+Sex in the independent cohorts (Extended Data Fig. 3b). Other investigated endpoints, such as all-cause dementia (HR_Age+Sex_ 1.56 (95% CI 1.54–1.72), HR_PANEL_ 1.46 (95% CI 1.43–1.47)), heart failure (HR_Age+Sex_ 1.8 (95% CI 1.74–1.86), HR_PANEL_ 1.45 (95% CI 1.38–1.52)), COPD (HR_Age+Sex_ 1.56 (95% CI 1.53–1.6), HR_PANEL_ 1.35 (95% CI 1.31–1.39)) or MACE (HR_Age+Sex_ 1.63 (95% CI 1.58–1.69), HR_PANEL_ 1.4 (95% CI 1.33–1.46)), showed less pronounced, yet clear, separation of risk trajectories. In regard to T2D, the HRs of the metabolomic states were externally validated with adjustment for Age+Sex for all-cause dementia, heart failure, atrial fibrillation, CHD and COPD (Extended Data Fig. 3b). In contrast, the metabolomic state only marginally modified the risk trajectories for asthma (HR_Age+Sex_ 1.37 (95% CI 1.3–1.44), HR_PANEL_ 1.09 (95% CI 1.03–1.16)) and cataract (HR_Age+Sex_ 1.22 (95% CI 1.18–1.25), HR_PANEL_ 1.08 (95% CI 1.05–1.11)).

### Discriminative performance translates to clinical utility

**Fig. 4:**
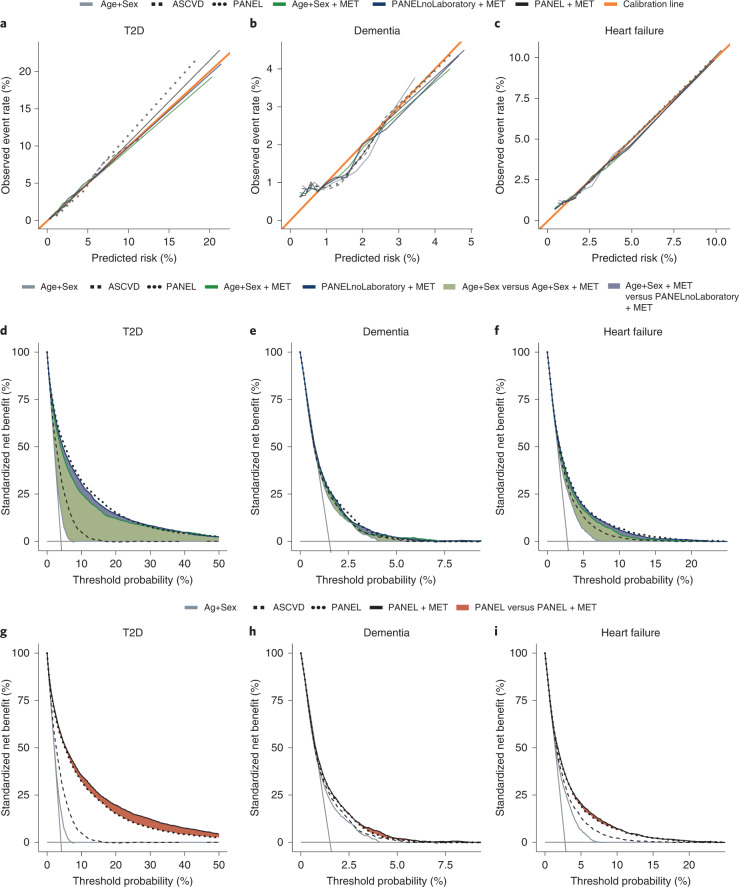
Model calibration and additive predictive value of the metabolomic state translate to potential clinical utility. **a**–**c**, Calibration curves for CPH models, including baseline parameter sets Age+Sex, ASCVD and PANEL, as well as their combinations with the metabolomic state (Age+Sex + MET) for the endpoints T2D (**a**), dementia (**b**) and heart failure (**c**). **d**–**f**, Endpoint-specific net benefit curves standardized by endpoint prevalence, where horizontal solid gray lines indicate ‘treat none’ and vertical solid gray lines indicate ‘treat all’; T2D (**d**), dementia (**e**) and heart failure (**f**). The standardized net benefits of sets Age+Sex, ASCVD and PANEL are compared with Age+Sex + MET and additional non-laboratory predictors of PANEL (PANELnoLaboratory). Green and blue color-filled areas indicate the added benefit of the combination of the metabolomic state and Age+Sex and PANELnoLaboratory, respectively. **g**–**i**, Standardized net benefit curves comparing the performance of PANEL + MET against baselines Age+Sex, ASCVD and PANEL; T2D (**g**), dementia (**h**) and heart failure (**i**). Decision curves were derived from *n* = 111,745 (T2D), *n* = 117,245 (dementia) and *n* = 113,636 (heart failure) individuals.

While discrimination is critical, the clinical utility of any risk model depends on calibration and the choice of adequate thresholds for interventions. We found all models well calibrated in the UK Biobank Cohort (see Fig. 4a–c and Supplementary Fig. 1 for details on all endpoints). UK Biobank^37^, as one of the largest and most comprehensive population cohorts in the world, therefore, allowed us to estimate clinical utility with high precision over a wide range of clinically reasonable intervention thresholds. However, adequate clinical decision thresholds directly depend on the benefits and harms of interventions and disease prevalence. We therefore calculated decision curves^38^ to estimate the benefit of adding metabolomic information to a prediction model (see Fig. 4d–i and Supplementary Fig. 1 for details on all endpoints). Further, we calculated clinically relevant metrics such as sensitivity, positive predictive value and positive likelihood ratio over multiple false-positive rates (Supplementary Table 11)^39^.

Specifically, we investigated the application of the metabolomic state in two scenarios. First, as a potentially economical and practical option, we assessed the combination of the metabolomic state with Age+Sex and with the less resource-intensive, non-laboratory predictors of the PANEL set. Second, we combined the metabolomic state with the entire PANEL set (including all laboratory predictors) to assess whether there is a net benefit even beyond comprehensive predictors.

**Extended Data Fig. 7:**
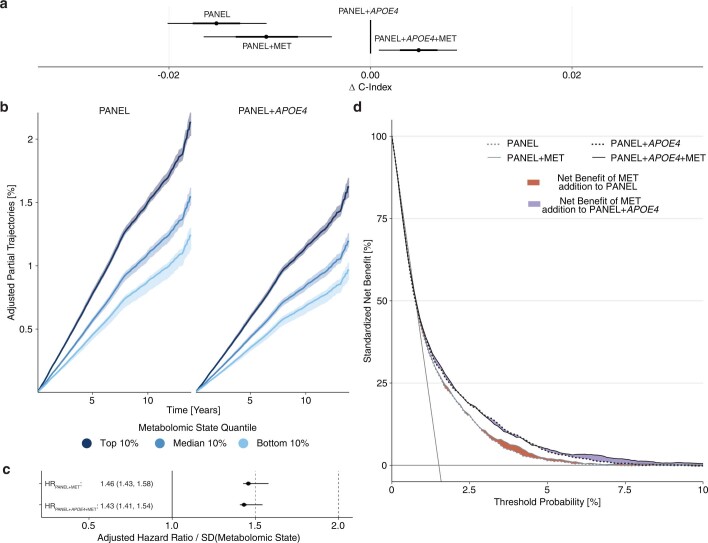
The metabolomic state contains independent predictive information over the *APOE4* carrier status for all-cause dementia. **a)** Displayed are C-index deltas between the CPH model trained on the PANEL + *APOE4* predictor set, its metabolomic state addition (PANEL + *APOE4* + MET), and CPH models trained on the PANEL set and its respective metabolomic state addition (PANEL + MET). The metabolomic state adds predictive information over the PANEL + *APOE4*. **b)** Partial trajectory for MET deciles (Top, Median, Bottom 10%) adjusted for PANEL and PANEL + *APOE4*, respectively. **c)** Hazard Ratio for the Metabolomic State adjusted for the predictors of the PANEL and PANEL + *APOE4*. **d)** Decision curve analysis for PANEL/PANEL + MET and PANEL + *APOE4*/PANEL + *APOE4* + MET. The areas in between the solid and dotted lines indicate added net benefits resulting from metabolomic state addition to PANEL (gray lines, red area) and PANEL + *APOE4* (black lines, violet area), respectively. Adding MET to PANEL improves net population benefit between the 2–8% decision threshold. In the case of PANEL + *APOE4*, MET addition improves utility at thresholds between 5–10%. Statistical measures were derived from n = 117.245 individuals without dementia at recruitment. Data are presented as median (center of error bar) and 95% CI (line of error bar) determined by bootstrapping with 1000 iterations.

Generally we found that discriminative gains (Fig. 3) translated to utility gains (see Fig. 4d–i and Supplementary Fig. 1 for details on all endpoints). The metabolomic state substantially added to age and sex for most endpoints, and additional non-laboratory predictors either closed (12 of the 24 endpoints, including T2D, stroke, heart failure and lung cancer) or narrowed the gap (an additional four of the 24 endpoints, including dementia, atrial fibrillation and renal disease) with the comprehensive set of PANEL predictors. The addition of the metabolomic state to the comprehensive PANEL predictors led to further improvements in the utility for reasonable ranges of decision thresholds for 11 of the 24 endpoints (most notably T2D, heart failure and, to a lesser extent, dementia; see Supplementary Fig. 1 for details on all endpoints and Extended Data Fig. 7 for additional analyses investigating apolipoprotein 4 (*APOE4*) carrier status for dementia). Conversely, where there were no improvements in the discriminatory value, no relevant improvements in clinical utility could be found. These observations were further reflected in the positive predictive values and positive likelihood ratios (Supplementary Table 9).

### Identification of disease-specific metabolite profiles

A requirement for the adoption of neural networks in medicine is explainability. While neural networks are not inherently interpretable, methods have been developed to overcome this challenge^40^. To identify which metabolites most affect disease risk, we approximated Shapely additive explanation (SHAP) values^41^ for all investigated diseases. Generally, the larger the absolute SHAP value the more important a metabolite for an individual prediction. Based on the direction of the effect of a metabolite’s contribution, increasing or decreasing the predicted risk, SHAP can take a positive or negative value.

**Fig. 5:**
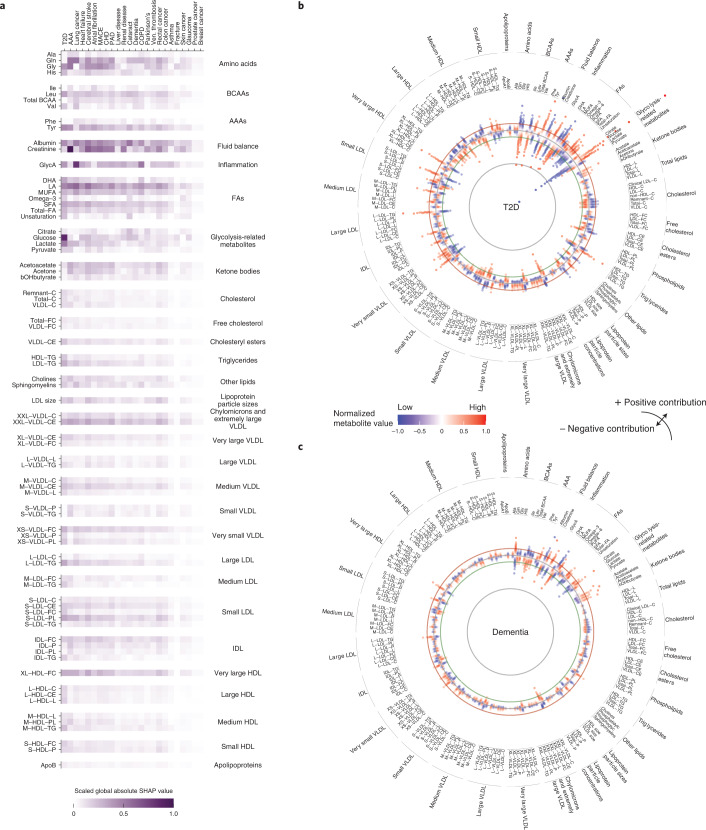
Analysis of the metabolomic state informs on metabolite profiles associated with disease risk. **a**, Heatmap showing the importance of metabolites in regard to the estimated metabolomic states, represented by absolute global SHAP value estimates per endpoint for the 75 globally most important metabolites. Endpoints are sorted by the discriminative performance of the metabolomic state (left to right; Fig. 3a). **b**, Global metabolite attributions for T2D; individual attributions are aggregated by percentiles and each dot indicates one percentile. The more distant a dot from the circular baseline, the stronger the absolute attribution for that percentile. Deviations toward the center and periphery represent negative and positive contributions, respectively, to the metabolomic state. Colors indicate the metabolite’s mean plasma value. **c**, Global metabolite attributions for all-cause dementia. IDL, intermediate-density lipoprotein.

**Extended Data Fig. 8:**
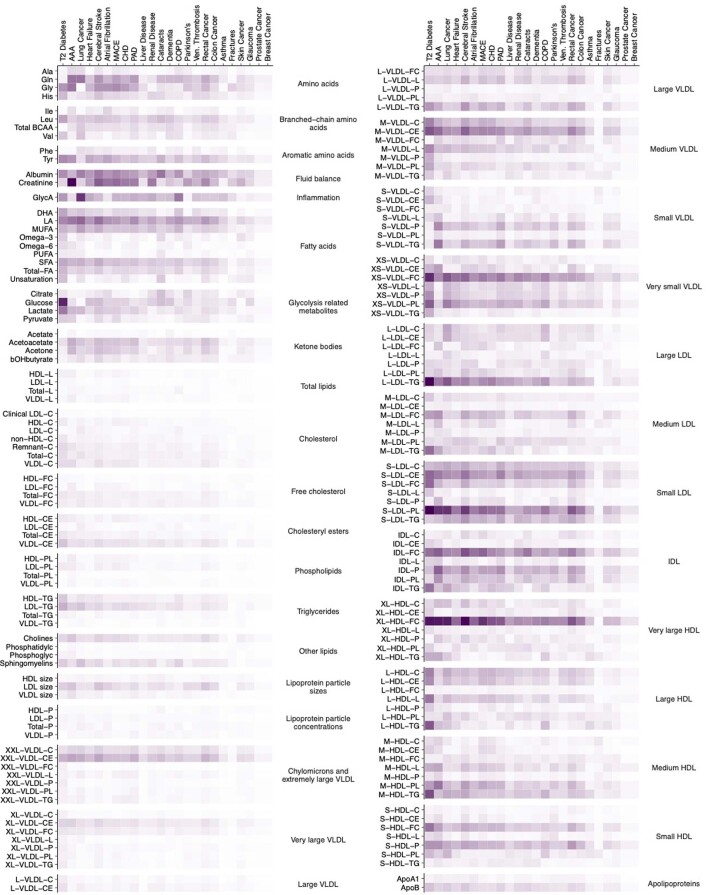
Global metabolite importances for each metabolite and endpoint. Heatmap of the metabolite importances, represented by absolute global SHAP value estimates per endpoint for the 168 circulating metabolites. The endpoints are sorted by the discriminative performance of the metabolomic state (left to right, see Fig. 3a). MACE - Major Adverse Cardiac Events, CHD - Coronary Heart Disease, PAD - Peripheral Artery Disease, AAA - Abdominal Aortic Aneurysm, COPD - Chronic Obstructive Pulmonary Disease.

To understand individual metabolites in the context of the 24 investigated diseases, we investigated global metabolite attributions, the sum of absolute SHAP values per metabolite and disease (Fig. 5a and Extended Data Fig. 8). We found that most high-impact metabolites were linked to multiple diseases: plasma levels of metabolites with consistently high contribution included the amino acids glutamine, glycine and tyrosine, metabolites related to carbohydrate metabolism, albumin, the kidney function marker creatinine, glycoprotein acetylation (GlycA) and the ketone bodies acetone and acetoacetate. Further implicated were fatty acids (FA) such as linoleic acid (LA) and multiple lipoprotein components, including free cholesterol in very large high-density lipoprotein (VHDL), triglycerides in large low-density lipoprotein (LDL), phospholipids in small LDL and sphingomyelins. In addition to shared metabolite profiles, we pinpointed marked associations of creatinine with AAA, glucose with T2D and GlycA with lung cancer and COPD. For diseases with a high discriminatory value for metabolomic state, predicted metabolite contributions were considerably higher than for diseases with little discriminatory metabolomic information (Fig. 5a).

Subsequently we focused on T2D (Fig. 5b) and all-cause dementia (Fig. 5c), two diseases with strong metabolomic contributions over the comprehensive clinical predictors and indications of clinical utility (see above). Metabolites related to carbohydrate metabolism, such as glucose and lactate, dominated the predicted metabolomic state of T2D in our model (Fig. 5b). In line with earlier findings^5,19,42^, we observed contributions of amino acids, ketone bodies, lipids and FAs as well as creatinine and albumin. We confirmed that higher plasma levels of FAs, docosahexaenoic acid (DHA) and LA were associated with lower risk^43,44^. Further, we observed a distinct contribution of lipid content across the whole density gradient of lipoproteins, including a high triglyceride content in LDL and IDL or free cholesterol content in very small very-low-density lipoprotein (VLDL) and HDL. For all-cause dementia, we identified creatinine, albumin and the amino acids glutamine, leucine and tyrosine as predominant contributors to predicted risk (Fig. 5c). In line with earlier findings^8,45^, we observed a notable role of FAs such as LA and monounsaturated and saturated FAs, as well as a protective effect of branched-chain amino acids (BCAAs). Our results further implicate associations of glucose, ketone bodies acetate, acetoacetate and acetone, and beta-hydroxybutyrate. Finally we found several lipoproteins to be associated, most notably free cholesterol in very large HDL and cholesterylester in extremely large VLDL. Comprehensive data for all investigated endpoints, including the most important metabolites and disease-specific attribution profiles, can be found in Extended Data Fig. 8, Supplementary Table 12 and Supplementary Fig. 2).

**Extended Data Fig. 9:**
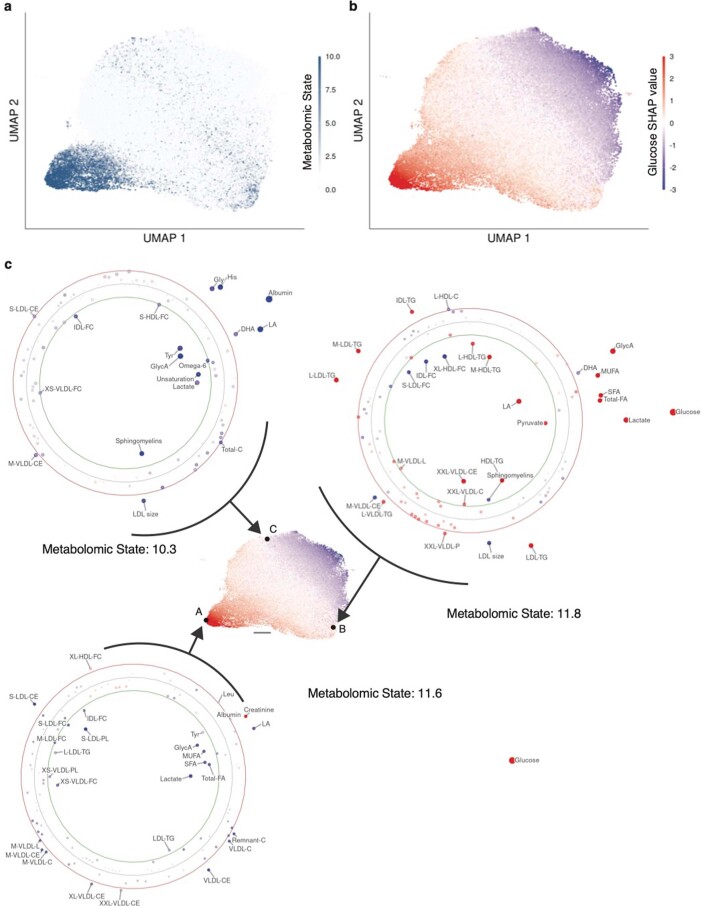
Individual attribution profiles diverge for high-risk individuals in T2D. The UMAP projection allows an assessment of the complex, high-dimensional manifold of attribution values in 2-dimensional space. For visualization, 41 unconnected outliers of 117981 total observations were excluded. **a)** UMAP of the SHAP value metabolite attributions for T2D for the entire study population colored by each individual’s metabolomic state. **b)** The same UMAP colored by the Glucose SHAP value. **c)** Displays individual attribution profiles for three high-risk (metabolomic state > 10, top 1% metabolomic state percentile) individuals, indicated by the letters A, B, C in the central UMAP. The three individual attribution profiles are dominated by different metabolites. The scale bar represents a unit in the UMAP space. The individual attribution profiles are set up equivalently to Figure 6: Each point in an individual attribution profile indicates one metabolite; the position, size, and color of the point indicate the magnitude and direction of the attributed contribution to predicted risk. The green and red circles represent the bounds of the top and bottom percentile of the global SHAP distribution, respectively, indicating outliers in the SHAP global distribution.

**Extended Data Fig. 10:**
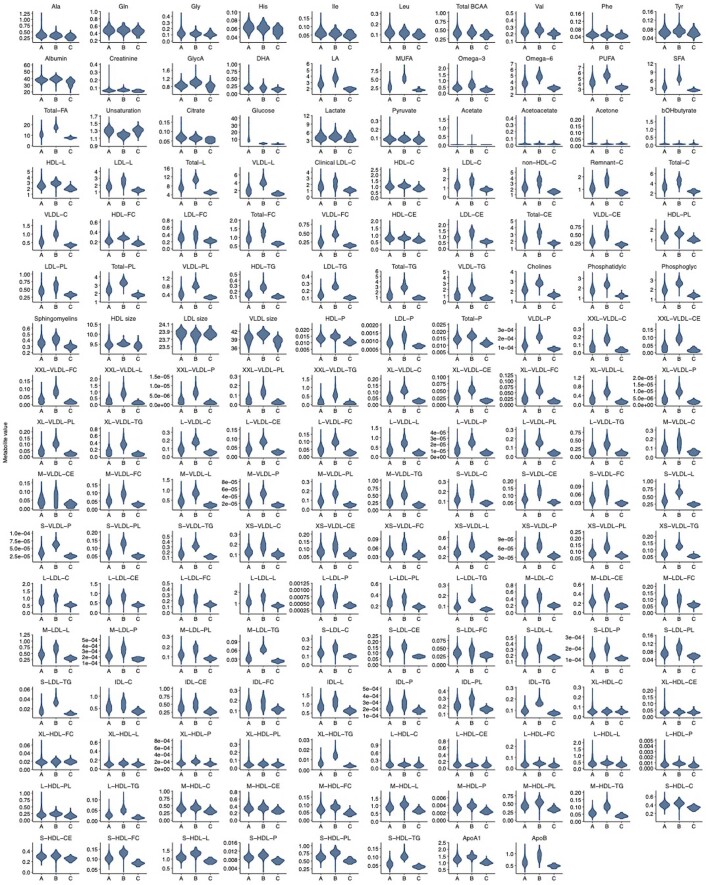
Metabolites differ throughout the attribution space. Displayed are distributions for all measured metabolites (n = 168) stratified by the region (A, B, and C) in the attribution space, defined by the UMAP of the attributions for T2D (see Extended Data Figure 9c). Regions were defined by including all samples with an euclidean distance < 1 to the centroid A, B, and C, respectively; a Euclidean distance of 1 is indicated by the scale bar (see Extended Data Figure 9c). The distributions differ notably for metabolites, including glucose, fatty acids (that is LA and Omega-6), and multiple lipoprotein components (that is VLDL cholesterol and very large HDL triglycerides).

Computation of SHAP values also allowed us to derive individual risk attribution profiles for individual participants and diseases, informing on the impact of single metabolites on a given prediction. We visualize the attribution profiles for T2D in two-dimensional uniform manifold approximation and projection (UMAP)^46^ space (Extended Data Fig. 9), which is resolved by the estimated importance of glucose (that is, SHAP values assigned to glucose regarding the predicted risk for T2D; Extended Data Fig. 9a). While most high-risk individuals (top 1% metabolomic state) are located at coordinates with strong glucose attribution, we found high-risk individuals scattered over the entire attribution space (Extended Data Fig. 9b). Interestingly, the attribution profiles of high-risk individuals were not consistently dominated by glucose but rather by, for instance, low levels of albumin, LA, DHA, histidine and glycine (Extended Data Fig. 9c). This observation is further reflected in NMR metabolite concentrations, because we found substantial differences in the concentrations of glucose, LA, FAs and triglycerides when comparing the metabolite distributions of individuals in the area with the strongest glucose attribution with those of individuals in two spatially distinct, high-risk UMAP areas (Extended Data Fig. 10).

## Discussion

The assessment of risk is a critical component of disease prevention. However, comprehensive risk assessment often requires the careful acquisition of predictors, one disease at a time. Thus, for each disease-specific risk score, the resources (time and cost) required for the collection can severely limit adoption and utility^47^. Interestingly, many common diseases involve metabolic alterations and human blood metabolomic patterns contain rich systemic information on the underlying physiology^9–11,20,21^. While individual metabolites have long been linked to disease risk, systemic information from blood metabolomics could inform on multiple diseases simultaneously. Importantly, in recent years, assays such as 1H-NMR spectroscopy have matured and allowed the assessment of serum metabolite information robustly at comparatively low cost^13,14^. However, the potential of metabolomic profile as a single-domain, multidisease assay in primary care has not been investigated thus far.

We have assessed the potential of NMR-derived metabolomic profiles as a tool for individualized prediction of onset across 24 common diseases. With >1.4 million person-years of follow-up, we leveraged the systemic information in metabolomic profiles to derive integrative metabolomic states for many diseases simultaneously. We found the metabolomic states to be predictive for all but one of the investigated diseases and externally validated these findings in four independent cohorts for available endpoints. Further, we investigated the predictive value beyond clinical variables and identified a subset of endpoints with potential clinical utility. Finally, we examined metabolite attributions confirming a multitude of disease-associated metabolites and a shared metabolomic background of common diseases.

Importantly, we found that the predictive information of the metabolomic state matched established clinical variables for many of the investigated endpoints. In line with previous reports on NMR–metabolite associations, we confirm that metabolomic profiles are highly predictive for, for example, T2D^19^, dementia^8^ and cardiovascular diseases^6,11,17^ such as CHD and heart failure^48^. Generally, the additional predictive information decreased over comprehensive clinical predictors, indicating that substantial parts of the metabolomic state’s discriminatory information are shared with established clinical predictors. However, for multiple endpoints, including T2D, all-cause dementia and heart failure, the metabolomic state contained complementary information that added predictive value even over comprehensive laboratory measurements. These findings largely translate into potential clinical utility for NMR-based metabolomic profiling, both as a replacement for comprehensive laboratory examinations and as an additional source of discriminatory information to refine comprehensive risk assessments for multiple diseases simultaneously.

Calculation of attributions for each individual allowed us to assess how differences in the metabolomic profile affect disease risk. We confirmed the role of metabolites such as albumin and creatinine, which have previously been associated with all-cause and disease-specific mortality^11,16^ and are already part of routine care^49,50^. Further, we confirmed the role of LA, tyrosine, glycine and cholesterylesters in extremely large VLDL in multiple diseases, further supporting metabolomic multidisease-spanning information^21^. Dissecting disease-specific attribution profiles, we found that metabolite attributions reflect metabolite–disease associations previously reported in the literature. In the case of T2D, we confirmed the associations between disease risk and metabolites beyond glucose. Specifically, our model captured the positive association between high levels of glycoprotein acetyls, BCAAs, lactate and FAs (both monounsaturated and saturated) and the protective role of metabolites such as LA or glycine^5,19^. In the attribution profile of dementia we replicated associations with BCAAs, including leucine and valine, and with FAs, most notably LA^8,45^. In addition, the associations of GlycAs with cardiovascular disease, T2D, COPD and lung cancer^51,52^ are reflected in the attributions. Consequently, our metabolomic state model learns systemic information in NMR-derived metabolomic profiles based on established shared and highly specific metabolite–disease associations.

In our perspective, 1H-NMR metabolomics profiling is an attractive candidate for a single-domain, multidisease assay. Because many countries already recommend regular check-ups entailing blood tests in the prevention of selected common diseases^53^, our results indicate the potential of NMR metabolomic profiling in combination with simple demographic, but also with comprehensive laboratory predictors to estimate disease risk. In addition, metabolomic risk profiles could be of potential value in the guidance of pharmacological and lifestyle interventions. This is especially relevant for diseases such as T2D, where interventions on modifiable risk factors have been shown to delay disease onset^54^ and prevent subsequent comorbidities^55,56^. Similarly, the Lancet 2020 commission suggested that up to 40% of worldwide dementia may be preventable by interventions on modifiable risk factors^57^. This is particularly compelling because today’s pharmacological treatment options for dementia are scarce. However, the efficacy of various lifestyle interventions^58,59^ is disputed, calling for further experimental investigation.

Before application in routine care, substantial challenges remain. While the 1H-NMR assay is robust and cheaper than mass-spectrometry-based alternatives, sensitivity is lower. Also, current metabolite coverage is relatively narrow and lipid focused^13,14,60^. Although a future expansion of metabolite coverage is expected, it presents a limitation for clinical utility to date. Further, downstream quantification from raw NMR spectra needs to be harmonized for the reliable application of multivariable prediction models. While our study population is more healthy and less deprived than the general UK population^37^, the results of external validation in four independent cohorts indicate general transferability of metabolomic states. However, the scope of validation was limited by the available endpoint information, constraining the replication to a subset of seven endpoints. In light of these limitations, we recommend careful scrutinization before application of the metabolomic state model beyond the validated conditions or in specific populations outside the research context. Ultimately, a broad rollout of NMR metabolomics for clinical care requires multiple logistical questions to be addressed, including both sample processing and transport.

Taken together, our work demonstrates the potential and limitations of NMR-derived metabolomic profiles as a multidisease assay to inform on the risk of many common diseases simultaneously.

## Methods

### Data source and endpoint definition

We use data from the UK Biobank cohort, a sample of the UK’s general population. Participants were enrolled from 2006 to 2010 in 22 recruitment centers across the United Kingdom; the follow-up is ongoing. The UK Biobank provides NMR metabolomics measured at recruitment for a subset of individuals: 63,903 women and 54,078 men aged 37–73 years at the time of baseline assessment.

Details on the characteristics of the external validation cohorts are provided in Whitehall II Cohort, Rotterdam Study, Leiden Longevity Study and PROspective Study of Pravastatin in the Elderly at Risk. We investigated a set of 24 endpoints, each defined by the earliest occurrence in primary care, hospital episode statistics or death records. Endpoints were adapted from an earlier study^21^ and defined by ICD10 codes (Supplementary Table 1), and patients with previous disease were excluded for each endpoint. In the case of cardiovascular endpoints we also excluded patients with lipid-lowering therapy records. In addition, we analyzed only men or only women for predominantly sex-specific diseases such as prostate and breast cancer.

The study adhered to the transparent reporting of a multivariable prediction model for individual prognosis or diagnosis (TRIPOD) statement for reporting^61^. The complete checklist can be found in Supplementary Note 1.

### Predictor selection and extraction

We investigated three sets of clinical predictor sets—Age+Sex, ASCVD and PANEL. An overview of the predictors and their use in the respective covariate sets is presented in Fig. 1d. The NMR assay covers 168 metabolites, from multiple amino acids to lipids, lipoproteins, cholesterol subtypes and inflammation markers. While the NMR assay further includes 81 percentage ratios derived from combinations of the 168 original measures, these were not included in the analysis. Basic demographic information was extracted from primary care records and matched with data collected at the study’s recruitment interview. Lifestyle information was extracted from the questionnaire completed at recruitment. Physical measurements and laboratory measures were taken at recruitment. Pre-existing medical conditions were extracted from the questionnaire, interview at recruitment, primary care records and hospital episode statistics. Medications were extracted from the recruitment interview. Cardiovascular predictors were selected based on ESC- and AHA-recommended cardiovascular risk scores for primary prevention, the AHA–ASCVD score^3^ and the ESC–SCORE2 (ref. ^62^). For the PANEL predictor set we included additional predictors from CAIDE^33^ and FINDRISC^32^ scores and comprehensive information on lifestyle, demographics, physical measurements and laboratory values available in the primary care setting. Because genotyping is currently not commonly available in primary care, we decided to omit the *APOE4* status in the primary analysis. A dedicated analysis, including *APOE4* carrier status for all-cause dementia, can be found in Extended Data Fig. 6. A list of all clinical predictors applied in this study is presented in Supplementary Table 2 and a list of all metabolomic predictors in Supplementary Table 3.

### Dataset partitions and imputation

For model development and testing, we split the dataset into 22 spatially separated partitions based on the location of the assessment center at recruitment as previously established^63^. We analyzed the data in 22-fold nested cross-validation, setting aside one of the spatially separated partitions as a test set, aggregating the remaining partitions and randomly selecting 10% of the aggregated data for the validation set. Within each of the 22 cross-validation loops, the individual test set (that is, the spatially separated partition) remained untouched throughout model development and the validation set was used to validate the fitting progress and checkpoint selection. All 22 obtained models were then evaluated on their respective test sets. We assumed that missing data occurred at random and performed multiple imputations using chained equations with random forests^64^. Continuous variables were standardized; Categorical variables were one-hot encoded. Imputation models were fitted on the training sets and applied to the respective validation and test sets.

### Metabolomic state model

The metabolomic state model is a residual neural network simultaneously predicting the metabolomic state for each of the 24 endpoints. The model consists of a shared network and smaller endpoint-specific head networks. The shared neural network comprises three fully connected linear layers, each with batch normalization, dropout^65^ of 0.3 and sigmoid-weighted linear units (SiLU)^66^ activations with 256, 256 and 512 nodes. It outputs a representation of size 512, which is passed on to the endpoint-specific residual head networks. Thereby, each of the 24 residual head networks takes two inputs: the shared representation learned by the shared network and the original 168 metabolomic markers. Each residual head network consists of a small 256-, 128- or 32-node multilayer perceptron (MLP) with a dropout of 0.6, batch normalization and SiLU activations that transform the shared representation, and a skip-connection^67^ network of 128, 128 and 32 nodes transforming the 168 metabolomic markers. The outputs of both networks are subsequently added in a skip-connection and fed through another two-layer, fully connected network of 128 and 128 nodes with a dropout of 0.6, batch normalization and SiLU activations before the scalar metabolomic state is computed through a final single-output linear layer with identity activation. For each endpoint, and thus for each metabolomic state, we individually calculate an adapted proportional hazards loss^68^, excluding prevalent events endpoint specifically. The individual losses are averaged and then summed to derive the final loss of the metabolomic state model. After architecture development, a hyperparameter search is run on training and validation splits of partition zero as random search over a constrained parameter space tuning batch size, initial learning rate, number of nodes in the layers of the endpoint heads and size of the output vector of the shared network. The final models are trained with batch size 1,024 for a maximum of 100 epochs using the Adam optimizer^69^ with default parameters, stochastic weight averaging, a learning rate of 0.001 and early stopping tracking of the performance on each partition’s validation set. We further apply a multistep learning rate schedule with gamma 0.1 and steps at 20, 30 and 40 epochs. We implement the metabolomic state model model in Python v.3.7 using PyTorch v.1.7 (ref. ^70^) and PyTorch-lightning v.1.4.

### Survival analysis and metabolomic state integration

We fitted CPH models^23^ to derive risk predictions for the individual endpoints. Specifically, for each endpoint we developed models on seven distinct covariate sets: first, only the learned metabolomic state; second, the three clinical predictor sets age and sex, cardiovascular predictors and the comprehensive PANEL (Table 1, Fig. 1d and Predictor selection and extraction); and third, clinical predictors with the added metabolomic states for the respective endpoint. Model development was repeated independently for each assessment center and thus, for each cross-validation split, models were trained on the respective training set and checkpoints for the metabolomic state model were selected on the respective validation set. For the final evaluation, predictions made on the respective test sets were aggregated. Harrell’s *C*-index was calculated with the Python package lifelines^71^ by bootstrapping both the aggregated test set and individual assessment centers. Statistical inferences about model differences were based on the distribution of bootstrapped differences in the *C*-index; performances were considered significantly different when the 95% CIs of the performance deltas did not overlap with 0. CPH models were fitted with CoxPHFitter from the Python package lifelines^71^, with default parameters and step size of 0.5 and 0.1 to facilitate model convergence. To estimate risk trajectory based on the metabolomic state, partial metabolomics effects were calculated using a custom adaptation of lifelines CoxPHFitter’s plot_partial_effects_on_outcome method, fixing all other predictors to their central values. CIs for all statistical analyses were calculated with >1,000 bootstrapping iterations. All statistical analyses were performed in R v.4.0.2 (ref. ^72^).

### Feature attribution estimates

SHAP values^41,73^ were calculated to estimate feature attribution for each endpoint and model individually. SHAP values are a combination of game-theoretically optimal Shapley values, which determine the estimated average marginal contribution of each feature for a prediction with local additivity^41,73^. Because computation time of exact SHAP values grows exponentially with an increasing number of features, we resort to an approximation of SHAP values: DeepSHAP, an adaptation of the DeepLIFT^74^ method. Importantly, the sum of the approximated SHAP values amounts to the difference between the expected model prediction on a given set of background samples and the prediction for an observed sample. Calculations were performed using the DeepExplainer method implemented in v.0.39 of the SHAP package^75^. After calculation of per-sample attributions for each metabolite and endpoint, attributions were aggregated per endpoint to derive a global metabolite-specific set of attributions. We identified important attributes based on the top and bottom 1% percentile borders of the SHAP value distribution over all attributions.

### Individual metabolite attribution profiles

Computation of SHAP values (Feature attribution estimates) enabled the derivation of attribution profiles for each individual and disease, informing on the specific contribution of metabolites to individual risk. Individual high-impact metabolites were defined by the top and bottom 1% percentiles of the metabolite SHAP distribution (that is, SHAP ∉ (−0.2,0.2)). To assess the space of individual attribution profiles, UMAP^46^ for dimension reduction was fitted on the entire set of SHAP values for each endpoint individually. The UMAP projection allows assessment of the complex, high-dimensional manifold of attribution values in two-dimensional space. UMAPs were fitted using the UMAP Python package^76^ and default parameters. For visualization of UMAP space, 41 unconnected outliers of 117,981 total observations were excluded.

### Replication in independent cohorts

The models fitted in the UK Biobank were exported via ONNX^77^, and calculation of metabolomic states was replicated in the Whitehall II Cohort^24^, the Rotterdam Study^25^, the Leiden Longevity Study^26^ and the PROspective Study of Pravastatin in the Elderly at Risk^27,78^ (Fig. 1c and Supplementary Table 4). In consideration of available predictors and endpoints, CPH models were fitted and evaluated as described in Survival analysis and metabolomic state integration. The ONNX weights of the model, as well as the normalization pipeline for the NMR data as fitted on the UK Biobank, are available through our GitHub repository (Code availability).

### Whitehall II Cohort

The Whitehall II Cohort (WHII) is an ongoing prospective cohort study of adults, consisting of 10,308 individuals (3,413 women and 6,895 men) recruited at age 35–55 years^24^. At the time of recruitment (1985–1988), all study participants were working in the London offices of 20 Whitehall departments. Participants have been followed up regularly over the years, with questionnaires and self-examination conducted every 5 years. NMR profiling was performed from serum samples between 1997 and 1999.

### Rotterdam Study

The Rotterdam Study (RS) is a prospective, population-based cohort study^25^ with the aim of determining the occurrence of common diseases in elderly people. Baseline examination took place in 1990, with approximately 7,983 persons aged 55 years and older undergoing a home interview and extensive physical examination. Follow-up visits took place every 3–4 years (RS-I)^25^. The study was later extended to two stages and contained 14,926 subjects as of 2008. Written informed consent was obtained from all participants, and the Medical Ethics Committee of the Erasmus Medical Center, Rotterdam, approved the study^25^. Metabolomics measurements were quantified in fasted EDTA plasma samples using the Nightingale Health platform. We included all 2,949 samples with complete baseline covariates and NMR metabolomics that were available in the BBMRI-NL platform.

### Leiden Longevity Study

The Leiden Longevity Study (LLS) consists of 421 long-lived families of European descent. Families were included if at least two long-lived siblings were alive and fulfilled the age criterion of 89 years or older for males and 91 years or older for females, representing <0.5% of the Dutch population in 2001 (ref. ^26^). In total, 944 long-lived proband siblings (mean age 94 years, range 89–104), 1,671 offspring (mean age 61 years, range 39–81) and 744 spouses thereof (mean age 60 years, range 36–79) were included. Registry-based follow-up until 27 October 2016 was available for all participants. Metabolites were successfully quantified in 843 nonagenarians, 1,157 of their offspring and 684 controls using nonfasted EDTA plasma samples. We included all 1,655 samples of the offspring and spouse population with complete baseline covariates and NMR metabolomics available in the BBMRI-NL platform.

### PROspective Study of Pravastatin in the Elderly at Risk

The PROspective Study of Pravastatin in the Elderly at Risk (PROSPER) trial is a double-blind, randomized, placebo-controlled trial investigating the benefit of pravastatin (40 mg d^–1^) in elderly individuals at risk of CVD^27,78^. In total, 5,804 participants (70–82 years) were identified in the primary care setting between December 1997 and May 1999 from three centers: Glasgow (UK) Cork (Ireland) and Leiden (the Netherlands). The mean follow-up period was 3.2 years. All included patients either had evidence of vascular disease (physician-diagnosed stable angina, stroke, transient ischemic attack or myocardial infarction) or high risk of vascular disease as determined by hypertension, diabetes or smoking status. Fasting venous blood samples were collected at baseline and at 3-month intervals and stored at −80 °C. For the present study, all individuals recruited at the Leiden recruitment center and with NMR metabolomics data available through the BBMRI-NL consortium (in total, 960 individuals) were included, employing the study as a cohort study. NMR metabolomics was quantified from previously unthawed 6-month postrandomization samples.

### Reporting summary

Further information on research design is available in the Nature Research Reporting Summary linked to this article.

## Online content

Any methods, additional references, Nature Research reporting summaries, source data, extended data, supplementary information, acknowledgements, peer review information; details of author contributions and competing interests; and statements of data and code availability are available at 10.1038/s41591-022-01980-3.

## Supplementary information

Supplementary Information Supplementary Note 1 (TRIPOD checklist) and Figs. 1 and 2.

Reporting Summary

Supplementary Tables Supplementary Tables 1–12

## Data Availability

UK Biobank data, including NMR metabolomics, are publicly available to bona fide researchers upon application at http://www.ukbiobank.ac.uk/using-the-resource/. Detailed information on predictors and endpoints used in this study is presented in Supplementary Tables 1–3. WHII data are available for the scientific community, and researchers are invited to apply for data access at https://www.dementiasplatform.uk/. Data from the BBMRI-NL consortium are available upon application at https://www.bbmri.nl/Omics-metabolomics.

## Code availability

All code developed and used throughout this study has been made open source and is available on GitHub. The code used to train the metabolomic state model can be found at github.com/thbuerg/MetabolomicsCommonDiseases, while the code used to run analysis on trained models can be found at github.com/JakobSteinfeldt/MetabolomicsCommonDiseases.
