# Supplementary material for: Metabolomic profiles predict individual multidisease outcomes
Source: Nat Med. 2022 Sep 22;28(11):2309–20. doi: 10.1038/s41591-022-01980-3 (PMC9671812; doi:10.1038/s41591-022-01980-3)
Supplement: Supplementary file 1 — Supplementary Note 1 (TRIPOD checklist) and Figs. 1 and 2. [file 41591_2022_1980_MOESM1_ESM.pdf]

nature portfolio

https://doi.org/10.1038/s41591-022-01980-3

Supplementary information

Metabolomic profiles predict individual multidisease outcomes

In the format provided by the authors and unedited

Supplementary Note 1

TRIPOD Checklist: Prediction Model Development and Validation

Section/Topic	Item		Checklist Item	Page
**Title and abstract**				
Title	1	D;V	Identify the study as developing and/or validating a multivariable prediction model, the target population, and the outcome to be predicted.	1
Abstract	2	D;V	Provide a summary of objectives, study design, setting, participants, sample size, predictors, outcome, statistical analysis, results, and conclusions.	4
**Introduction**				
Background and objectives	3a	D;V	Explain the medical context (including whether diagnostic or prognostic) and rationale for developing or validating the multivariable prediction model, including references to existing models.	5ff
	3b	D;V	Specify the objectives, including whether the study describes the development or validation of the model or both.	5ff
**Methods**				
Source of data	4a	D;V	Describe the study design or source of data (e.g., randomized trial, cohort, or registry data), separately for the development and validation data sets, if applicable.	5ff, 37ff
	4b	D;V	Specify the key study dates, including start of accrual; end of accrual; and, if applicable, end of follow-up.	37ff
Participants	5a	D;V	Specify key elements of the study setting (e.g., primary care, secondary care, general population) including number and location of centres.	6+7, 37ff
	5b	D;V	Describe eligibility criteria for participants.	6+7, 37ff
	5c	D;V	Give details of treatments received, if relevant.	37ff
Outcome	6a	D;V	Clearly define the outcome that is predicted by the prediction model, including how and when assessed.	37ff, Supplementary Table 1
	6b	D;V	Report any actions to blind assessment of the outcome to be predicted.	NA
Predictors	7a	D;V	Clearly define all predictors used in developing or validating the multivariable prediction model, including how and when they were measured.	Supplementary Table 1,2,3
	7b	D;V	Report any actions to blind assessment of predictors for the outcome and other predictors.	NA
Sample size	8	D;V	Explain how the study size was arrived at.	NA
Missing data	9	D;V	Describe how missing data were handled (e.g., complete-case analysis, single imputation, multiple imputation) with details of any imputation method.	37+38
Statistical analysis methods	10a	D	Describe how predictors were handled in the analyses.	38, 39, 40
	10b	D	Specify type of model, all model-building procedures (including any predictor selection), and method for internal validation.	5-8, 38ff
	10c	V	For validation, describe how the predictions were calculated.	41+42
	10d	D;V	Specify all measures used to assess model performance and, if relevant, to compare multiple models.	9,10,11, 40+41
	10e	V	Describe any model updating (e.g., recalibration) arising from the validation, if done.	41
Risk groups	11	D;V	Provide details on how risk groups were created, if done.	12+13
Development vs. validation	12	V	For validation, identify any differences from the development data in setting, eligibility criteria, outcome, and predictors.	41
**Results**				
Participants	13a	D;V	Describe the flow of participants through the study, including the number of participants with and without the outcome and, if applicable, a summary of the follow-up time. A diagram may be helpful.	6,7,8, Fig1
	13b	D;V	Describe the characteristics of the participants (basic demographics, clinical features, available predictors), including the number of participants with missing data for predictors and outcome.	6,7,8, Table 1
	13c	V	For validation, show a comparison with the development data of the distribution of important variables (demographics, predictors and outcome).	Suppl. Table 4
Model development	14a	D	Specify the number of participants and outcome events in each analysis.	Suppl. Table 1
	14b	D	If done, report the unadjusted association between each candidate predictor and outcome.	NA
Model specification	15a	D	Present the full prediction model to allow predictions for individuals (i.e., all regression coefficients, and model intercept or baseline survival at a given time point).	39, Github
	15b	D	Explain how to the use the prediction model.	39, Github
Model performance	16	D;V	Report performance measures (with CIs) for the prediction model.	Fig. 3,4 Extended Data Fig. 4
Model-updating	17	V	If done, report the results from any model updating (i.e., model specification, model performance).	NA
**Discussion**				
Limitations	18	D;V	Discuss any limitations of the study (such as nonrepresentative sample, few events per predictor, missing data).	17ff
Interpretation	19a	V	For validation, discuss the results with reference to performance in the development data, and any other validation data.	18, 17
	19b	D;V	Give an overall interpretation of the results, considering objectives, limitations, results from similar studies, and other relevant evidence.	17ff
Implications	20	D;V	Discuss the potential clinical use of the model and implications for future research.	17ff
**Other information**				
Supplementary information	21	D;V	Provide information about the availability of supplementary resources, such as study protocol, Web calculator, and data sets.	20
Funding	22	D;V	Give the source of funding and the role of the funders for the present study.	20ff

*Items relevant only to the development of a prediction model are denoted by D, items relating solely to a validation of a prediction model are denoted by V, and items relating to both are denoted D;V. We recommend using the TRIPOD Checklist in conjunction with the TRIPOD Explanation and Elaboration document.

Supplementary Figure 1

Supplementary Figure 1: Model calibration and clinical utility for all endpoints

a-x) Displayed are calibration curves and clinical utility decision curves for all endpoints. In each panel, the left figure displays the calibration curve for Cox proportional hazard models, including the baseline parameter sets AgeSex, ASCVD, and PANEL, as well as their extensions with the metabolomic state (i.e. AgeSex+MET). All models are well-calibrated. The center figure displays endpoint-specific net benefit curves standardized by the endpoint prevalence, where the horizontal solid gray line indicates “treat none” and the vertical solid gray line indicates “treat all”. The standardized net benefit of AgeSex (gray line), ASCVD (dashed dark gray line) and the PANEL set (dotted black line), are compared to AgeSex+MET (green line) and additional non-laboratory predictors of the PANEL (PANELnoLaboratory, blue line). The green alpha indicates the added benefit of the metabolomic state addition over AgeSex, while the blue alpha indicates added performance over PANELnoLaboratory, respectively. The right figure displays the standardized net benefit curves comparing the performance of PANEL+MET (solid black line) against all baselines: AgeSex (gray line), ASCVD (dashed dark gray line), and the PANEL set (dotted black line).

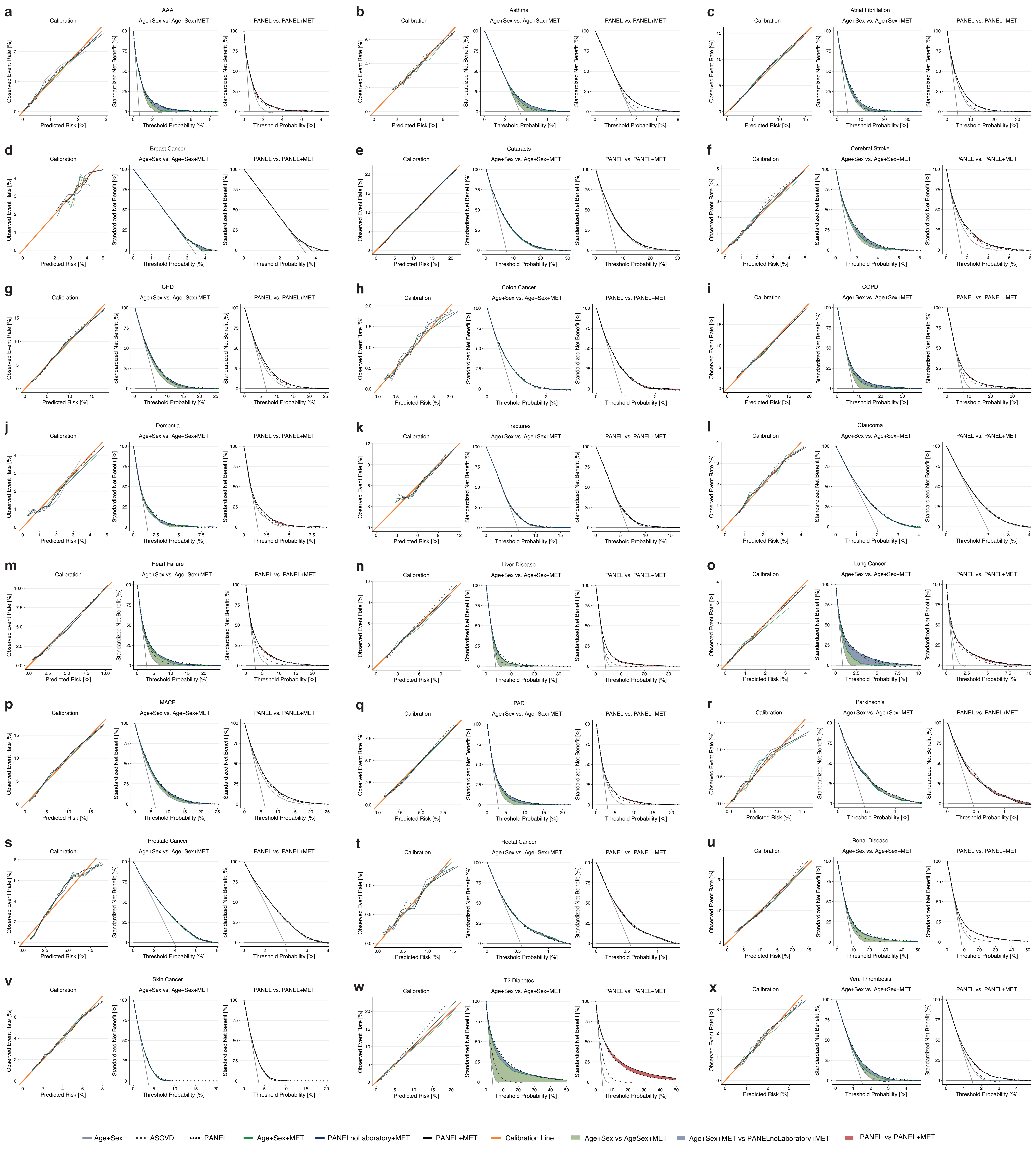
a AAA
b Asthma
c Atrial Fibrillation
d Breast Cancer
e Cataracts
f Cerebral Stroke
g CHD
h Colon Cancer
i COPD
j Dementia
k Fractures
l Glaucoma
m Heart Failure
n Liver Disease
o Lung Cancer
p MACE
q PAD
r Parkinson's
s Prostate Cancer
t Rectal Cancer
u Renal Disease
v Skin Cancer
w T2 Diabetes
x Ven. Thrombosis
Calibration
Age+Sex vs. Age+Sex+MET
PANEL vs. PANEL+MET
Observed Event Rate [%]
Predicted Risk [%]
Standardized Net Benefit [%]
Threshold Probability [%]
Age+Sex
ASCVD
PANEL
Age+Sex+MET
PANELnoLaboratory+MET
PANEL+MET
Calibration Line
Age+Sex vs AgeSex+MET
Age+Sex+MET vs PANELnoLaboratory+MET
PANEL vs PANEL+MET

Supplementary Figure 2

Supplementary Figure 2: Global attributions for all endpoints

a-x) Global metabolite attributions for all endpoints. Individual attributions are aggregated by percentiles. Each dot indicates one percentile. The more distant a dot from the circular baseline, the stronger the absolute attribution for the percentile. Deviations toward the center represent negative, and deviations toward the outside represent positive contributions to the metabolomic state. The color indicates the metabolite's mean normalized plasma value.

a

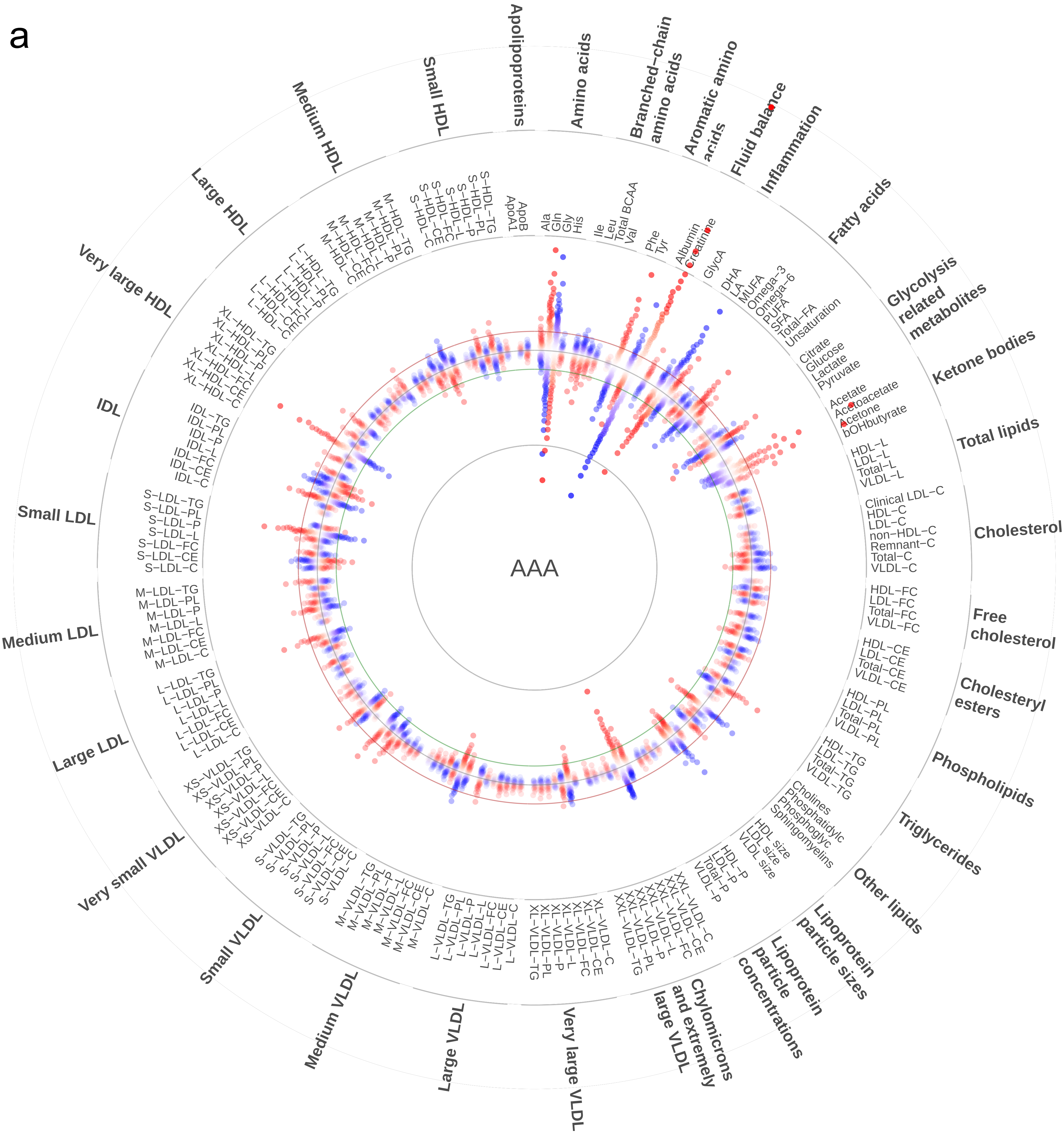
AAA
Apolipoproteins
Amino acids
Branched−chain amino acids
Aromatic amino acids
Fluid balance
Inflammation
Fatty acids
Glycolysis related metabolites
Ketone bodies
Total lipids
Cholesterol
Free cholesterol
Cholesteryl esters
Phospholipids
Triglycerides
Other lipids
Lipoprotein particle sizes
Lipoprotein particle concentrations
Chylomicrons and extremely large VLDL
Very large VLDL
Large VLDL
Medium VLDL
Small VLDL
Very small VLDL
Large LDL
Medium LDL
Small LDL
IDL
Very large HDL
Large HDL
Medium HDL
Small HDL
ApoB
ApoA1
Ala
Gln
Gly
His
Ile
Leu
Total BCAA
Val
Phe
Tyr
Albumin
Creatinine
GlycA
DHA
LA
MUFA
Omega−3
Omega−6
PUFA
SFA
Total−FA
Unsaturation
Citrate
Glucose
Lactate
Pyruvate
Acetate
Acetoacetate
Acetone
bOHbutyrate
HDL−L
LDL−L
Total−L
VLDL−L
Clinical LDL−C
HDL−C
LDL−C
non−HDL−C
Remnant−C
Total−C
VLDL−C
HDL−FC
LDL−FC
Total−FC
VLDL−FC
HDL−CE
LDL−CE
Total−CE
VLDL−CE
HDL−PL
LDL−PL
Total−PL
VLDL−PL
HDL−TG
LDL−TG
Total−TG
VLDL−TG
Cholines
Phosphatidylc
Phosphoglyc
Sphingomyelins
HDL size
LDL size
VLDL size
HDL−P
LDL−P
Total−P
VLDL−P
XXL−VLDL−C
XXL−VLDL−CE
XXL−VLDL−FC
XXL−VLDL−L
XXL−VLDL−P
XXL−VLDL−PL
XXL−VLDL−TG
XL−VLDL−C
XL−VLDL−CE
XL−VLDL−FC
XL−VLDL−L
XL−VLDL−P
XL−VLDL−PL
XL−VLDL−TG
L−VLDL−TG
L−VLDL−PL
L−VLDL−P
L−VLDL−L
L−VLDL−FC
L−VLDL−CE
L−VLDL−C
M−VLDL−TG
M−VLDL−PL
M−VLDL−P
M−VLDL−L
M−VLDL−FC
M−VLDL−CE
M−VLDL−C
S−VLDL−TG
S−VLDL−PL
S−VLDL−P
S−VLDL−L
S−VLDL−FC
S−VLDL−CE
S−VLDL−C
XS−VLDL−TG
XS−VLDL−PL
XS−VLDL−P
XS−VLDL−L
XS−VLDL−FC
XS−VLDL−CE
XS−VLDL−C
L−LDL−TG
L−LDL−PL
L−LDL−P
L−LDL−L
L−LDL−FC
L−LDL−CE
L−LDL−C
M−LDL−TG
M−LDL−PL
M−LDL−P
M−LDL−L
M−LDL−FC
M−LDL−CE
M−LDL−C
S−LDL−TG
S−LDL−PL
S−LDL−P
S−LDL−L
S−LDL−FC
S−LDL−CE
S−LDL−C
IDL−TG
IDL−PL
IDL−P
IDL−L
IDL−FC
IDL−CE
IDL−C
XL−HDL−TG
XL−HDL−PL
XL−HDL−P
XL−HDL−L
XL−HDL−FC
XL−HDL−CE
XL−HDL−C
L−HDL−TG
L−HDL−PL
L−HDL−P
L−HDL−L
L−HDL−FC
L−HDL−CE
L−HDL−C
M−HDL−TG
M−HDL−PL
M−HDL−P
M−HDL−L
M−HDL−FC
M−HDL−CE
M−HDL−C
S−HDL−TG
S−HDL−PL
S−HDL−P
S−HDL−L
S−HDL−FC
S−HDL−CE
S−HDL−C

b

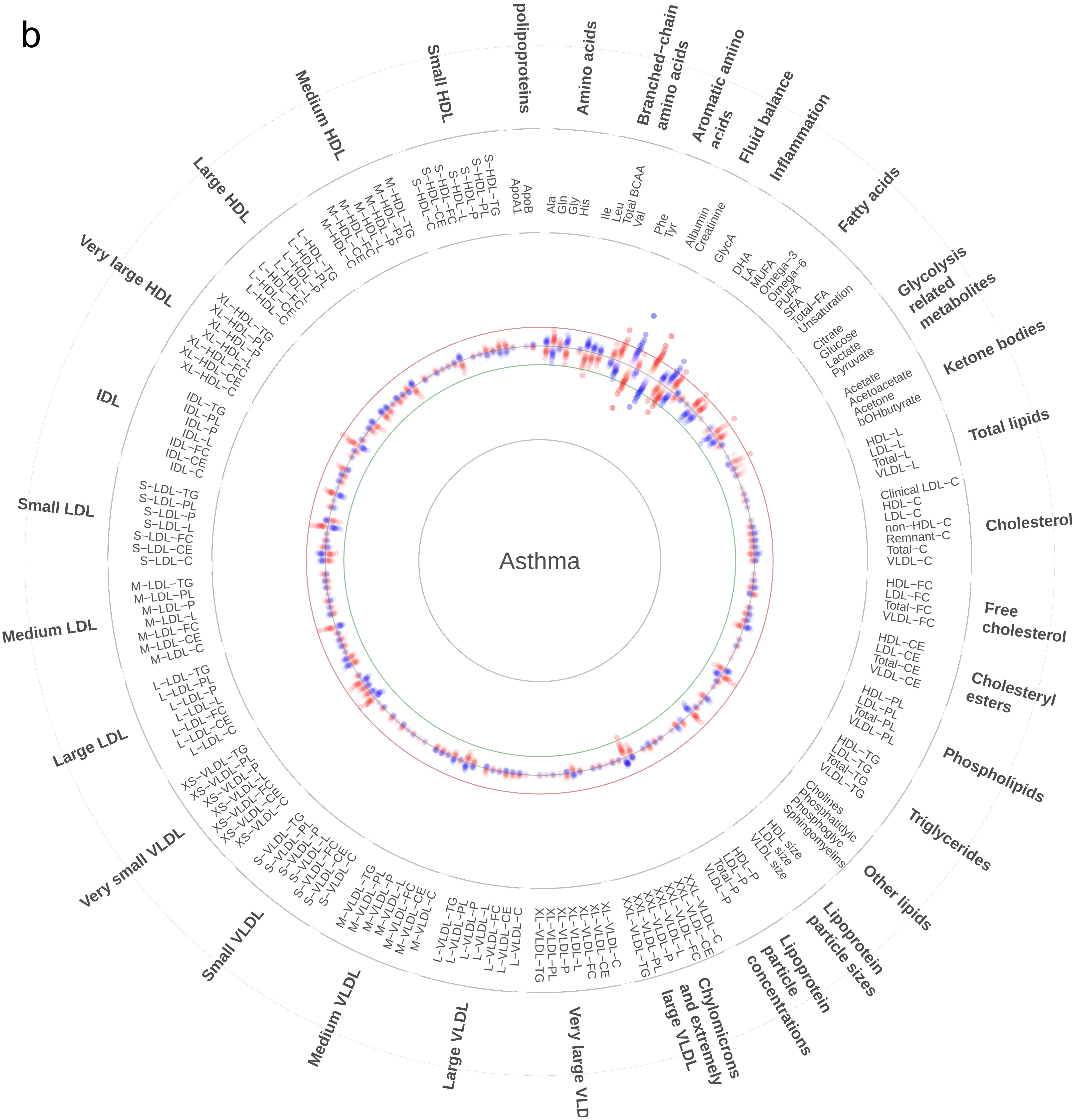

Asthma
polipoproteins
ApoB
ApoA1
Amino acids
Ala
Gln
Gly
His
Branched−chain amino acids
Ile
Leu
Total BCAA
Val
Aromatic amino acids
Phe
Tyr
Fluid balance
Albumin
Creatinine
Inflammation
GlycA
Fatty acids
DHA
LA
MUFA
Omega−3
Omega−6
PUFA
SFA
Total−FA
Unsaturation
Glycolysis related metabolites
Citrate
Glucose
Lactate
Pyruvate
Ketone bodies
Acetate
Acetoacetate
Acetone
bOHbutyrate
Total lipids
HDL−L
LDL−L
Total−L
VLDL−L
Cholesterol
Clinical LDL−C
HDL−C
LDL−C
non−HDL−C
Remnant−C
Total−C
VLDL−C
Free cholesterol
HDL−FC
LDL−FC
Total−FC
VLDL−FC
Cholesteryl esters
HDL−CE
LDL−CE
Total−CE
VLDL−CE
Phospholipids
HDL−PL
LDL−PL
Total−PL
VLDL−PL
Triglycerides
HDL−TG
LDL−TG
Total−TG
VLDL−TG
Other lipids
Cholines
Phosphatidylc
Phosphoglyc
Sphingomyelins
Lipoprotein particle sizes
HDL size
LDL size
VLDL size
Lipoprotein particle concentrations
HDL−P
LDL−P
Total−P
VLDL−P
Chylomicrons and extremely large VLDL
XXL−VLDL−C
XXL−VLDL−CE
XXL−VLDL−FC
XXL−VLDL−L
XXL−VLDL−P
XXL−VLDL−PL
XXL−VLDL−TG
Very large VLD
XL−VLDL−C
XL−VLDL−CE
XL−VLDL−FC
XL−VLDL−L
XL−VLDL−P
XL−VLDL−PL
XL−VLDL−TG
Large VLDL
L−VLDL−C
L−VLDL−CE
L−VLDL−FC
L−VLDL−L
L−VLDL−P
L−VLDL−PL
L−VLDL−TG
Medium VLDL
M−VLDL−C
M−VLDL−CE
M−VLDL−FC
M−VLDL−L
M−VLDL−P
M−VLDL−PL
M−VLDL−TG
Small VLDL
S−VLDL−C
S−VLDL−CE
S−VLDL−FC
S−VLDL−L
S−VLDL−P
S−VLDL−PL
S−VLDL−TG
Very small VLDL
XS−VLDL−C
XS−VLDL−CE
XS−VLDL−FC
XS−VLDL−L
XS−VLDL−P
XS−VLDL−PL
XS−VLDL−TG
Large LDL
L−LDL−TG
L−LDL−PL
L−LDL−P
L−LDL−L
L−LDL−FC
L−LDL−CE
L−LDL−C
Medium LDL
M−LDL−TG
M−LDL−PL
M−LDL−P
M−LDL−L
M−LDL−FC
M−LDL−CE
M−LDL−C
Small LDL
S−LDL−TG
S−LDL−PL
S−LDL−P
S−LDL−L
S−LDL−FC
S−LDL−CE
S−LDL−C
IDL
IDL−TG
IDL−PL
IDL−P
IDL−L
IDL−FC
IDL−CE
IDL−C
Very large HDL
XL−HDL−TG
XL−HDL−PL
XL−HDL−P
XL−HDL−L
XL−HDL−FC
XL−HDL−CE
XL−HDL−C
Large HDL
L−HDL−TG
L−HDL−PL
L−HDL−P
L−HDL−L
L−HDL−FC
L−HDL−CE
L−HDL−C
Medium HDL
M−HDL−TG
M−HDL−PL
M−HDL−P
M−HDL−L
M−HDL−FC
M−HDL−CE
M−HDL−C
Small HDL
S−HDL−TG
S−HDL−PL
S−HDL−P
S−HDL−L
S−HDL−FC
S−HDL−CE
S−HDL−C

c

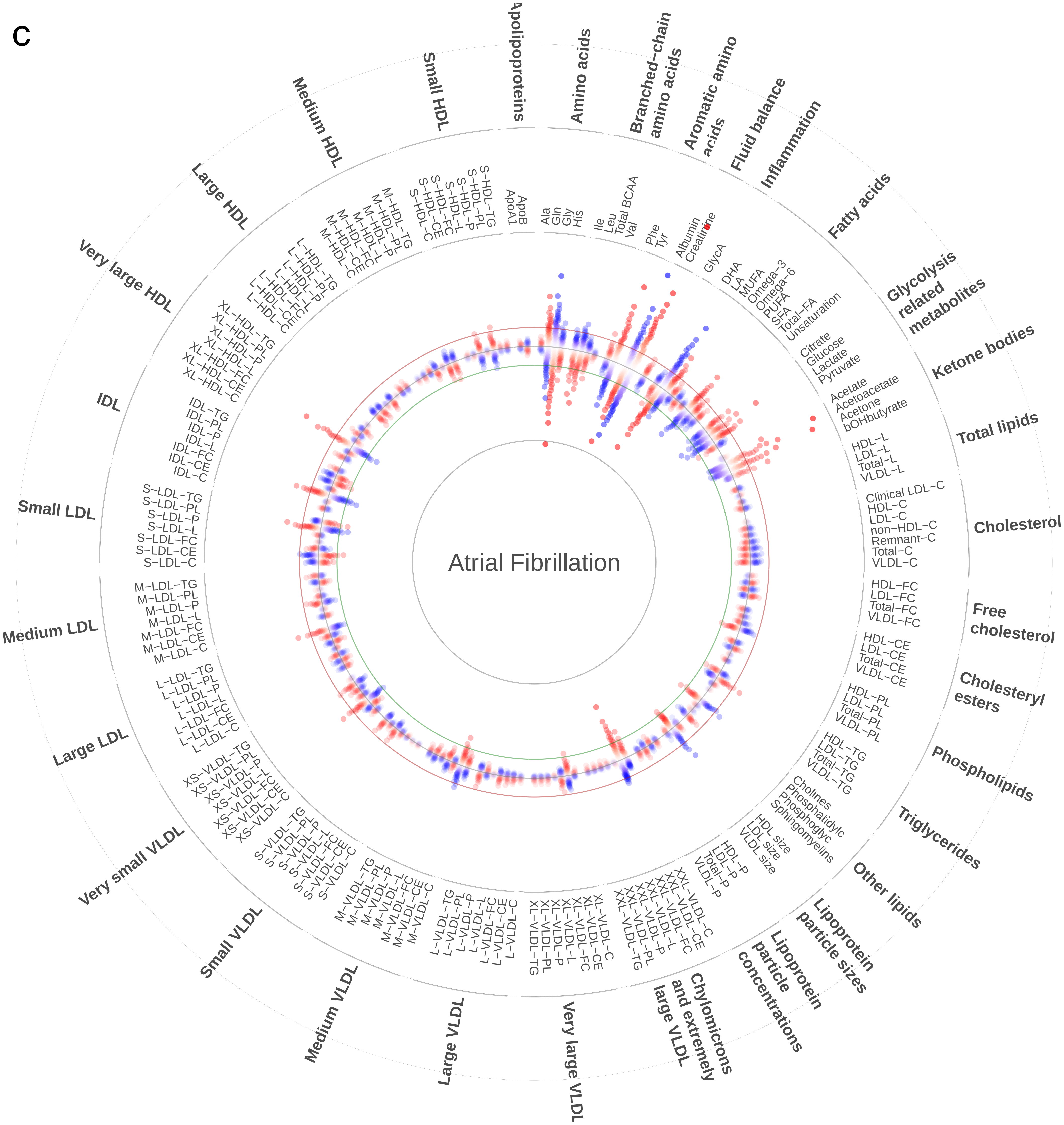

Atrial Fibrillation
Apolipoproteins
Amino acids
Branched−chain amino acids
Aromatic amino acids
Fluid balance
Inflammation
Fatty acids
Glycolysis related metabolites
Ketone bodies
Total lipids
Cholesterol
Free cholesterol
Cholesteryl esters
Phospholipids
Triglycerides
Other lipids
Lipoprotein particle sizes
Lipoprotein particle concentrations
Chylomicrons and extremely large VLDL
Very large VLDL
Large VLDL
Medium VLDL
Small VLDL
Very small VLDL
Large LDL
Medium LDL
Small LDL
IDL
Very large HDL
Large HDL
Medium HDL
Small HDL
ApoB
ApoA1
Ala
Gln
Gly
His
Ile
Leu
Total BCAA
Val
Phe
Tyr
Albumin
Creatinine
GlycA
DHA
LA
MUFA
Omega−3
Omega−6
PUFA
SFA
Total−FA
Unsaturation
Citrate
Glucose
Lactate
Pyruvate
Acetate
Acetoacetate
Acetone
bOHbutyrate
HDL−L
LDL−L
Total−L
VLDL−L
Clinical LDL−C
HDL−C
LDL−C
non−HDL−C
Remnant−C
Total−C
VLDL−C
HDL−FC
LDL−FC
Total−FC
VLDL−FC
HDL−CE
LDL−CE
Total−CE
VLDL−CE
HDL−PL
LDL−PL
Total−PL
VLDL−PL
HDL−TG
LDL−TG
Total−TG
VLDL−TG
Cholines
Phosphatidylc
Phosphoglyc
Sphingomyelins
HDL size
LDL size
VLDL size
HDL−P
LDL−P
Total−P
VLDL−P
XXL−VLDL−C
XXL−VLDL−CE
XXL−VLDL−FC
XXL−VLDL−L
XXL−VLDL−P
XXL−VLDL−PL
XXL−VLDL−TG
XL−VLDL−C
XL−VLDL−CE
XL−VLDL−FC
XL−VLDL−L
XL−VLDL−P
XL−VLDL−PL
XL−VLDL−TG
L−VLDL−TG
L−VLDL−PL
L−VLDL−P
L−VLDL−L
L−VLDL−FC
L−VLDL−CE
L−VLDL−C
M−VLDL−TG
M−VLDL−PL
M−VLDL−P
M−VLDL−L
M−VLDL−FC
M−VLDL−CE
M−VLDL−C
S−VLDL−TG
S−VLDL−PL
S−VLDL−P
S−VLDL−L
S−VLDL−FC
S−VLDL−CE
S−VLDL−C
XS−VLDL−TG
XS−VLDL−PL
XS−VLDL−P
XS−VLDL−L
XS−VLDL−FC
XS−VLDL−CE
XS−VLDL−C
L−LDL−TG
L−LDL−PL
L−LDL−P
L−LDL−L
L−LDL−FC
L−LDL−CE
L−LDL−C
M−LDL−TG
M−LDL−PL
M−LDL−P
M−LDL−L
M−LDL−FC
M−LDL−CE
M−LDL−C
S−LDL−TG
S−LDL−PL
S−LDL−P
S−LDL−L
S−LDL−FC
S−LDL−CE
S−LDL−C
IDL−TG
IDL−PL
IDL−P
IDL−L
IDL−FC
IDL−CE
IDL−C
XL−HDL−TG
XL−HDL−PL
XL−HDL−P
XL−HDL−L
XL−HDL−FC
XL−HDL−CE
XL−HDL−C
L−HDL−TG
L−HDL−PL
L−HDL−P
L−HDL−L
L−HDL−FC
L−HDL−CE
L−HDL−C
M−HDL−TG
M−HDL−PL
M−HDL−P
M−HDL−L
M−HDL−FC
M−HDL−CE
M−HDL−C
S−HDL−TG
S−HDL−PL
S−HDL−P
S−HDL−L
S−HDL−FC
S−HDL−CE
S−HDL−C

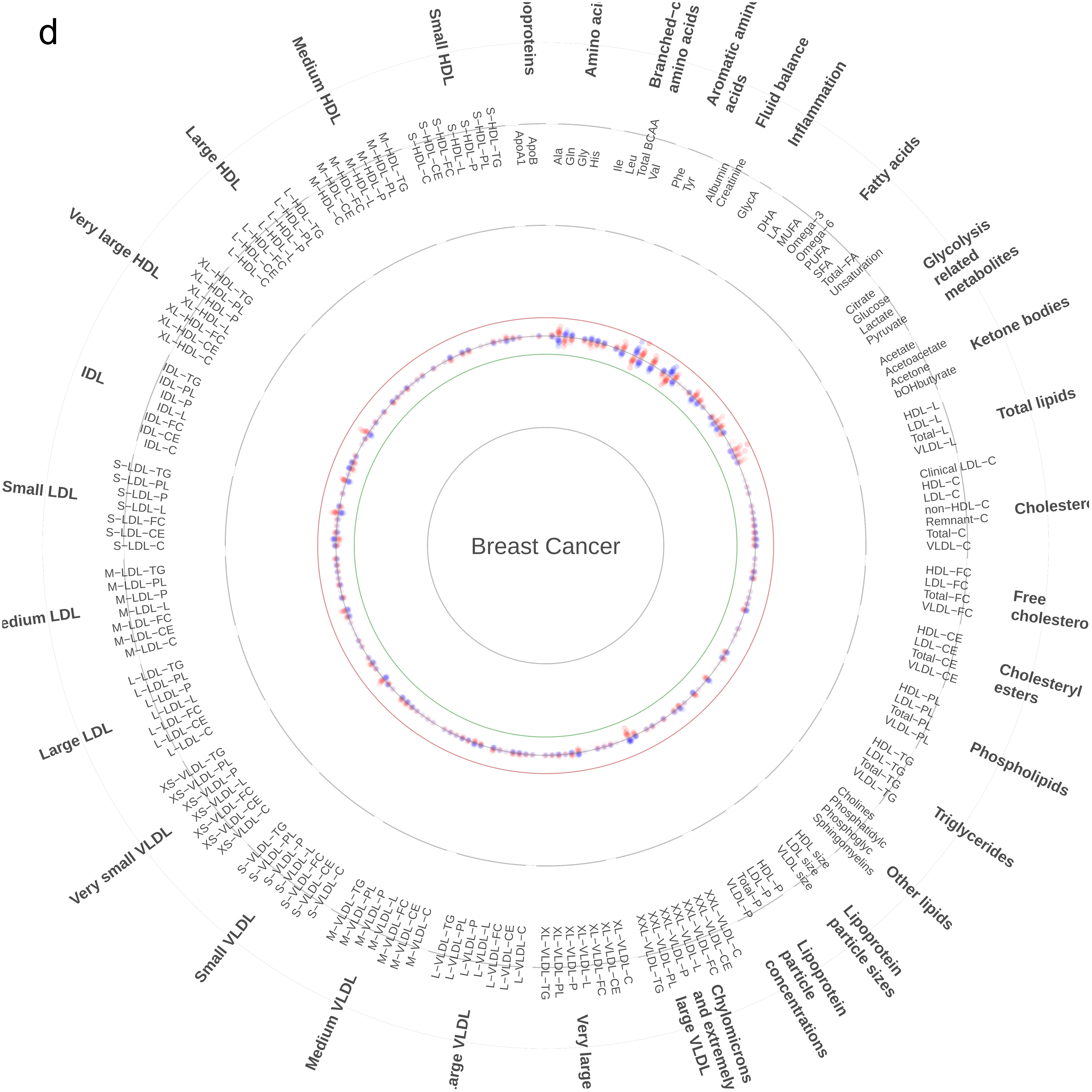

d
Breast Cancer
Small HDL
oproteins
Amino aci
Branched−c amino acids
Aromatic amin acids
Fluid balance
Inflammation
Fatty acids
Glycolysis related metabolites
Ketone bodies
Total lipids
Cholestero
Free cholestero
Cholesteryl esters
Phospholipids
Triglycerides
Other lipids
Lipoprotein particle sizes
Lipoprotein particle concentrations
Chylomicrons and extremely large VLDL
Very large
arge VLDL
Medium VLDL
Small VLDL
Very small VLDL
Large LDL
edium LDL
Small LDL
IDL
Very large HDL
Large HDL
Medium HDL
S−HDL−TG S−HDL−PL S−HDL−P S−HDL−L S−HDL−FC S−HDL−CE S−HDL−C
ApoB ApoA1
Ala Gln Gly His
Ile Leu Total BCAA Val
Phe Tyr
Albumin Creatinine
GlycA
DHA LA MUFA Omega−3 Omega−6 PUFA SFA Total−FA Unsaturation
Citrate Glucose Lactate Pyruvate
Acetate Acetoacetate Acetone bOHbutyrate
HDL−L LDL−L Total−L VLDL−L
Clinical LDL−C HDL−C LDL−C non−HDL−C Remnant−C Total−C VLDL−C
HDL−FC LDL−FC Total−FC VLDL−FC
HDL−CE LDL−CE Total−CE VLDL−CE
HDL−PL LDL−PL Total−PL VLDL−PL
HDL−TG LDL−TG Total−TG VLDL−TG
Cholines Phosphatidylc Phosphoglyc Sphingomyelins
HDL size LDL size VLDL size
HDL−P LDL−P Total−P VLDL−P
XXL−VLDL−C XXL−VLDL−CE XXL−VLDL−FC XXL−VLDL−L XXL−VLDL−P XXL−VLDL−PL XXL−VLDL−TG
XL−VLDL−C XL−VLDL−CE XL−VLDL−FC XL−VLDL−L XL−VLDL−P XL−VLDL−PL XL−VLDL−TG
L−VLDL−TG L−VLDL−PL L−VLDL−P L−VLDL−L L−VLDL−FC L−VLDL−CE L−VLDL−C
M−VLDL−TG M−VLDL−PL M−VLDL−P M−VLDL−L M−VLDL−FC M−VLDL−CE M−VLDL−C
S−VLDL−TG S−VLDL−PL S−VLDL−P S−VLDL−L S−VLDL−FC S−VLDL−CE S−VLDL−C
XS−VLDL−TG XS−VLDL−PL XS−VLDL−P XS−VLDL−L XS−VLDL−FC XS−VLDL−CE XS−VLDL−C
L−LDL−TG L−LDL−PL L−LDL−P L−LDL−L L−LDL−FC L−LDL−CE L−LDL−C
M−LDL−TG M−LDL−PL M−LDL−P M−LDL−L M−LDL−FC M−LDL−CE M−LDL−C
S−LDL−TG S−LDL−PL S−LDL−P S−LDL−L S−LDL−FC S−LDL−CE S−LDL−C
IDL−TG IDL−PL IDL−P IDL−L IDL−FC IDL−CE IDL−C
XL−HDL−TG XL−HDL−PL XL−HDL−P XL−HDL−L XL−HDL−FC XL−HDL−CE XL−HDL−C
L−HDL−TG L−HDL−PL L−HDL−P L−HDL−L L−HDL−FC L−HDL−CE L−HDL−C
M−HDL−TG M−HDL−PL M−HDL−P M−HDL−L M−HDL−FC M−HDL−CE M−HDL−C

e

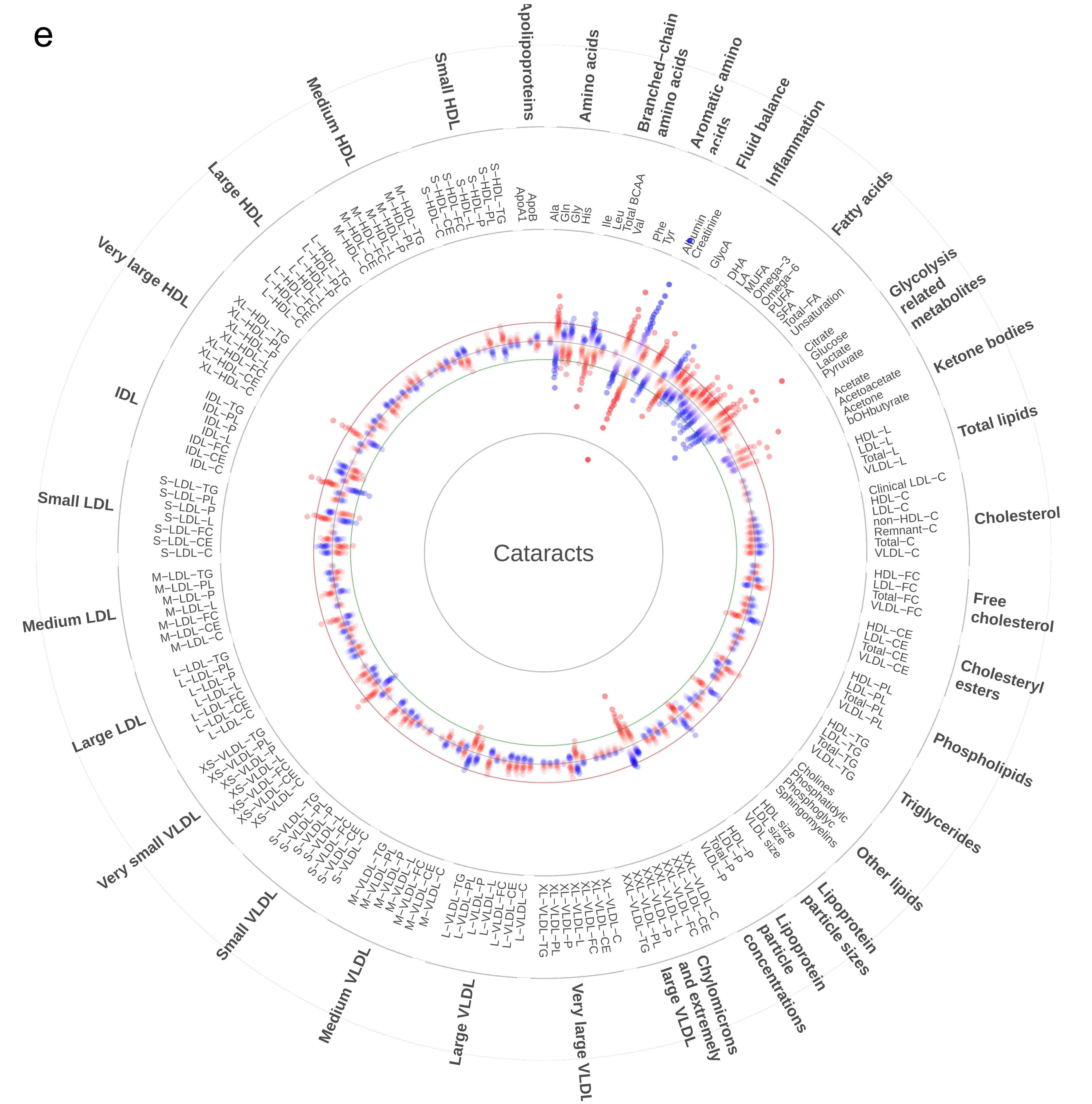

Cataracts
Apolipoproteins
Amino acids
Branched−chain amino acids
Aromatic amino acids
Fluid balance
Inflammation
Fatty acids
Glycolysis related metabolites
Ketone bodies
Total lipids
Cholesterol
Free cholesterol
Cholesteryl esters
Phospholipids
Triglycerides
Other lipids
Lipoprotein particle sizes
Lipoprotein particle concentrations
Chylomicrons and extremely large VLDL
Very large VLDL
Large VLDL
Medium VLDL
Small VLDL
Very small VLDL
Large LDL
Medium LDL
Small LDL
IDL
Very large HDL
Large HDL
Medium HDL
Small HDL
ApoB
ApoA1
Ala
Gln
Gly
His
Ile
Leu
Total BCAA
Val
Phe
Tyr
Albumin
Creatinine
GlycA
DHA
LA
MUFA
Omega−3
Omega−6
PUFA
SFA
Total−FA
Unsaturation
Citrate
Glucose
Lactate
Pyruvate
Acetate
Acetoacetate
Acetone
bOHbutyrate
HDL−L
LDL−L
Total−L
VLDL−L
Clinical LDL−C
HDL−C
LDL−C
non−HDL−C
Remnant−C
Total−C
VLDL−C
HDL−FC
LDL−FC
Total−FC
VLDL−FC
HDL−CE
LDL−CE
Total−CE
VLDL−CE
HDL−PL
LDL−PL
Total−PL
VLDL−PL
HDL−TG
LDL−TG
Total−TG
VLDL−TG
Cholines
Phosphatidylc
Phosphoglyc
Sphingomyelins
HDL size
LDL size
VLDL size
HDL−P
LDL−P
Total−P
VLDL−P

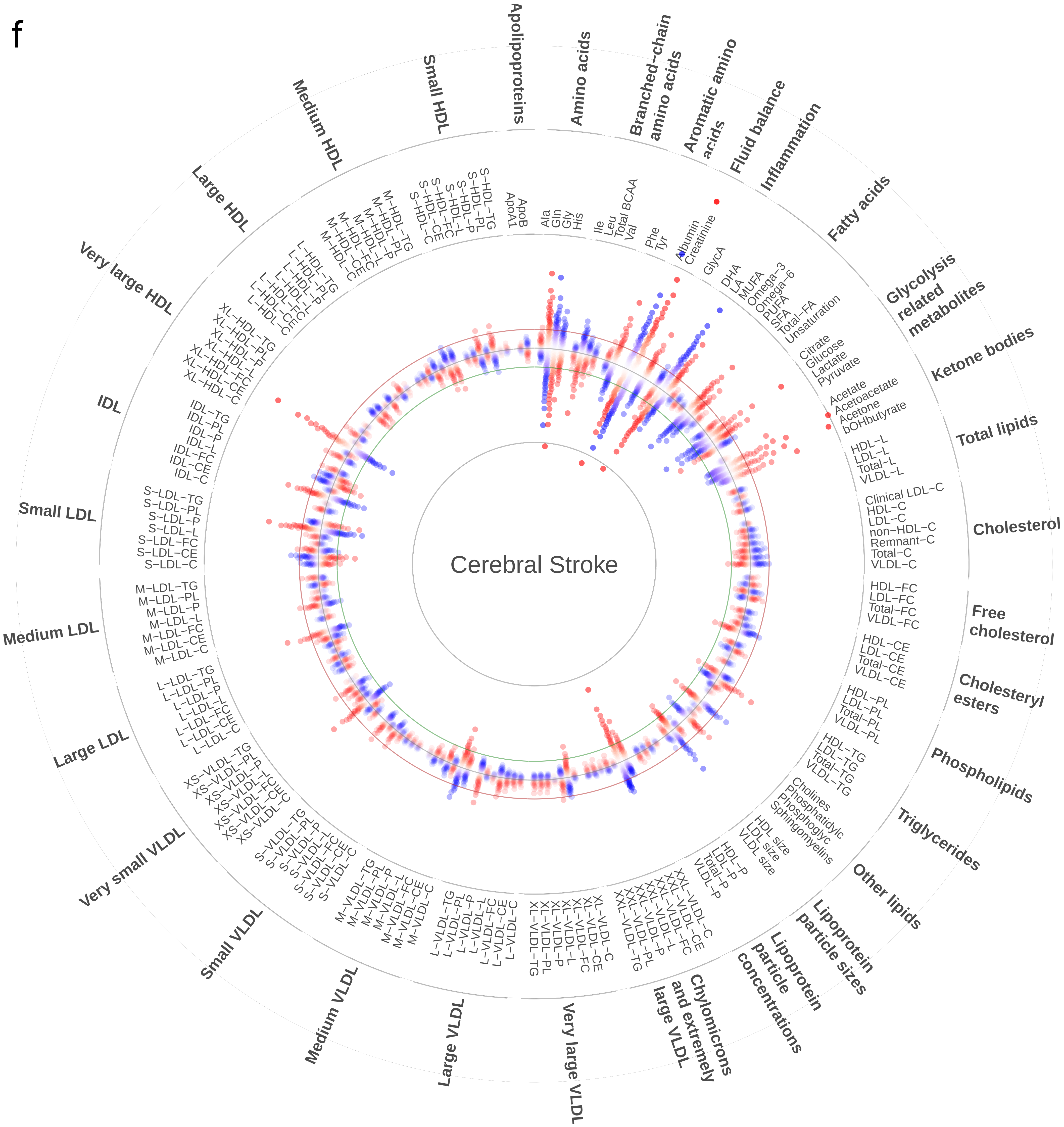

f
Cerebral Stroke
Apolipoproteins
Amino acids
Branched−chain amino acids
Aromatic amino acids
Fluid balance
Inflammation
Fatty acids
Glycolysis related metabolites
Ketone bodies
Total lipids
Cholesterol
Free cholesterol
Cholesteryl esters
Phospholipids
Triglycerides
Other lipids
Lipoprotein particle sizes
Lipoprotein particle concentrations
Chylomicrons and extremely large VLDL
Very large VLDL
Large VLDL
Medium VLDL
Small VLDL
Very small VLDL
Large LDL
Medium LDL
Small LDL
IDL
Very large HDL
Large HDL
Medium HDL
Small HDL
ApoB
ApoA1
Ala
Gln
Gly
His
Ile
Leu
Total BCAA
Val
Phe
Tyr
Albumin
Creatinine
GlycA
DHA
LA
MUFA
Omega−3
Omega−6
PUFA
SFA
Total−FA
Unsaturation
Citrate
Glucose
Lactate
Pyruvate
Acetate
Acetoacetate
Acetone
bOHbutyrate
HDL−L
LDL−L
Total−L
VLDL−L
Clinical LDL−C
HDL−C
LDL−C
non−HDL−C
Remnant−C
Total−C
VLDL−C
HDL−FC
LDL−FC
Total−FC
VLDL−FC
HDL−CE
LDL−CE
Total−CE
VLDL−CE
HDL−PL
LDL−PL
Total−PL
VLDL−PL
HDL−TG
LDL−TG
Total−TG
VLDL−TG
Cholines
Phosphatidylc
Phosphoglyc
Sphingomyelins
HDL size
LDL size
VLDL size
HDL−P
LDL−P
Total−P
VLDL−P
XXL−VLDL−C
XXL−VLDL−CE
XXL−VLDL−FC
XXL−VLDL−L
XXL−VLDL−P
XXL−VLDL−PL
XXL−VLDL−TG
XL−VLDL−C
XL−VLDL−CE
XL−VLDL−FC
XL−VLDL−L
XL−VLDL−P
XL−VLDL−PL
XL−VLDL−TG
L−VLDL−C
L−VLDL−CE
L−VLDL−FC
L−VLDL−L
L−VLDL−P
L−VLDL−PL
L−VLDL−TG
M−VLDL−C
M−VLDL−CE
M−VLDL−FC
M−VLDL−L
M−VLDL−P
M−VLDL−PL
M−VLDL−TG
S−VLDL−C
S−VLDL−CE
S−VLDL−FC
S−VLDL−L
S−VLDL−P
S−VLDL−PL
S−VLDL−TG
XS−VLDL−C
XS−VLDL−CE
XS−VLDL−FC
XS−VLDL−L
XS−VLDL−P
XS−VLDL−PL
XS−VLDL−TG
L−LDL−C
L−LDL−CE
L−LDL−FC
L−LDL−L
L−LDL−P
L−LDL−PL
L−LDL−TG
M−LDL−C
M−LDL−CE
M−LDL−FC
M−LDL−L
M−LDL−P
M−LDL−PL
M−LDL−TG
S−LDL−C
S−LDL−CE
S−LDL−FC
S−LDL−L
S−LDL−P
S−LDL−PL
S−LDL−TG
IDL−C
IDL−CE
IDL−FC
IDL−L
IDL−P
IDL−PL
IDL−TG
XL−HDL−C
XL−HDL−CE
XL−HDL−FC
XL−HDL−L
XL−HDL−P
XL−HDL−PL
XL−HDL−TG
L−HDL−C
L−HDL−CE
L−HDL−FC
L−HDL−L
L−HDL−P
L−HDL−PL
L−HDL−TG
M−HDL−C
M−HDL−CE
M−HDL−FC
M−HDL−L
M−HDL−P
M−HDL−PL
M−HDL−TG
S−HDL−C
S−HDL−CE
S−HDL−FC
S−HDL−L
S−HDL−P
S−HDL−PL
S−HDL−TG

g

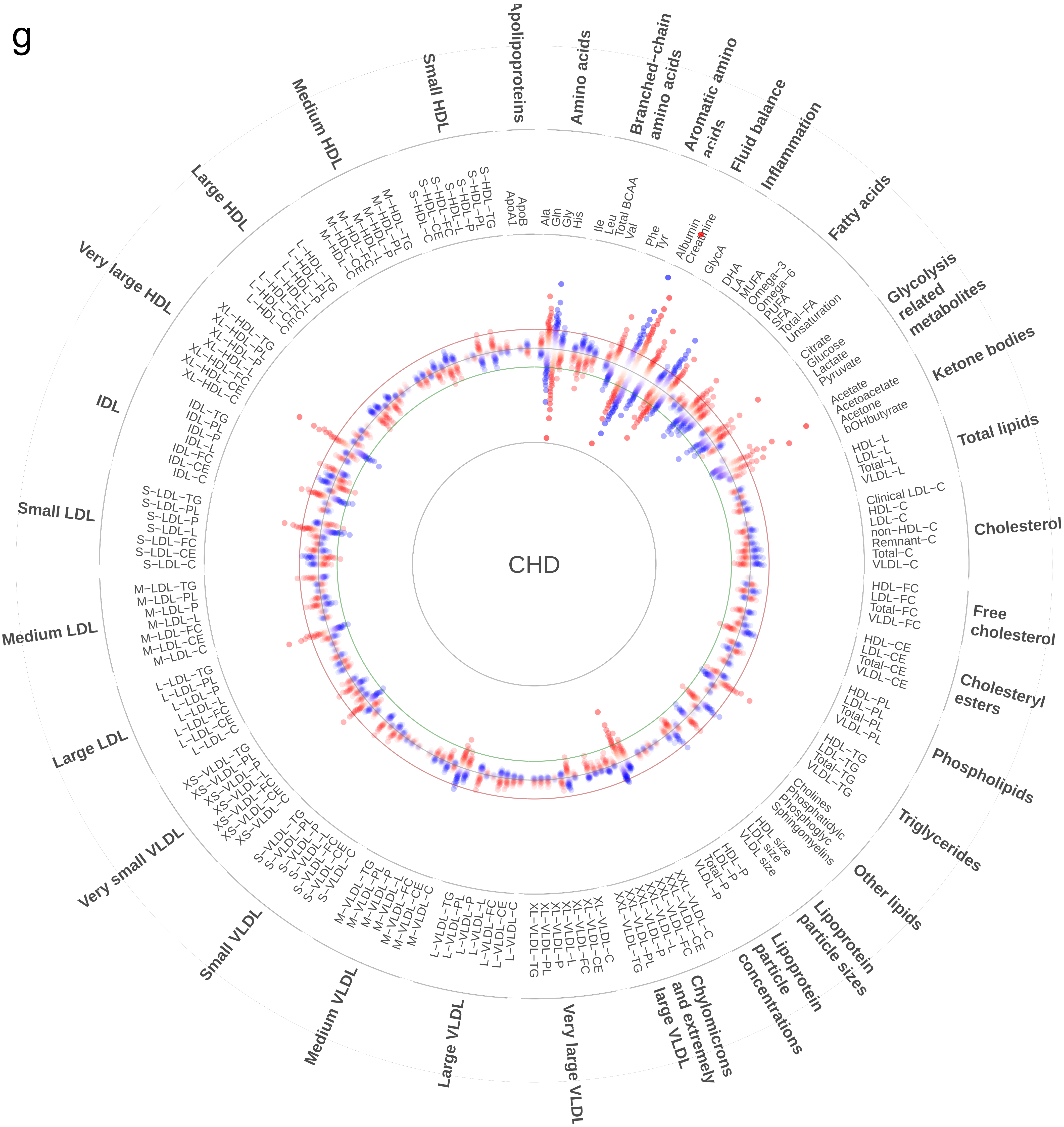

CHD
Apolipoproteins
Amino acids
Branched−chain amino acids
Aromatic amino acids
Fluid balance
Inflammation
Fatty acids
Glycolysis related metabolites
Ketone bodies
Total lipids
Cholesterol
Free cholesterol
Cholesteryl esters
Phospholipids
Triglycerides
Other lipids
Lipoprotein particle sizes
Lipoprotein particle concentrations
Chylomicrons and extremely large VLDL
Very large VLDL
Large VLDL
Medium VLDL
Small VLDL
Very small VLDL
Large LDL
Medium LDL
Small LDL
IDL
Very large HDL
Large HDL
Medium HDL
Small HDL
ApoB
ApoA1
Ala
Gln
Gly
His
Ile
Leu
Total BCAA
Val
Phe
Tyr
Albumin
Creatinine
GlycA
DHA
LA
MUFA
Omega−3
Omega−6
PUFA
SFA
Total−FA
Unsaturation
Citrate
Glucose
Lactate
Pyruvate
Acetate
Acetoacetate
Acetone
bOHbutyrate
HDL−L
LDL−L
Total−L
VLDL−L
Clinical LDL−C
HDL−C
LDL−C
non−HDL−C
Remnant−C
Total−C
VLDL−C
HDL−FC
LDL−FC
Total−FC
VLDL−FC
HDL−CE
LDL−CE
Total−CE
VLDL−CE
HDL−PL
LDL−PL
Total−PL
VLDL−PL
HDL−TG
LDL−TG
Total−TG
VLDL−TG
Cholines
Phosphatidylc
Phosphoglyc
Sphingomyelins
HDL size
LDL size
VLDL size
HDL−P
LDL−P
Total−P
VLDL−P
XXL−VLDL−C
XXL−VLDL−CE
XXL−VLDL−FC
XXL−VLDL−L
XXL−VLDL−P
XXL−VLDL−PL
XXL−VLDL−TG
XL−VLDL−C
XL−VLDL−CE
XL−VLDL−FC
XL−VLDL−L
XL−VLDL−P
XL−VLDL−PL
XL−VLDL−TG
L−VLDL−C
L−VLDL−CE
L−VLDL−FC
L−VLDL−L
L−VLDL−P
L−VLDL−PL
L−VLDL−TG
M−VLDL−C
M−VLDL−CE
M−VLDL−FC
M−VLDL−L
M−VLDL−P
M−VLDL−PL
M−VLDL−TG
S−VLDL−C
S−VLDL−CE
S−VLDL−FC
S−VLDL−L
S−VLDL−P
S−VLDL−PL
S−VLDL−TG
XS−VLDL−C
XS−VLDL−CE
XS−VLDL−FC
XS−VLDL−L
XS−VLDL−P
XS−VLDL−PL
XS−VLDL−TG
L−LDL−C
L−LDL−CE
L−LDL−FC
L−LDL−L
L−LDL−P
L−LDL−PL
L−LDL−TG
M−LDL−C
M−LDL−CE
M−LDL−FC
M−LDL−L
M−LDL−P
M−LDL−PL
M−LDL−TG
S−LDL−C
S−LDL−CE
S−LDL−FC
S−LDL−L
S−LDL−P
S−LDL−PL
S−LDL−TG
IDL−C
IDL−CE
IDL−FC
IDL−L
IDL−P
IDL−PL
IDL−TG
XL−HDL−C
XL−HDL−CE
XL−HDL−FC
XL−HDL−L
XL−HDL−P
XL−HDL−PL
XL−HDL−TG
L−HDL−C
L−HDL−CE
L−HDL−FC
L−HDL−L
L−HDL−P
L−HDL−PL
L−HDL−TG
M−HDL−C
M−HDL−CE
M−HDL−FC
M−HDL−L
M−HDL−P
M−HDL−PL
M−HDL−TG
S−HDL−C
S−HDL−CE
S−HDL−FC
S−HDL−L
S−HDL−P
S−HDL−PL
S−HDL−TG

h

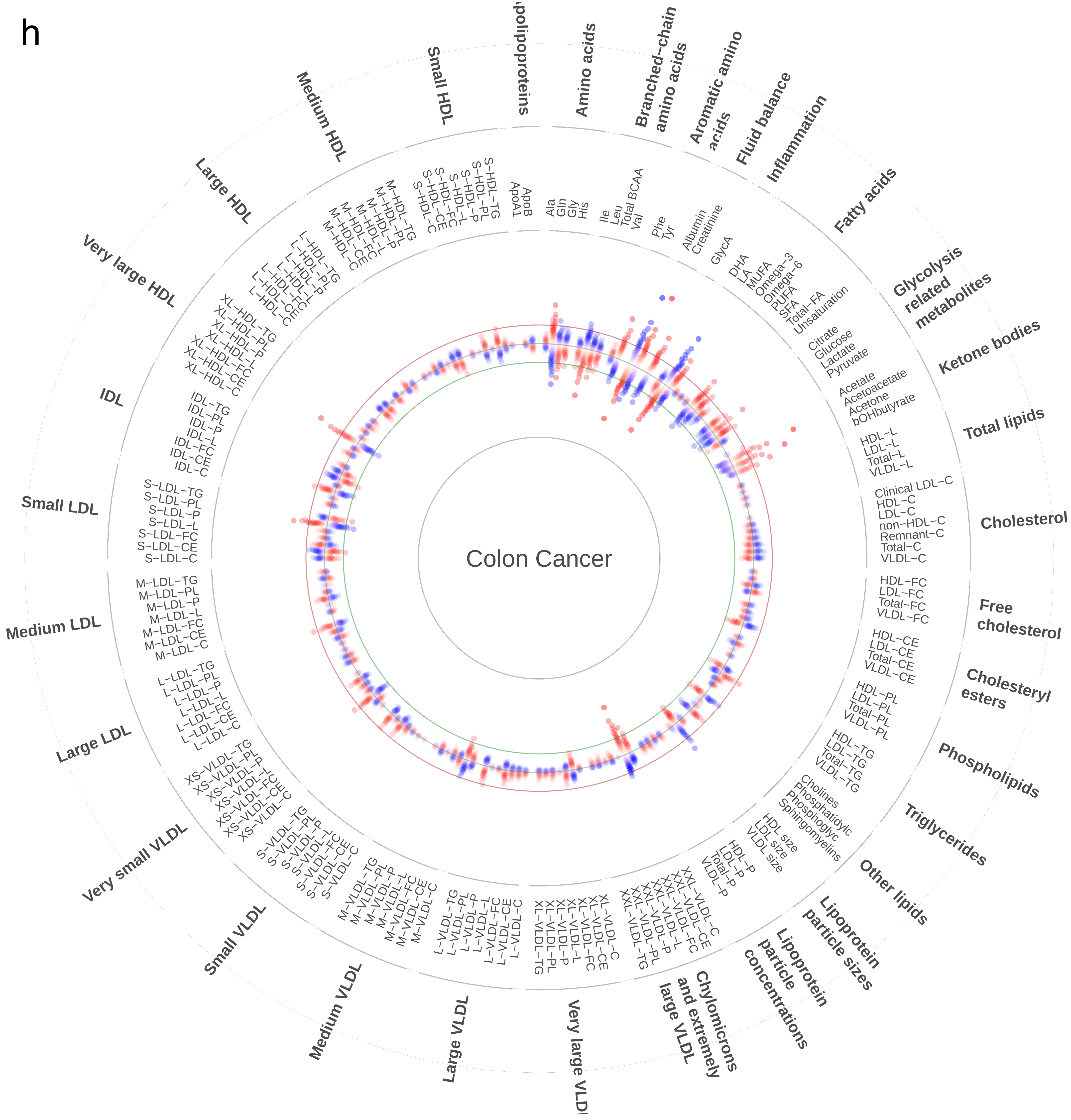

Colon Cancer
Apolipoproteins
ApoB
ApoA1
Amino acids
Ala
Gln
Gly
His
Branched−chain amino acids
Ile
Leu
Total BCAA
Val
Aromatic amino acids
Phe
Tyr
Fluid balance
Albumin
Creatinine
Inflammation
GlycA
Fatty acids
DHA
LA
MUFA
Omega−3
Omega−6
PUFA
SFA
Total−FA
Unsaturation
Glycolysis related metabolites
Citrate
Glucose
Lactate
Pyruvate
Ketone bodies
Acetate
Acetoacetate
Acetone
bOHbutyrate
Total lipids
HDL−L
LDL−L
Total−L
VLDL−L
Cholesterol
Clinical LDL−C
HDL−C
LDL−C
non−HDL−C
Remnant−C
Total−C
VLDL−C
Free cholesterol
HDL−FC
LDL−FC
Total−FC
VLDL−FC
Cholesteryl esters
HDL−CE
LDL−CE
Total−CE
VLDL−CE
Phospholipids
HDL−PL
LDL−PL
Total−PL
VLDL−PL
Triglycerides
HDL−TG
LDL−TG
Total−TG
VLDL−TG
Other lipids
Cholines
Phosphatidylc
Phosphoglyc
Sphingomyelins
Lipoprotein particle sizes
HDL size
LDL size
VLDL size
Lipoprotein particle concentrations
HDL−P
LDL−P
Total−P
VLDL−P
Chylomicrons and extremely large VLDL
XXL−VLDL−C
XXL−VLDL−CE
XXL−VLDL−FC
XXL−VLDL−L
XXL−VLDL−P
XXL−VLDL−PL
XXL−VLDL−TG
Very large VLDL
XL−VLDL−C
XL−VLDL−CE
XL−VLDL−FC
XL−VLDL−L
XL−VLDL−P
XL−VLDL−PL
XL−VLDL−TG
Large VLDL
L−VLDL−TG
L−VLDL−PL
L−VLDL−P
L−VLDL−L
L−VLDL−FC
L−VLDL−CE
L−VLDL−C
Medium VLDL
M−VLDL−TG
M−VLDL−PL
M−VLDL−P
M−VLDL−L
M−VLDL−FC
M−VLDL−CE
M−VLDL−C
Small VLDL
S−VLDL−TG
S−VLDL−PL
S−VLDL−P
S−VLDL−L
S−VLDL−FC
S−VLDL−CE
S−VLDL−C
Very small VLDL
XS−VLDL−TG
XS−VLDL−PL
XS−VLDL−P
XS−VLDL−L
XS−VLDL−FC
XS−VLDL−CE
XS−VLDL−C
Large LDL
L−LDL−TG
L−LDL−PL
L−LDL−P
L−LDL−L
L−LDL−FC
L−LDL−CE
L−LDL−C
Medium LDL
M−LDL−TG
M−LDL−PL
M−LDL−P
M−LDL−L
M−LDL−FC
M−LDL−CE
M−LDL−C
Small LDL
S−LDL−TG
S−LDL−PL
S−LDL−P
S−LDL−L
S−LDL−FC
S−LDL−CE
S−LDL−C
IDL
IDL−TG
IDL−PL
IDL−P
IDL−L
IDL−FC
IDL−CE
IDL−C
Very large HDL
XL−HDL−TG
XL−HDL−PL
XL−HDL−P
XL−HDL−L
XL−HDL−FC
XL−HDL−CE
XL−HDL−C
Large HDL
L−HDL−TG
L−HDL−PL
L−HDL−P
L−HDL−L
L−HDL−FC
L−HDL−CE
L−HDL−C
Medium HDL
M−HDL−TG
M−HDL−PL
M−HDL−P
M−HDL−L
M−HDL−FC
M−HDL−CE
M−HDL−C
Small HDL
S−HDL−TG
S−HDL−PL
S−HDL−P
S−HDL−L
S−HDL−FC
S−HDL−CE
S−HDL−C

i

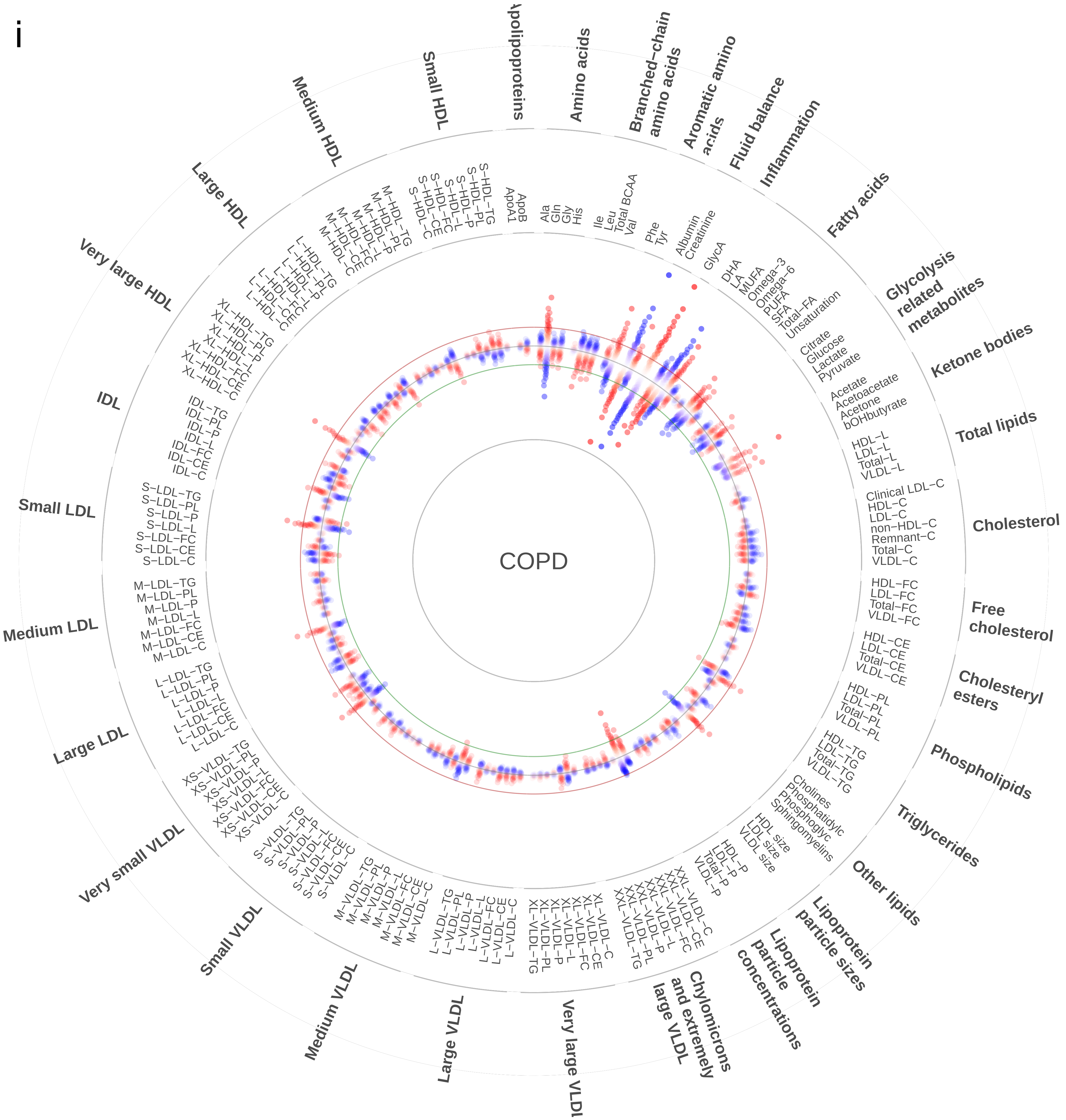
COPD
Apolipoproteins
Amino acids
Branched−chain amino acids
Aromatic amino acids
Fluid balance
Inflammation
Fatty acids
Glycolysis related metabolites
Ketone bodies
Total lipids
Cholesterol
Free cholesterol
Cholesteryl esters
Phospholipids
Triglycerides
Other lipids
Lipoprotein particle sizes
Lipoprotein particle concentrations
Chylomicrons and extremely large VLDL
Very large VLDL
Large VLDL
Medium VLDL
Small VLDL
Very small VLDL
Large LDL
Medium LDL
Small LDL
IDL
Very large HDL
Large HDL
Medium HDL
Small HDL
ApoB
ApoA1
Ala
Gln
Gly
His
Ile
Leu
Total BCAA
Val
Phe
Tyr
Albumin
Creatinine
GlycA
DHA
LA
MUFA
Omega−3
Omega−6
PUFA
SFA
Total−FA
Unsaturation
Citrate
Glucose
Lactate
Pyruvate
Acetate
Acetoacetate
Acetone
bOHbutyrate
HDL−L
LDL−L
Total−L
VLDL−L
Clinical LDL−C
HDL−C
LDL−C
non−HDL−C
Remnant−C
Total−C
VLDL−C
HDL−FC
LDL−FC
Total−FC
VLDL−FC
HDL−CE
LDL−CE
Total−CE
VLDL−CE
HDL−PL
LDL−PL
Total−PL
VLDL−PL
HDL−TG
LDL−TG
Total−TG
VLDL−TG
Cholines
Phosphatidylc
Phosphoglyc
Sphingomyelins
HDL size
LDL size
VLDL size
HDL−P
LDL−P
Total−P
VLDL−P
XXL−VLDL−C
XXL−VLDL−CE
XXL−VLDL−FC
XXL−VLDL−L
XXL−VLDL−P
XXL−VLDL−PL
XXL−VLDL−TG
XL−VLDL−C
XL−VLDL−CE
XL−VLDL−FC
XL−VLDL−L
XL−VLDL−P
XL−VLDL−PL
XL−VLDL−TG
L−VLDL−TG
L−VLDL−PL
L−VLDL−P
L−VLDL−L
L−VLDL−FC
L−VLDL−CE
L−VLDL−C
M−VLDL−TG
M−VLDL−PL
M−VLDL−P
M−VLDL−L
M−VLDL−FC
M−VLDL−CE
M−VLDL−C
S−VLDL−TG
S−VLDL−PL
S−VLDL−P
S−VLDL−L
S−VLDL−FC
S−VLDL−CE
S−VLDL−C
XS−VLDL−TG
XS−VLDL−PL
XS−VLDL−P
XS−VLDL−L
XS−VLDL−FC
XS−VLDL−CE
XS−VLDL−C
L−LDL−TG
L−LDL−PL
L−LDL−P
L−LDL−L
L−LDL−FC
L−LDL−CE
L−LDL−C
M−LDL−TG
M−LDL−PL
M−LDL−P
M−LDL−L
M−LDL−FC
M−LDL−CE
M−LDL−C
S−LDL−TG
S−LDL−PL
S−LDL−P
S−LDL−L
S−LDL−FC
S−LDL−CE
S−LDL−C
IDL−TG
IDL−PL
IDL−P
IDL−L
IDL−FC
IDL−CE
IDL−C
XL−HDL−TG
XL−HDL−PL
XL−HDL−P
XL−HDL−L
XL−HDL−FC
XL−HDL−CE
XL−HDL−C
L−HDL−TG
L−HDL−PL
L−HDL−P
L−HDL−L
L−HDL−FC
L−HDL−CE
L−HDL−C
M−HDL−TG
M−HDL−PL
M−HDL−P
M−HDL−L
M−HDL−FC
M−HDL−CE
M−HDL−C
S−HDL−TG
S−HDL−PL
S−HDL−P
S−HDL−L
S−HDL−FC
S−HDL−CE
S−HDL−C

j

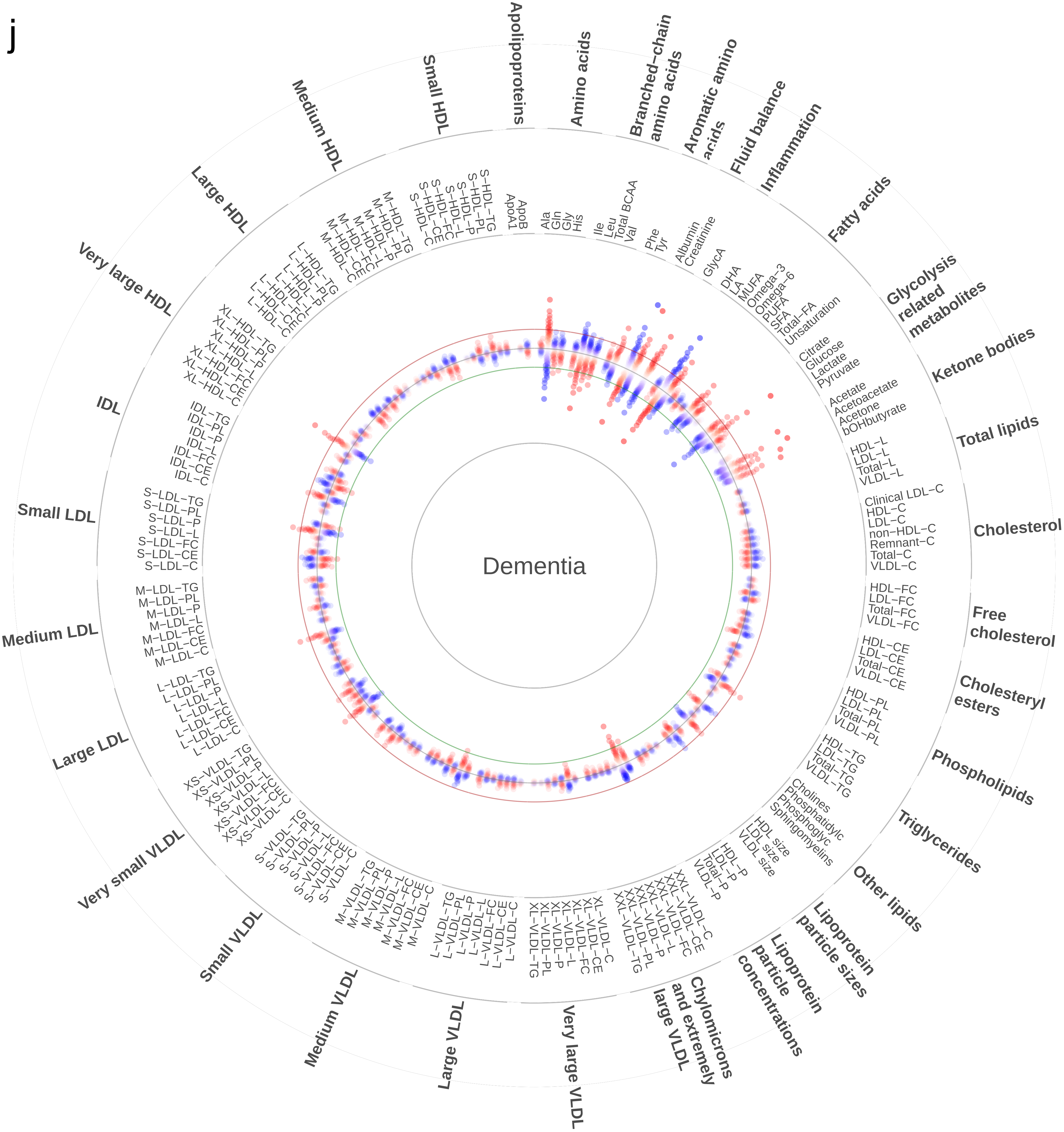
Dementia
Apolipoproteins
Amino acids
Branched−chain amino acids
Aromatic amino acids
Fluid balance
Inflammation
Fatty acids
Glycolysis related metabolites
Ketone bodies
Total lipids
Cholesterol
Free cholesterol
Cholesteryl esters
Phospholipids
Triglycerides
Other lipids
Lipoprotein particle sizes
Lipoprotein particle concentrations
Chylomicrons and extremely large VLDL
Very large VLDL
Large VLDL
Medium VLDL
Small VLDL
Very small VLDL
Large LDL
Medium LDL
Small LDL
IDL
Very large HDL
Large HDL
Medium HDL
Small HDL
ApoB
ApoA1
Ala
Gln
Gly
His
Ile
Leu
Total BCAA
Val
Phe
Tyr
Albumin
Creatinine
GlycA
DHA
LA
MUFA
Omega−3
Omega−6
PUFA
SFA
Total−FA
Unsaturation
Citrate
Glucose
Lactate
Pyruvate
Acetate
Acetoacetate
Acetone
bOHbutyrate
HDL−L
LDL−L
Total−L
VLDL−L
Clinical LDL−C
HDL−C
LDL−C
non−HDL−C
Remnant−C
Total−C
VLDL−C
HDL−FC
LDL−FC
Total−FC
VLDL−FC
HDL−CE
LDL−CE
Total−CE
VLDL−CE
HDL−PL
LDL−PL
Total−PL
VLDL−PL
HDL−TG
LDL−TG
Total−TG
VLDL−TG
Cholines
Phosphatidylc
Phosphoglyc
Sphingomyelins
HDL size
LDL size
VLDL size
HDL−P
LDL−P
Total−P
VLDL−P
XXL−VLDL−C
XXL−VLDL−CE
XXL−VLDL−FC
XXL−VLDL−L
XXL−VLDL−P
XXL−VLDL−PL
XXL−VLDL−TG
XL−VLDL−C
XL−VLDL−CE
XL−VLDL−FC
XL−VLDL−L
XL−VLDL−P
XL−VLDL−PL
XL−VLDL−TG
L−VLDL−C
L−VLDL−CE
L−VLDL−FC
L−VLDL−L
L−VLDL−P
L−VLDL−PL
L−VLDL−TG
M−VLDL−C
M−VLDL−CE
M−VLDL−FC
M−VLDL−L
M−VLDL−P
M−VLDL−PL
M−VLDL−TG
S−VLDL−C
S−VLDL−CE
S−VLDL−FC
S−VLDL−L
S−VLDL−P
S−VLDL−PL
S−VLDL−TG
XS−VLDL−C
XS−VLDL−CE
XS−VLDL−FC
XS−VLDL−L
XS−VLDL−P
XS−VLDL−PL
XS−VLDL−TG
L−LDL−C
L−LDL−CE
L−LDL−FC
L−LDL−L
L−LDL−P
L−LDL−PL
L−LDL−TG
M−LDL−C
M−LDL−CE
M−LDL−FC
M−LDL−L
M−LDL−P
M−LDL−PL
M−LDL−TG
S−LDL−C
S−LDL−CE
S−LDL−FC
S−LDL−L
S−LDL−P
S−LDL−PL
S−LDL−TG
IDL−C
IDL−CE
IDL−FC
IDL−L
IDL−P
IDL−PL
IDL−TG
XL−HDL−CE
XL−HDL−C
XL−HDL−FC
XL−HDL−L
XL−HDL−P
XL−HDL−PL
XL−HDL−TG
L−HDL−C
L−HDL−CE
L−HDL−FC
L−HDL−L
L−HDL−P
L−HDL−PL
L−HDL−TG
M−HDL−C
M−HDL−CE
M−HDL−FC
M−HDL−L
M−HDL−P
M−HDL−PL
M−HDL−TG
S−HDL−C
S−HDL−CE
S−HDL−FC
S−HDL−L
S−HDL−P
S−HDL−PL
S−HDL−TG

k

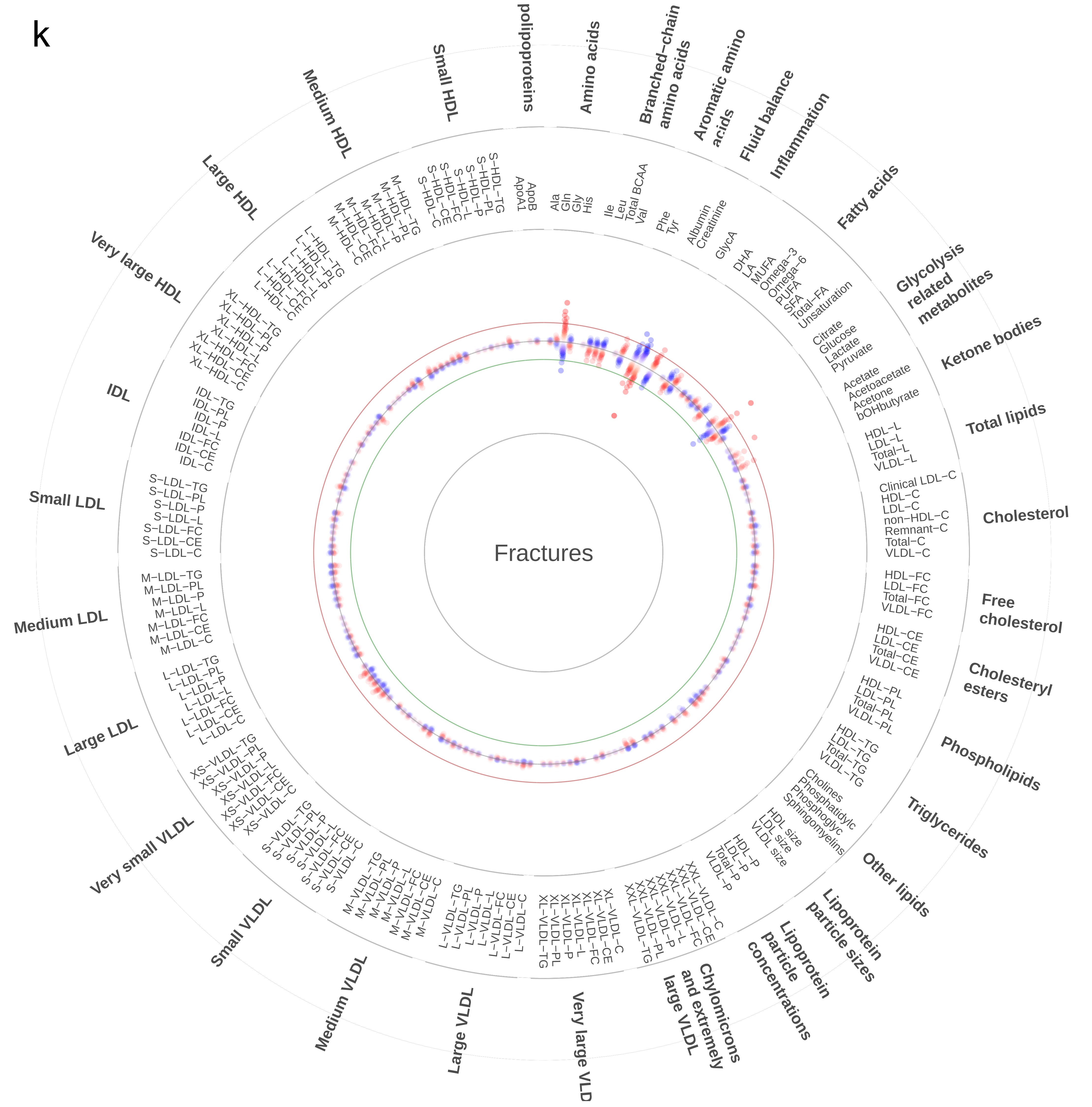
Fractures
Amino acids
Branched−chain amino acids
Aromatic amino acids
Fluid balance
Inflammation
Fatty acids
Glycolysis related metabolites
Ketone bodies
Total lipids
Cholesterol
Free cholesterol
Cholesteryl esters
Phospholipids
Triglycerides
Other lipids
Lipoprotein particle sizes
Lipoprotein particle concentrations
Chylomicrons and extremely large VLDL
Very large VLDL
Large VLDL
Medium VLDL
Small VLDL
Very small VLDL
Large LDL
Medium LDL
Small LDL
IDL
Very large HDL
Large HDL
Medium HDL
Small HDL
polipoproteins
ApoB
ApoA1
Ala
Gln
Gly
His
Ile
Leu
Total BCAA
Val
Phe
Tyr
Albumin
Creatinine
GlycA
DHA
LA
MUFA
Omega−3
Omega−6
PUFA
SFA
Total−FA
Unsaturation
Citrate
Glucose
Lactate
Pyruvate
Acetate
Acetoacetate
Acetone
bOHbutyrate
HDL−L
LDL−L
Total−L
VLDL−L
Clinical LDL−C
HDL−C
LDL−C
non−HDL−C
Remnant−C
Total−C
VLDL−C
HDL−FC
LDL−FC
Total−FC
VLDL−FC
HDL−CE
LDL−CE
Total−CE
VLDL−CE
HDL−PL
LDL−PL
Total−PL
VLDL−PL
HDL−TG
LDL−TG
Total−TG
VLDL−TG
Cholines
Phosphatidylc
Phosphoglyc
Sphingomyelins
HDL size
LDL size
VLDL size
HDL−P
LDL−P
Total−P
VLDL−P
XXL−VLDL−C
XXL−VLDL−CE
XXL−VLDL−FC
XXL−VLDL−L
XXL−VLDL−P
XXL−VLDL−PL
XXL−VLDL−TG
XL−VLDL−C
XL−VLDL−CE
XL−VLDL−FC
XL−VLDL−L
XL−VLDL−P
XL−VLDL−PL
XL−VLDL−TG
L−VLDL−TG
L−VLDL−PL
L−VLDL−P
L−VLDL−L
L−VLDL−FC
L−VLDL−CE
L−VLDL−C
M−VLDL−TG
M−VLDL−PL
M−VLDL−P
M−VLDL−L
M−VLDL−FC
M−VLDL−CE
M−VLDL−C
S−VLDL−TG
S−VLDL−PL
S−VLDL−P
S−VLDL−L
S−VLDL−FC
S−VLDL−CE
S−VLDL−C
XS−VLDL−TG
XS−VLDL−PL
XS−VLDL−P
XS−VLDL−L
XS−VLDL−FC
XS−VLDL−CE
XS−VLDL−C
L−LDL−TG
L−LDL−PL
L−LDL−P
L−LDL−L
L−LDL−FC
L−LDL−CE
L−LDL−C
M−LDL−TG
M−LDL−PL
M−LDL−P
M−LDL−L
M−LDL−FC
M−LDL−CE
M−LDL−C
S−LDL−TG
S−LDL−PL
S−LDL−P
S−LDL−L
S−LDL−FC
S−LDL−CE
S−LDL−C
IDL−TG
IDL−PL
IDL−P
IDL−L
IDL−FC
IDL−CE
IDL−C
XL−HDL−TG
XL−HDL−PL
XL−HDL−P
XL−HDL−L
XL−HDL−FC
XL−HDL−CE
XL−HDL−C
L−HDL−TG
L−HDL−PL
L−HDL−P
L−HDL−L
L−HDL−FC
L−HDL−CE
L−HDL−C
M−HDL−TG
M−HDL−PL
M−HDL−P
M−HDL−L
M−HDL−FC
M−HDL−CE
M−HDL−C
S−HDL−TG
S−HDL−PL
S−HDL−P
S−HDL−L
S−HDL−FC
S−HDL−CE
S−HDL−C

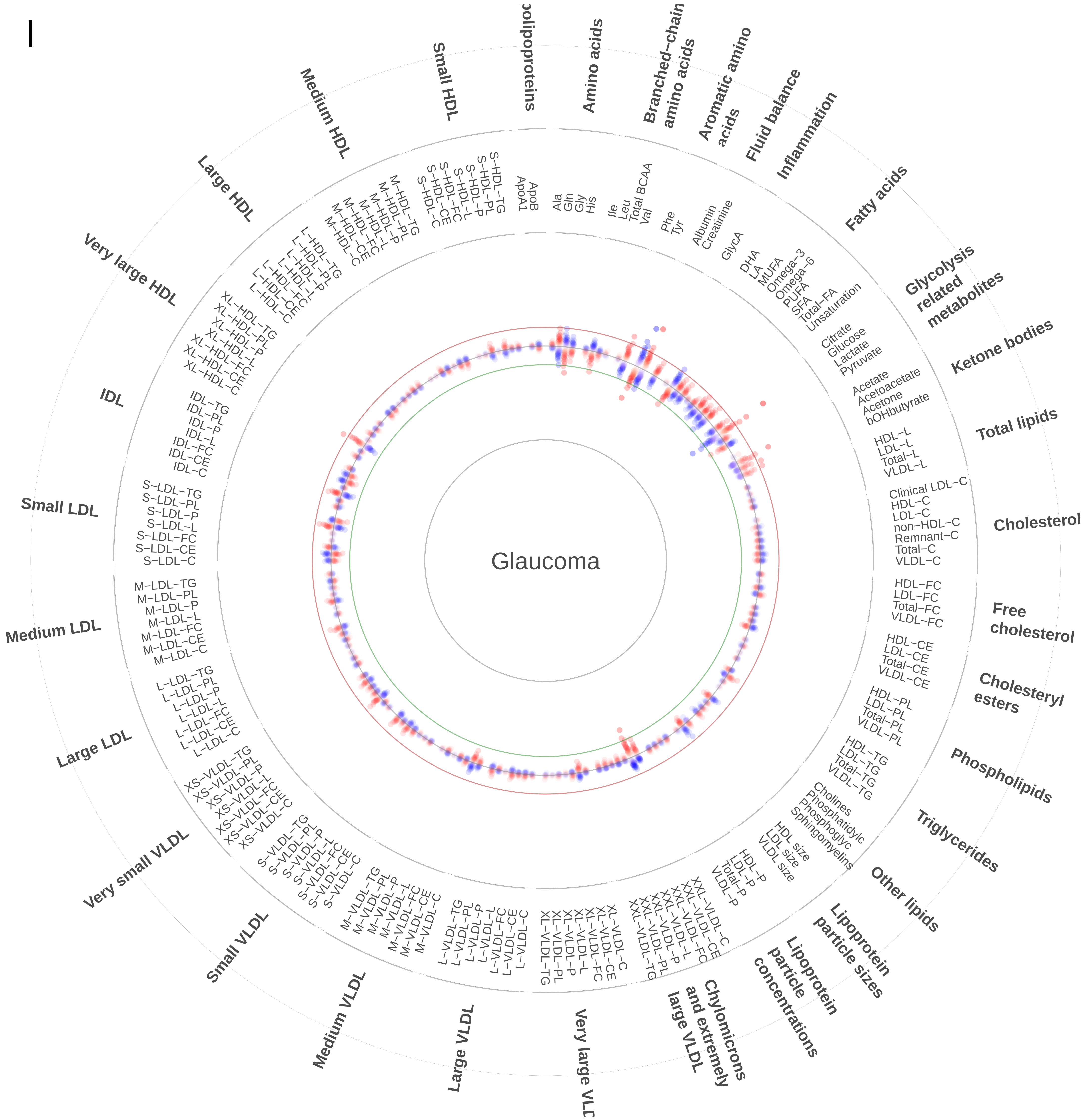

I
Glaucoma
Apolipoproteins
Amino acids
Branched−chain amino acids
Aromatic amino acids
Fluid balance
Inflammation
Fatty acids
Glycolysis related metabolites
Ketone bodies
Total lipids
Cholesterol
Free cholesterol
Cholesteryl esters
Phospholipids
Triglycerides
Other lipids
Lipoprotein particle sizes
Lipoprotein particle concentrations
Chylomicrons and extremely large VLDL
Very large VLDL
Large VLDL
Medium VLDL
Small VLDL
Very small VLDL
Large LDL
Medium LDL
Small LDL
IDL
Very large HDL
Large HDL
Medium HDL
Small HDL
ApoB
ApoA1
Ala
Gln
Gly
His
Ile
Leu
Total BCAA
Val
Phe
Tyr
Albumin
Creatinine
GlycA
DHA
LA
MUFA
Omega−3
Omega−6
PUFA
SFA
Total−FA
Unsaturation
Citrate
Glucose
Lactate
Pyruvate
Acetate
Acetoacetate
Acetone
bOHbutyrate
HDL−L
LDL−L
Total−L
VLDL−L
Clinical LDL−C
HDL−C
LDL−C
non−HDL−C
Remnant−C
Total−C
VLDL−C
HDL−FC
LDL−FC
Total−FC
VLDL−FC
HDL−CE
LDL−CE
Total−CE
VLDL−CE
HDL−PL
LDL−PL
Total−PL
VLDL−PL
HDL−TG
LDL−TG
Total−TG
VLDL−TG
Cholines
Phosphatidylc
Phosphoglyc
Sphingomyelins
HDL size
LDL size
VLDL size
HDL−P
LDL−P
Total−P
VLDL−P
XXL−VLDL−C
XXL−VLDL−CE
XXL−VLDL−FC
XXL−VLDL−L
XXL−VLDL−P
XXL−VLDL−PL
XXL−VLDL−TG
XL−VLDL−C
XL−VLDL−CE
XL−VLDL−FC
XL−VLDL−L
XL−VLDL−P
XL−VLDL−PL
XL−VLDL−TG
L−VLDL−TG
L−VLDL−PL
L−VLDL−P
L−VLDL−L
L−VLDL−FC
L−VLDL−CE
L−VLDL−C
M−VLDL−TG
M−VLDL−PL
M−VLDL−P
M−VLDL−L
M−VLDL−FC
M−VLDL−CE
M−VLDL−C
S−VLDL−TG
S−VLDL−PL
S−VLDL−P
S−VLDL−L
S−VLDL−FC
S−VLDL−CE
S−VLDL−C
XS−VLDL−TG
XS−VLDL−PL
XS−VLDL−P
XS−VLDL−L
XS−VLDL−FC
XS−VLDL−CE
XS−VLDL−C
L−LDL−TG
L−LDL−PL
L−LDL−P
L−LDL−L
L−LDL−FC
L−LDL−CE
L−LDL−C
M−LDL−TG
M−LDL−PL
M−LDL−P
M−LDL−L
M−LDL−FC
M−LDL−CE
M−LDL−C
S−LDL−TG
S−LDL−PL
S−LDL−P
S−LDL−L
S−LDL−FC
S−LDL−CE
S−LDL−C
IDL−TG
IDL−PL
IDL−P
IDL−L
IDL−FC
IDL−CE
IDL−C
XL−HDL−TG
XL−HDL−PL
XL−HDL−P
XL−HDL−L
XL−HDL−FC
XL−HDL−CE
XL−HDL−C
L−HDL−TG
L−HDL−PL
L−HDL−P
L−HDL−L
L−HDL−FC
L−HDL−CE
L−HDL−C
M−HDL−TG
M−HDL−PL
M−HDL−P
M−HDL−L
M−HDL−FC
M−HDL−CE
M−HDL−C
S−HDL−TG
S−HDL−PL
S−HDL−P
S−HDL−L
S−HDL−FC
S−HDL−CE
S−HDL−C

m

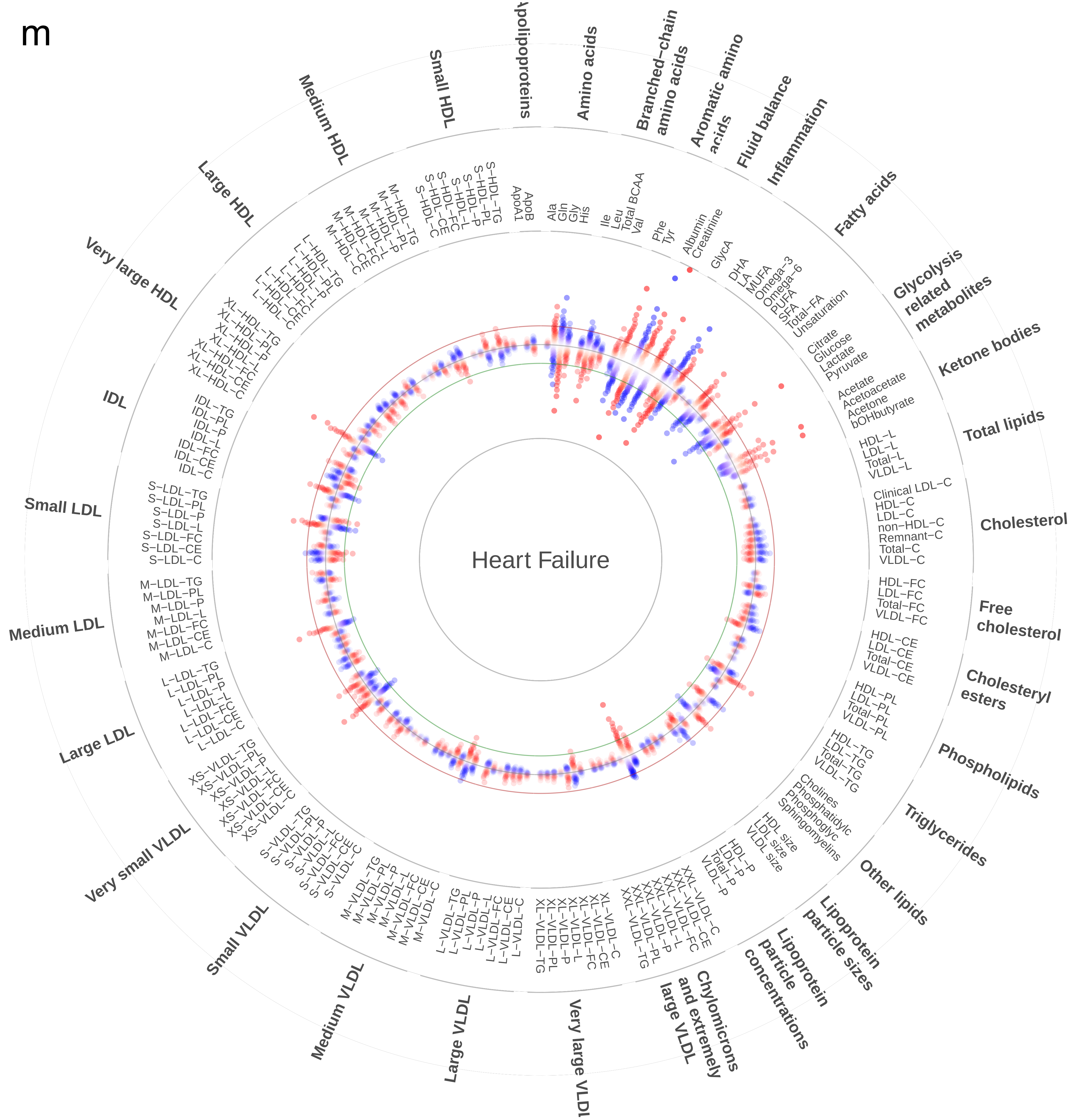
Heart Failure
Apolipoproteins
ApoB
ApoA1
Amino acids
Ala
Gln
Gly
His
Branched−chain amino acids
Ile
Leu
Total BCAA
Val
Aromatic amino acids
Phe
Tyr
Fluid balance
Albumin
Creatinine
Inflammation
GlycA
Fatty acids
DHA
LA
MUFA
Omega−3
Omega−6
PUFA
SFA
Total−FA
Unsaturation
Glycolysis related metabolites
Citrate
Glucose
Lactate
Pyruvate
Ketone bodies
Acetate
Acetoacetate
Acetone
bOHbutyrate
Total lipids
HDL−L
LDL−L
Total−L
VLDL−L
Cholesterol
Clinical LDL−C
HDL−C
LDL−C
non−HDL−C
Remnant−C
Total−C
VLDL−C
Free cholesterol
HDL−FC
LDL−FC
Total−FC
VLDL−FC
Cholesteryl esters
HDL−CE
LDL−CE
Total−CE
VLDL−CE
Phospholipids
HDL−PL
LDL−PL
Total−PL
VLDL−PL
Triglycerides
HDL−TG
LDL−TG
Total−TG
VLDL−TG
Other lipids
Cholines
Phosphatidylc
Phosphoglyc
Sphingomyelins
Lipoprotein particle sizes
HDL size
LDL size
VLDL size
Lipoprotein particle concentrations
HDL−P
LDL−P
Total−P
VLDL−P
Chylomicrons and extremely large VLDL
XXL−VLDL−C
XXL−VLDL−CE
XXL−VLDL−FC
XXL−VLDL−L
XXL−VLDL−P
XXL−VLDL−PL
XXL−VLDL−TG
Very large VLDL
XL−VLDL−C
XL−VLDL−CE
XL−VLDL−FC
XL−VLDL−L
XL−VLDL−P
XL−VLDL−PL
XL−VLDL−TG
Large VLDL
L−VLDL−TG
L−VLDL−PL
L−VLDL−P
L−VLDL−L
L−VLDL−FC
L−VLDL−CE
L−VLDL−C
Medium VLDL
M−VLDL−TG
M−VLDL−PL
M−VLDL−P
M−VLDL−L
M−VLDL−FC
M−VLDL−CE
M−VLDL−C
Small VLDL
S−VLDL−TG
S−VLDL−PL
S−VLDL−P
S−VLDL−L
S−VLDL−FC
S−VLDL−CE
S−VLDL−C
Very small VLDL
XS−VLDL−TG
XS−VLDL−PL
XS−VLDL−P
XS−VLDL−L
XS−VLDL−FC
XS−VLDL−CE
XS−VLDL−C
Large LDL
L−LDL−TG
L−LDL−PL
L−LDL−P
L−LDL−L
L−LDL−FC
L−LDL−CE
L−LDL−C
Medium LDL
M−LDL−TG
M−LDL−PL
M−LDL−P
M−LDL−L
M−LDL−FC
M−LDL−CE
M−LDL−C
Small LDL
S−LDL−TG
S−LDL−PL
S−LDL−P
S−LDL−L
S−LDL−FC
S−LDL−CE
S−LDL−C
IDL
IDL−TG
IDL−PL
IDL−P
IDL−L
IDL−FC
IDL−CE
IDL−C
Very large HDL
XL−HDL−TG
XL−HDL−PL
XL−HDL−P
XL−HDL−L
XL−HDL−FC
XL−HDL−CE
XL−HDL−C
Large HDL
L−HDL−TG
L−HDL−PL
L−HDL−P
L−HDL−L
L−HDL−FC
L−HDL−CE
L−HDL−C
Medium HDL
M−HDL−TG
M−HDL−PL
M−HDL−P
M−HDL−L
M−HDL−FC
M−HDL−CE
M−HDL−C
Small HDL
S−HDL−TG
S−HDL−PL
S−HDL−P
S−HDL−L
S−HDL−FC
S−HDL−CE
S−HDL−C

n

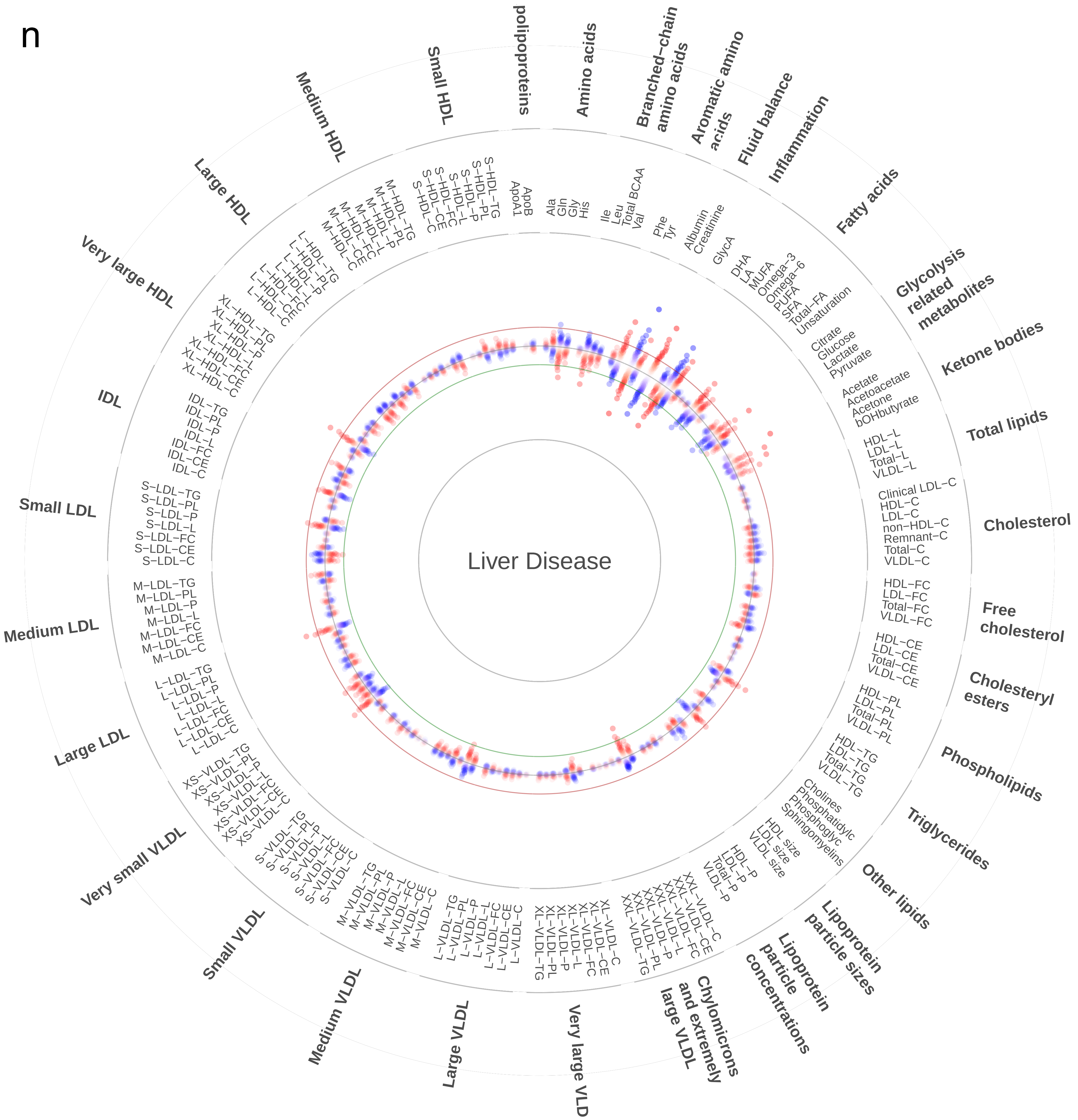
Liver Disease
polipoproteins
ApoB
ApoA1
Amino acids
Ala
Gln
Gly
His
Branched−chain amino acids
Ile
Leu
Total BCAA
Val
Aromatic amino acids
Phe
Tyr
Fluid balance
Albumin
Creatinine
Inflammation
GlycA
Fatty acids
DHA
LA
MUFA
Omega−3
Omega−6
PUFA
SFA
Total−FA
Unsaturation
Glycolysis related metabolites
Citrate
Glucose
Lactate
Pyruvate
Ketone bodies
Acetate
Acetoacetate
Acetone
bOHbutyrate
Total lipids
HDL−L
LDL−L
Total−L
VLDL−L
Cholesterol
Clinical LDL−C
HDL−C
LDL−C
non−HDL−C
Remnant−C
Total−C
VLDL−C
Free cholesterol
HDL−FC
LDL−FC
Total−FC
VLDL−FC
Cholesteryl esters
HDL−CE
LDL−CE
Total−CE
VLDL−CE
Phospholipids
HDL−PL
LDL−PL
Total−PL
VLDL−PL
Triglycerides
HDL−TG
LDL−TG
Total−TG
VLDL−TG
Other lipids
Cholines
Phosphatidylc
Phosphoglyc
Sphingomyelins
Lipoprotein particle sizes
HDL size
LDL size
VLDL size
Lipoprotein particle concentrations
HDL−P
LDL−P
Total−P
VLDL−P
Chylomicrons and extremely large VLDL
XXL−VLDL−TG
XXL−VLDL−PL
XXL−VLDL−P
XXL−VLDL−L
XXL−VLDL−FC
XXL−VLDL−CE
XXL−VLDL−C
Very large VLD
XL−VLDL−TG
XL−VLDL−PL
XL−VLDL−P
XL−VLDL−L
XL−VLDL−FC
XL−VLDL−CE
XL−VLDL−C
Large VLDL
L−VLDL−TG
L−VLDL−PL
L−VLDL−P
L−VLDL−L
L−VLDL−FC
L−VLDL−CE
L−VLDL−C
Medium VLDL
M−VLDL−TG
M−VLDL−PL
M−VLDL−P
M−VLDL−L
M−VLDL−FC
M−VLDL−CE
M−VLDL−C
Small VLDL
S−VLDL−TG
S−VLDL−PL
S−VLDL−P
S−VLDL−L
S−VLDL−FC
S−VLDL−CE
S−VLDL−C
Very small VLDL
XS−VLDL−TG
XS−VLDL−PL
XS−VLDL−P
XS−VLDL−L
XS−VLDL−FC
XS−VLDL−CE
XS−VLDL−C
Large LDL
L−LDL−TG
L−LDL−PL
L−LDL−P
L−LDL−L
L−LDL−FC
L−LDL−CE
L−LDL−C
Medium LDL
M−LDL−TG
M−LDL−PL
M−LDL−P
M−LDL−L
M−LDL−FC
M−LDL−CE
M−LDL−C
Small LDL
S−LDL−TG
S−LDL−PL
S−LDL−P
S−LDL−L
S−LDL−FC
S−LDL−CE
S−LDL−C
IDL
IDL−TG
IDL−PL
IDL−P
IDL−L
IDL−FC
IDL−CE
IDL−C
Very large HDL
XL−HDL−TG
XL−HDL−PL
XL−HDL−P
XL−HDL−L
XL−HDL−FC
XL−HDL−CE
XL−HDL−C
Large HDL
L−HDL−TG
L−HDL−PL
L−HDL−P
L−HDL−L
L−HDL−FC
L−HDL−CE
L−HDL−C
Medium HDL
M−HDL−TG
M−HDL−PL
M−HDL−P
M−HDL−L
M−HDL−FC
M−HDL−CE
M−HDL−C
Small HDL
S−HDL−TG
S−HDL−PL
S−HDL−P
S−HDL−L
S−HDL−FC
S−HDL−CE
S−HDL−C

O

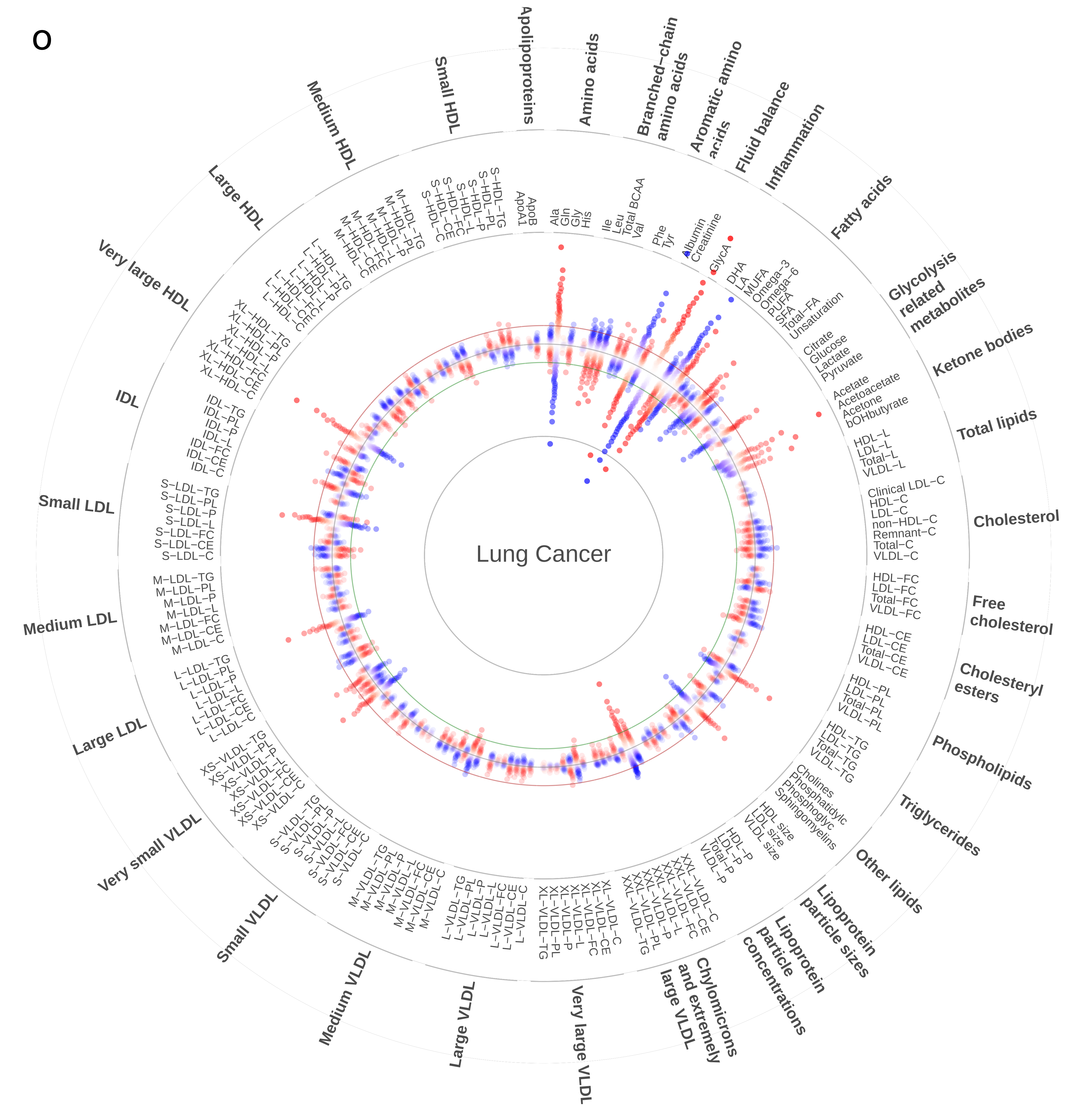
Lung Cancer
Apolipoproteins
Amino acids
Branched−chain amino acids
Aromatic amino acids
Fluid balance
Inflammation
Fatty acids
Glycolysis related metabolites
Ketone bodies
Total lipids
Cholesterol
Free cholesterol
Cholesteryl esters
Phospholipids
Triglycerides
Other lipids
Lipoprotein particle sizes
Lipoprotein particle concentrations
Chylomicrons and extremely large VLDL
Very large VLDL
Large VLDL
Medium VLDL
Small VLDL
Very small VLDL
Large LDL
Medium LDL
Small LDL
IDL
Very large HDL
Large HDL
Medium HDL
Small HDL
ApoB
ApoA1
Ala
Gln
Gly
His
Ile
Leu
Total BCAA
Val
Phe
Tyr
Albumin
Creatinine
GlycA
DHA
LA
MUFA
Omega−3
Omega−6
PUFA
SFA
Total−FA
Unsaturation
Citrate
Glucose
Lactate
Pyruvate
Acetate
Acetoacetate
Acetone
bOHbutyrate
HDL−L
LDL−L
Total−L
VLDL−L
Clinical LDL−C
HDL−C
LDL−C
non−HDL−C
Remnant−C
Total−C
VLDL−C
HDL−FC
LDL−FC
Total−FC
VLDL−FC
HDL−CE
LDL−CE
Total−CE
VLDL−CE
HDL−PL
LDL−PL
Total−PL
VLDL−PL
HDL−TG
LDL−TG
Total−TG
VLDL−TG
Cholines
Phosphatidylc
Phosphoglyc
Sphingomyelins
HDL size
LDL size
VLDL size
HDL−P
LDL−P
Total−P
VLDL−P
XXL−VLDL−C
XXL−VLDL−CE
XXL−VLDL−FC
XXL−VLDL−L
XXL−VLDL−P
XXL−VLDL−PL
XXL−VLDL−TG
XL−VLDL−C
XL−VLDL−CE
XL−VLDL−FC
XL−VLDL−L
XL−VLDL−P
XL−VLDL−PL
XL−VLDL−TG
L−VLDL−C
L−VLDL−CE
L−VLDL−FC
L−VLDL−L
L−VLDL−P
L−VLDL−PL
L−VLDL−TG
M−VLDL−C
M−VLDL−CE
M−VLDL−FC
M−VLDL−L
M−VLDL−P
M−VLDL−PL
M−VLDL−TG
S−VLDL−C
S−VLDL−CE
S−VLDL−FC
S−VLDL−L
S−VLDL−P
S−VLDL−PL
S−VLDL−TG
XS−VLDL−C
XS−VLDL−CE
XS−VLDL−FC
XS−VLDL−L
XS−VLDL−P
XS−VLDL−PL
XS−VLDL−TG
L−LDL−C
L−LDL−CE
L−LDL−FC
L−LDL−L
L−LDL−P
L−LDL−PL
L−LDL−TG
M−LDL−C
M−LDL−CE
M−LDL−FC
M−LDL−L
M−LDL−P
M−LDL−PL
M−LDL−TG
S−LDL−C
S−LDL−CE
S−LDL−FC
S−LDL−L
S−LDL−P
S−LDL−PL
S−LDL−TG
IDL−C
IDL−CE
IDL−FC
IDL−L
IDL−P
IDL−PL
IDL−TG
XL−HDL−C
XL−HDL−CE
XL−HDL−FC
XL−HDL−L
XL−HDL−P
XL−HDL−PL
XL−HDL−TG
L−HDL−C
L−HDL−CE
L−HDL−FC
L−HDL−L
L−HDL−P
L−HDL−PL
L−HDL−TG
M−HDL−C
M−HDL−CE
M−HDL−FC
M−HDL−L
M−HDL−P
M−HDL−PL
M−HDL−TG
S−HDL−C
S−HDL−CE
S−HDL−FC
S−HDL−L
S−HDL−P
S−HDL−PL
S−HDL−TG

p

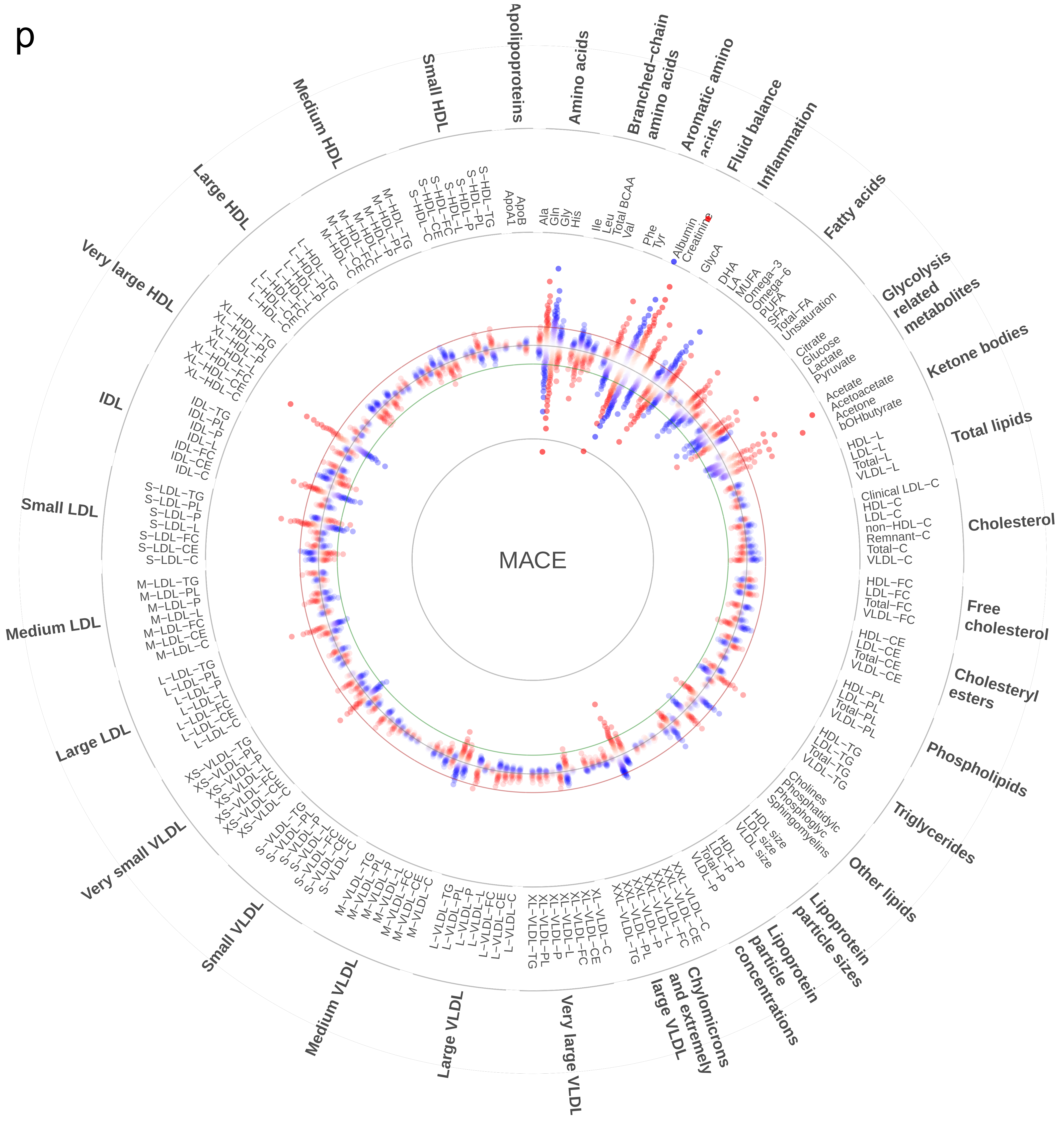

MACE
Apolipoproteins
Amino acids
Branched-chain amino acids
Aromatic amino acids
Fluid balance
Inflammation
Fatty acids
Glycolysis related metabolites
Ketone bodies
Total lipids
Cholesterol
Free cholesterol
Cholesteryl esters
Phospholipids
Triglycerides
Other lipids
Lipoprotein particle sizes
Lipoprotein particle concentrations
Chylomicrons and extremely large VLDL
Very large VLDL
Large VLDL
Medium VLDL
Small VLDL
Very small VLDL
Large LDL
Medium LDL
Small LDL
IDL
Very large HDL
Large HDL
Medium HDL
Small HDL
ApoB
ApoA1
Ala
Gln
Gly
His
Ile
Leu
Total BCAA
Val
Phe
Tyr
Albumin
Creatinine
GlycA
DHA
LA
MUFA
Omega-3
Omega-6
PUFA
SFA
Total-FA
Unsaturation
Citrate
Glucose
Lactate
Pyruvate
Acetate
Acetoacetate
Acetone
bOHbutyrate
HDL-L
LDL-L
Total-L
VLDL-L
Clinical LDL-C
HDL-C
LDL-C
non-HDL-C
Remnant-C
Total-C
VLDL-C
HDL-FC
LDL-FC
Total-FC
VLDL-FC
HDL-CE
LDL-CE
Total-CE
VLDL-CE
HDL-PL
LDL-PL
Total-PL
VLDL-PL
HDL-TG
LDL-TG
Total-TG
VLDL-TG
Cholines
Phosphatidylc
Phosphoglyc
Sphingomyelins
HDL size
LDL size
VLDL size
HDL-P
LDL-P
Total-P
VLDL-P
XXL-VLDL-C
XXL-VLDL-CE
XXL-VLDL-FC
XXL-VLDL-L
XXL-VLDL-P
XXL-VLDL-PL
XXL-VLDL-TG
XL-VLDL-C
XL-VLDL-CE
XL-VLDL-FC
XL-VLDL-L
XL-VLDL-P
XL-VLDL-PL
XL-VLDL-TG
L-VLDL-TG
L-VLDL-PL
L-VLDL-P
L-VLDL-L
L-VLDL-FC
L-VLDL-CE
L-VLDL-C
M-VLDL-TG
M-VLDL-PL
M-VLDL-P
M-VLDL-L
M-VLDL-FC
M-VLDL-CE
M-VLDL-C
S-VLDL-TG
S-VLDL-PL
S-VLDL-P
S-VLDL-L
S-VLDL-FC
S-VLDL-CE
S-VLDL-C
XS-VLDL-TG
XS-VLDL-PL
XS-VLDL-P
XS-VLDL-L
XS-VLDL-FC
XS-VLDL-CE
XS-VLDL-C
L-LDL-TG
L-LDL-PL
L-LDL-P
L-LDL-L
L-LDL-FC
L-LDL-CE
L-LDL-C
M-LDL-TG
M-LDL-PL
M-LDL-P
M-LDL-L
M-LDL-FC
M-LDL-CE
M-LDL-C
S-LDL-TG
S-LDL-PL
S-LDL-P
S-LDL-L
S-LDL-FC
S-LDL-CE
S-LDL-C
IDL-TG
IDL-PL
IDL-P
IDL-L
IDL-FC
IDL-CE
IDL-C
XL-HDL-TG
XL-HDL-PL
XL-HDL-P
XL-HDL-L
XL-HDL-FC
XL-HDL-CE
XL-HDL-C
L-HDL-TG
L-HDL-PL
L-HDL-P
L-HDL-L
L-HDL-FC
L-HDL-CE
L-HDL-C
M-HDL-TG
M-HDL-PL
M-HDL-P
M-HDL-L
M-HDL-FC
M-HDL-CE
M-HDL-C
S-HDL-TG
S-HDL-PL
S-HDL-P
S-HDL-L
S-HDL-FC
S-HDL-CE
S-HDL-C

q

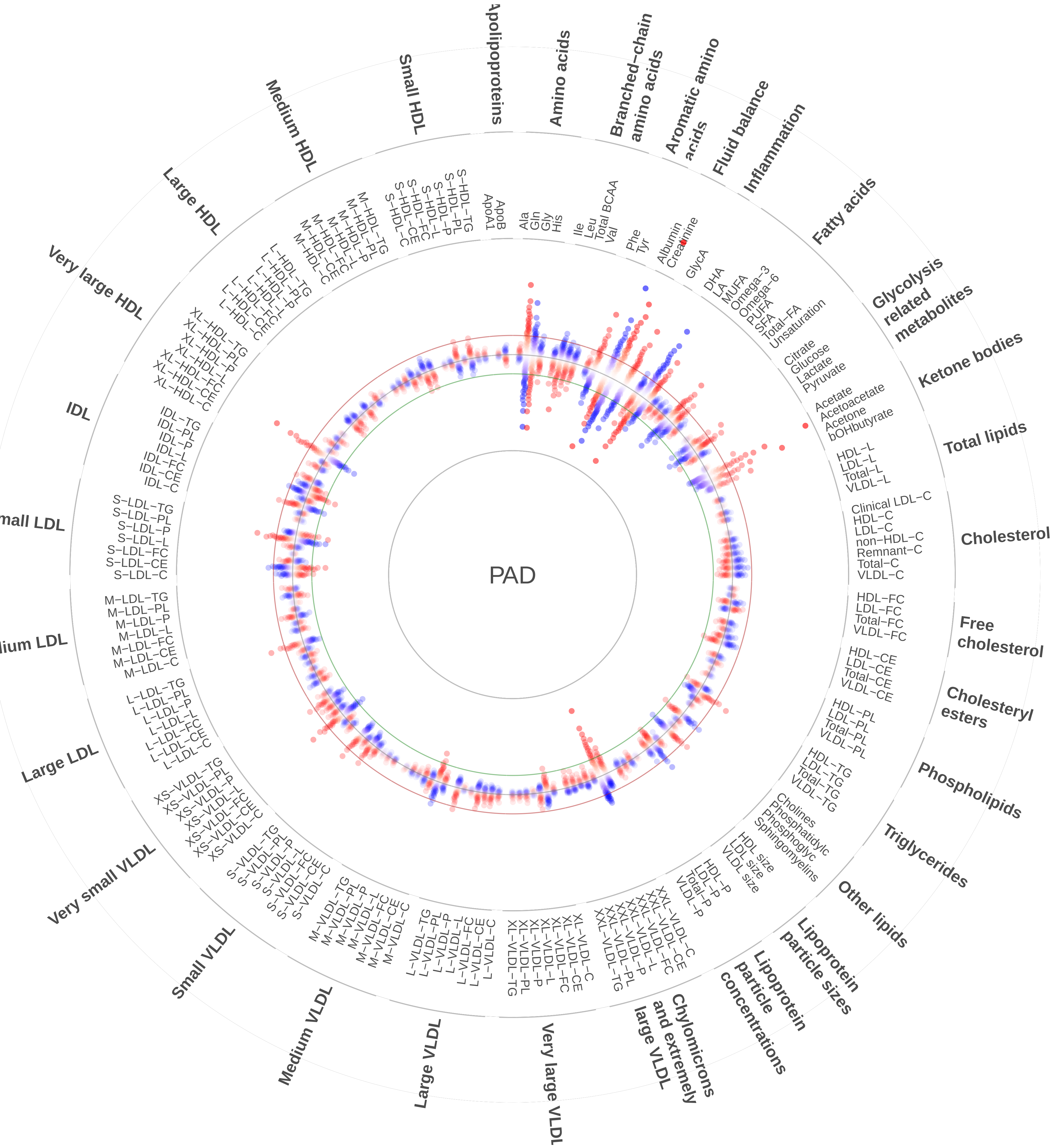

PAD
Apolipoproteins
Amino acids
Branched−chain amino acids
Aromatic amino acids
Fluid balance
Inflammation
Fatty acids
Glycolysis related metabolites
Ketone bodies
Total lipids
Cholesterol
Free cholesterol
Cholesteryl esters
Phospholipids
Triglycerides
Other lipids
Lipoprotein particle sizes
Lipoprotein particle concentrations
Chylomicrons and extremely large VLDL
Very large VLDL
Large VLDL
Medium VLDL
Small VLDL
Very small VLDL
Large LDL
Medium LDL
Small LDL
IDL
Very large HDL
Large HDL
Medium HDL
Small HDL
ApoB
ApoA1
Ala
Gln
Gly
His
Ile
Leu
Total BCAA
Val
Phe
Tyr
Albumin
Creatinine
GlycA
DHA
LA
MUFA
Omega−3
Omega−6
PUFA
SFA
Total−FA
Unsaturation
Citrate
Glucose
Lactate
Pyruvate
Acetate
Acetoacetate
Acetone
bOHbutyrate
HDL−L
LDL−L
Total−L
VLDL−L
Clinical LDL−C
HDL−C
LDL−C
non−HDL−C
Remnant−C
Total−C
VLDL−C
HDL−FC
LDL−FC
Total−FC
VLDL−FC
HDL−CE
LDL−CE
Total−CE
VLDL−CE
HDL−PL
LDL−PL
Total−PL
VLDL−PL
HDL−TG
LDL−TG
Total−TG
VLDL−TG
Cholines
Phosphatidylc
Phosphoglyc
Sphingomyelins
HDL size
LDL size
VLDL size
HDL−P
LDL−P
Total−P
VLDL−P
XXL−VLDL−C
XXL−VLDL−CE
XXL−VLDL−FC
XXL−VLDL−L
XXL−VLDL−P
XXL−VLDL−PL
XXL−VLDL−TG
XL−VLDL−C
XL−VLDL−CE
XL−VLDL−FC
XL−VLDL−L
XL−VLDL−P
XL−VLDL−PL
XL−VLDL−TG
L−VLDL−C
L−VLDL−CE
L−VLDL−FC
L−VLDL−L
L−VLDL−P
L−VLDL−PL
L−VLDL−TG
M−VLDL−C
M−VLDL−CE
M−VLDL−FC
M−VLDL−L
M−VLDL−P
M−VLDL−PL
M−VLDL−TG
S−VLDL−C
S−VLDL−CE
S−VLDL−FC
S−VLDL−L
S−VLDL−P
S−VLDL−PL
S−VLDL−TG
XS−VLDL−C
XS−VLDL−CE
XS−VLDL−FC
XS−VLDL−L
XS−VLDL−P
XS−VLDL−PL
XS−VLDL−TG
L−LDL−C
L−LDL−CE
L−LDL−FC
L−LDL−L
L−LDL−P
L−LDL−PL
L−LDL−TG
M−LDL−C
M−LDL−CE
M−LDL−FC
M−LDL−L
M−LDL−P
M−LDL−PL
M−LDL−TG
S−LDL−C
S−LDL−CE
S−LDL−FC
S−LDL−L
S−LDL−P
S−LDL−PL
S−LDL−TG
IDL−C
IDL−CE
IDL−FC
IDL−L
IDL−P
IDL−PL
IDL−TG
XL−HDL−C
XL−HDL−CE
XL−HDL−FC
XL−HDL−L
XL−HDL−P
XL−HDL−PL
XL−HDL−TG
L−HDL−C
L−HDL−CE
L−HDL−FC
L−HDL−L
L−HDL−P
L−HDL−PL
L−HDL−TG
M−HDL−C
M−HDL−CE
M−HDL−FC
M−HDL−L
M−HDL−P
M−HDL−PL
M−HDL−TG
S−HDL−C
S−HDL−CE
S−HDL−FC
S−HDL−L
S−HDL−P
S−HDL−PL
S−HDL−TG

r

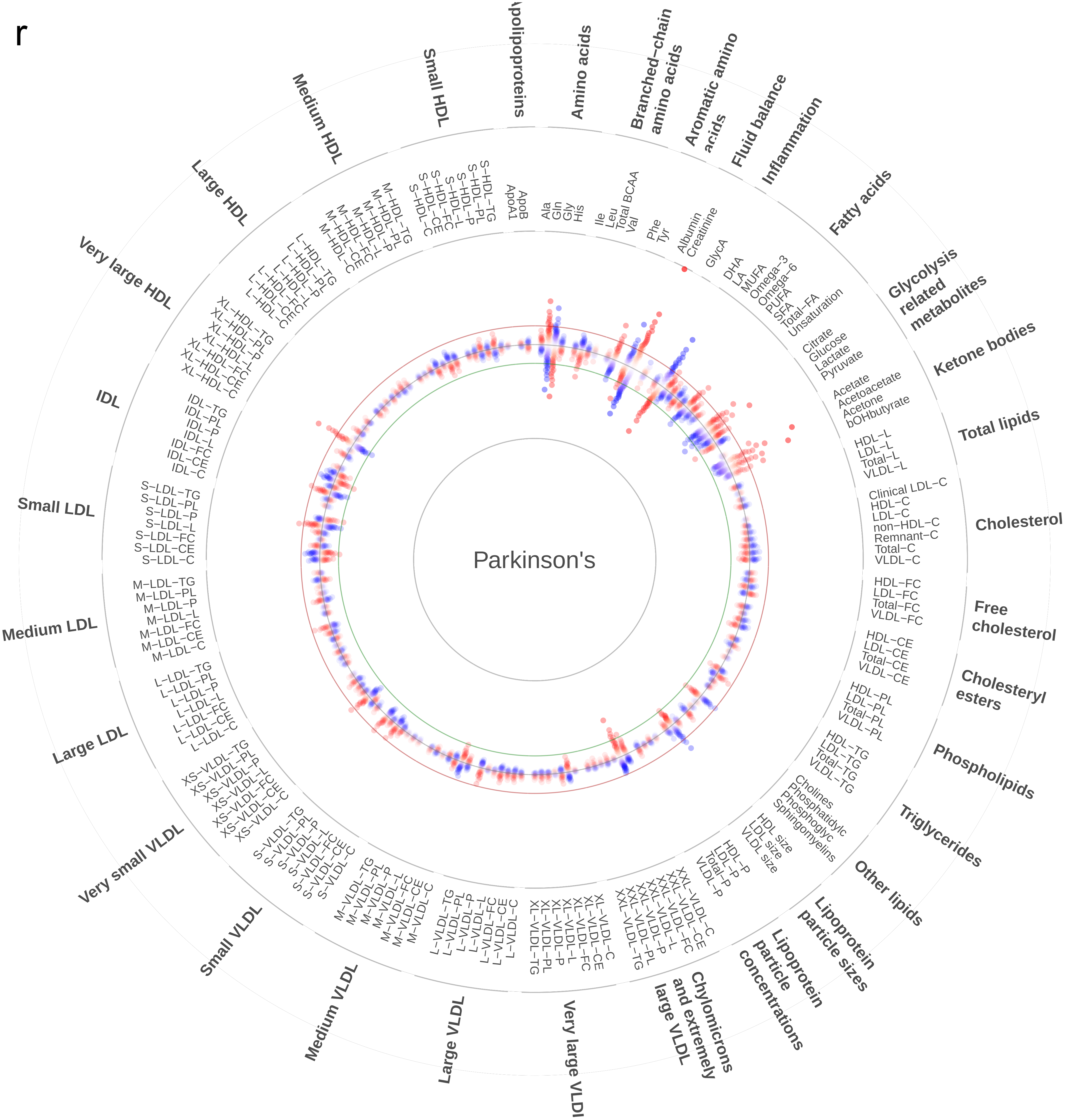
Parkinson's
Apolipoproteins
Amino acids
Branched−chain amino acids
Aromatic amino acids
Fluid balance
Inflammation
Fatty acids
Glycolysis related metabolites
Ketone bodies
Total lipids
Cholesterol
Free cholesterol
Cholesteryl esters
Phospholipids
Triglycerides
Other lipids
Lipoprotein particle sizes
Lipoprotein particle concentrations
Chylomicrons and extremely large VLDL
Very large VLDL
Large VLDL
Medium VLDL
Small VLDL
Very small VLDL
Large LDL
Medium LDL
Small LDL
IDL
Very large HDL
Large HDL
Medium HDL
Small HDL
ApoB
ApoA1
Ala
Gln
Gly
His
Ile
Leu
Total BCAA
Val
Phe
Tyr
Albumin
Creatinine
GlycA
DHA
LA
MUFA
Omega−3
Omega−6
PUFA
SFA
Total−FA
Unsaturation
Citrate
Glucose
Lactate
Pyruvate
Acetate
Acetoacetate
Acetone
bOHbutyrate
HDL−L
LDL−L
Total−L
VLDL−L
Clinical LDL−C
HDL−C
LDL−C
non−HDL−C
Remnant−C
Total−C
VLDL−C
HDL−FC
LDL−FC
Total−FC
VLDL−FC
HDL−CE
LDL−CE
Total−CE
VLDL−CE
HDL−PL
LDL−PL
Total−PL
VLDL−PL
HDL−TG
LDL−TG
Total−TG
VLDL−TG
Cholines
Phosphatidylc
Phosphoglyc
Sphingomyelins
HDL size
LDL size
VLDL size
HDL−P
LDL−P
Total−P
VLDL−P
XXL−VLDL−C
XXL−VLDL−CE
XXL−VLDL−FC
XXL−VLDL−L
XXL−VLDL−P
XXL−VLDL−PL
XXL−VLDL−TG
XL−VLDL−C
XL−VLDL−CE
XL−VLDL−FC
XL−VLDL−L
XL−VLDL−P
XL−VLDL−PL
XL−VLDL−TG
L−VLDL−C
L−VLDL−CE
L−VLDL−FC
L−VLDL−L
L−VLDL−P
L−VLDL−PL
L−VLDL−TG
M−VLDL−C
M−VLDL−CE
M−VLDL−FC
M−VLDL−L
M−VLDL−P
M−VLDL−PL
M−VLDL−TG
S−VLDL−C
S−VLDL−CE
S−VLDL−FC
S−VLDL−L
S−VLDL−P
S−VLDL−PL
S−VLDL−TG
XS−VLDL−C
XS−VLDL−CE
XS−VLDL−FC
XS−VLDL−L
XS−VLDL−P
XS−VLDL−PL
XS−VLDL−TG
L−LDL−C
L−LDL−CE
L−LDL−FC
L−LDL−L
L−LDL−P
L−LDL−PL
L−LDL−TG
M−LDL−C
M−LDL−CE
M−LDL−FC
M−LDL−L
M−LDL−P
M−LDL−PL
M−LDL−TG
S−LDL−C
S−LDL−CE
S−LDL−FC
S−LDL−L
S−LDL−P
S−LDL−PL
S−LDL−TG
IDL−C
IDL−CE
IDL−FC
IDL−L
IDL−P
IDL−PL
IDL−TG
XL−HDL−C
XL−HDL−CE
XL−HDL−FC
XL−HDL−L
XL−HDL−P
XL−HDL−PL
XL−HDL−TG
L−HDL−C
L−HDL−CE
L−HDL−FC
L−HDL−L
L−HDL−P
L−HDL−PL
L−HDL−TG
M−HDL−C
M−HDL−CE
M−HDL−FC
M−HDL−L
M−HDL−P
M−HDL−PL
M−HDL−TG
S−HDL−C
S−HDL−CE
S−HDL−FC
S−HDL−L
S−HDL−P
S−HDL−PL
S−HDL−TG

S

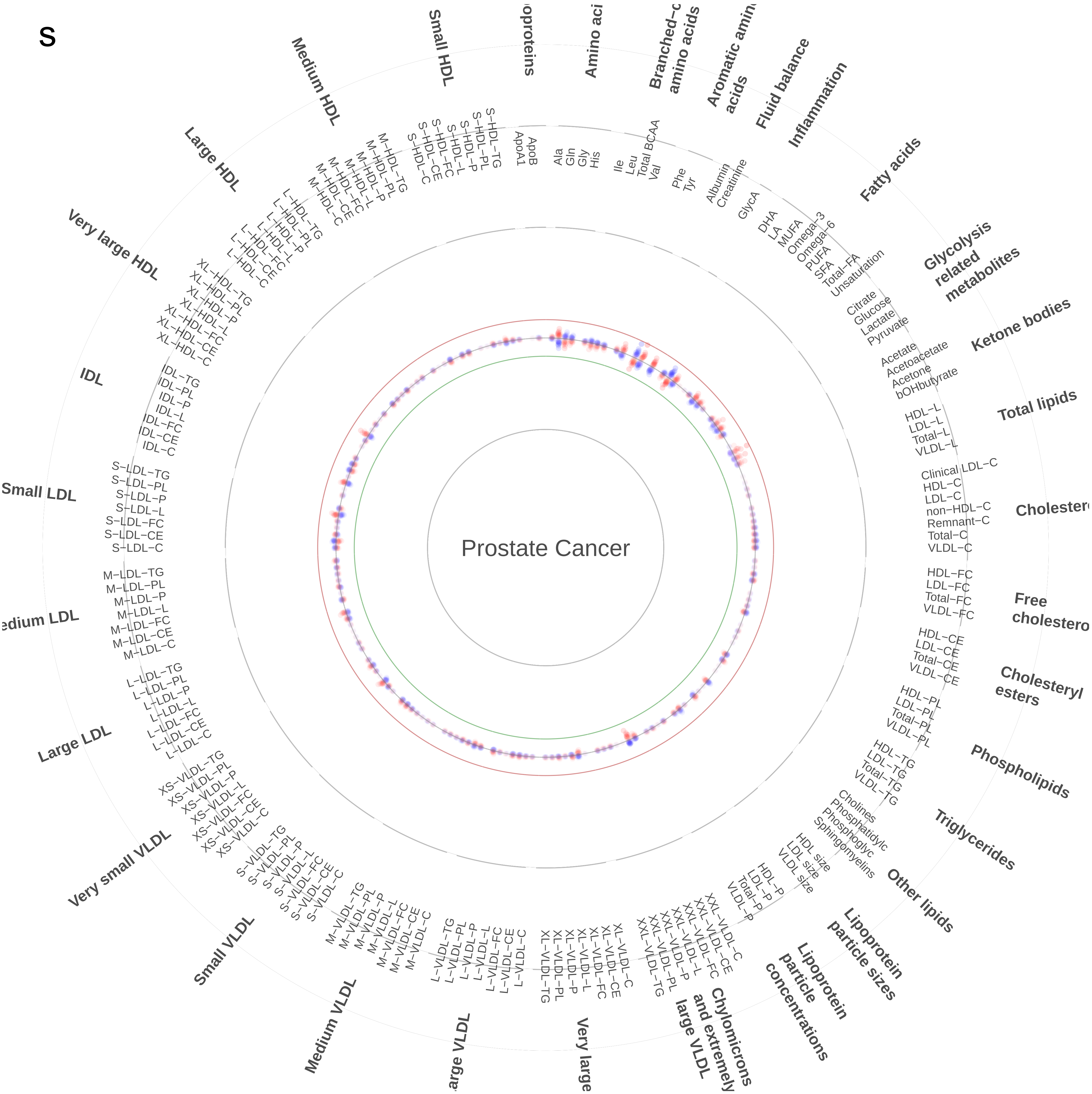

Prostate Cancer
Amino aci
Branched−c amino acids
Aromatic amin acids
Fluid balance
Inflammation
Fatty acids
Glycolysis related metabolites
Ketone bodies
Total lipids
Cholestero
Free cholestero
Cholesteryl esters
Phospholipids
Triglycerides
Other lipids
Lipoprotein particle sizes
Lipoprotein particle concentrations
Chylomicrons and extremely large VLDL
Very large
arge VLDL
Medium VLDL
Small VLDL
Very small VLDL
Large LDL
edium LDL
Small LDL
IDL
Very large HDL
Large HDL
Medium HDL
Small HDL
oproteins
ApoB
ApoA1
Ala
Gln
Gly
His
Ile
Leu
Total BCAA
Val
Phe
Tyr
Albumin
Creatinine
GlycA
DHA
LA
MUFA
Omega−3
Omega−6
PUFA
SFA
Total−FA
Unsaturation
Citrate
Glucose
Lactate
Pyruvate
Acetate
Acetoacetate
Acetone
bOHbutyrate
HDL−L
LDL−L
Total−L
VLDL−L
Clinical LDL−C
HDL−C
LDL−C
non−HDL−C
Remnant−C
Total−C
VLDL−C
HDL−FC
LDL−FC
Total−FC
VLDL−FC
HDL−CE
LDL−CE
Total−CE
VLDL−CE
HDL−PL
LDL−PL
Total−PL
VLDL−PL
HDL−TG
LDL−TG
Total−TG
VLDL−TG
Cholines
Phosphatidylc
Phosphoglyc
Sphingomyelins
HDL size
LDL size
VLDL size
HDL−P
LDL−P
Total−P
VLDL−P
XXL−VLDL−C
XXL−VLDL−CE
XXL−VLDL−FC
XXL−VLDL−L
XXL−VLDL−P
XXL−VLDL−PL
XXL−VLDL−TG
XL−VLDL−C
XL−VLDL−CE
XL−VLDL−FC
XL−VLDL−L
XL−VLDL−P
XL−VLDL−PL
XL−VLDL−TG
L−VLDL−C
L−VLDL−CE
L−VLDL−FC
L−VLDL−L
L−VLDL−P
L−VLDL−PL
L−VLDL−TG
M−VLDL−C
M−VLDL−CE
M−VLDL−FC
M−VLDL−L
M−VLDL−P
M−VLDL−PL
M−VLDL−TG
S−VLDL−C
S−VLDL−CE
S−VLDL−FC
S−VLDL−L
S−VLDL−P
S−VLDL−PL
S−VLDL−TG
XS−VLDL−C
XS−VLDL−CE
XS−VLDL−FC
XS−VLDL−L
XS−VLDL−P
XS−VLDL−PL
XS−VLDL−TG
L−LDL−C
L−LDL−CE
L−LDL−FC
L−LDL−L
L−LDL−P
L−LDL−PL
L−LDL−TG
M−LDL−C
M−LDL−CE
M−LDL−FC
M−LDL−L
M−LDL−P
M−LDL−PL
M−LDL−TG
S−LDL−C
S−LDL−CE
S−LDL−FC
S−LDL−L
S−LDL−P
S−LDL−PL
S−LDL−TG
IDL−C
IDL−CE
IDL−FC
IDL−L
IDL−P
IDL−PL
IDL−TG
XL−HDL−C
XL−HDL−CE
XL−HDL−FC
XL−HDL−L
XL−HDL−P
XL−HDL−PL
XL−HDL−TG
L−HDL−C
L−HDL−CE
L−HDL−FC
L−HDL−L
L−HDL−P
L−HDL−PL
L−HDL−TG
M−HDL−C
M−HDL−CE
M−HDL−FC
M−HDL−L
M−HDL−P
M−HDL−PL
M−HDL−TG
S−HDL−C
S−HDL−CE
S−HDL−FC
S−HDL−L
S−HDL−P
S−HDL−PL
S−HDL−TG

t

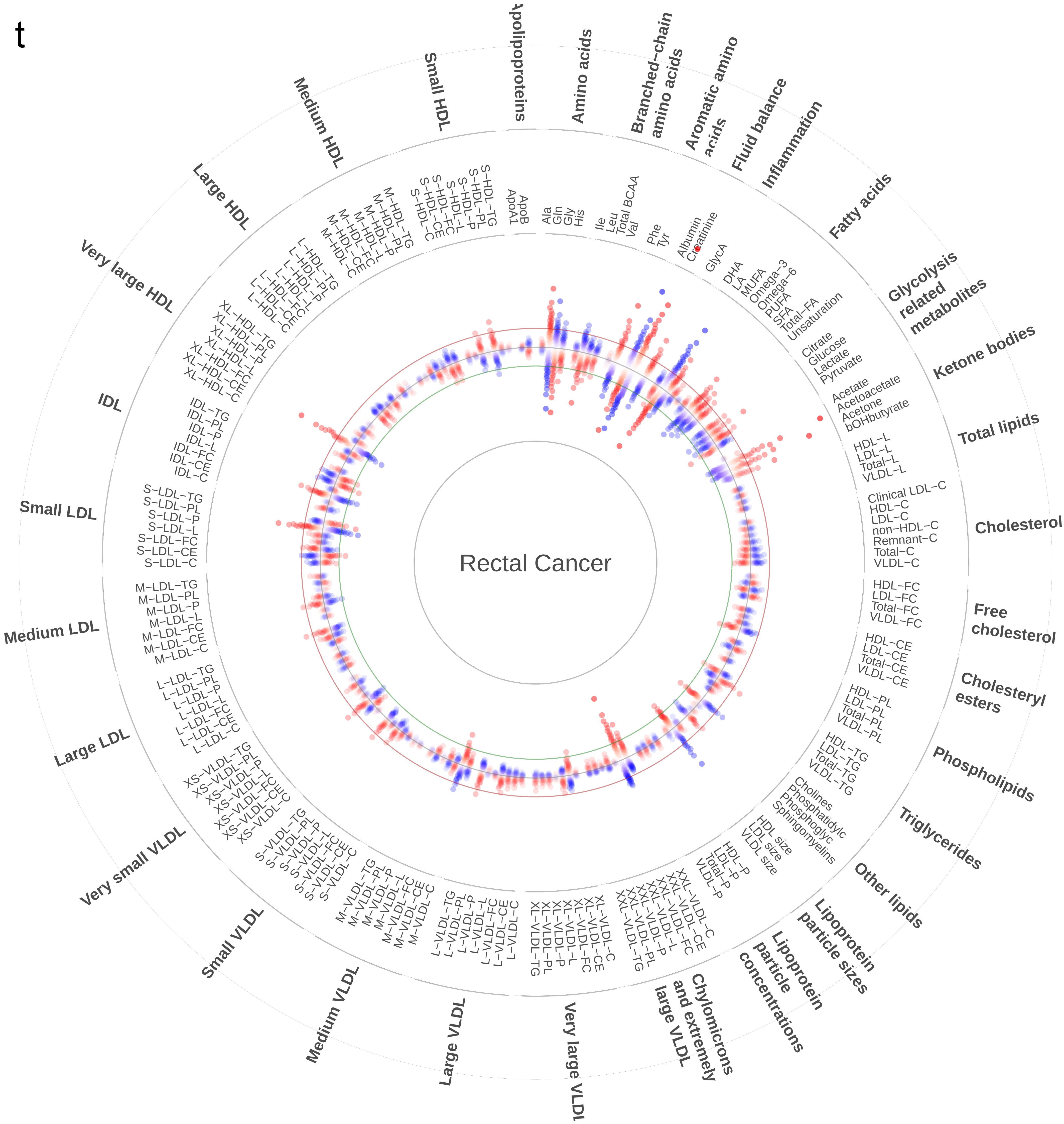

Rectal Cancer
Apolipoproteins
Amino acids
Branched−chain amino acids
Aromatic amino acids
Fluid balance
Inflammation
Fatty acids
Glycolysis related metabolites
Ketone bodies
Total lipids
Cholesterol
Free cholesterol
Cholesteryl esters
Phospholipids
Triglycerides
Other lipids
Lipoprotein particle sizes
Lipoprotein particle concentrations
Chylomicrons and extremely large VLDL
Very large VLDL
Large VLDL
Medium VLDL
Small VLDL
Very small VLDL
Large LDL
Medium LDL
Small LDL
IDL
Very large HDL
Large HDL
Medium HDL
Small HDL
ApoB
ApoA1
Ala
Gln
Gly
His
Ile
Leu
Total BCAA
Val
Phe
Tyr
Albumin
Creatinine
GlycA
DHA
LA
MUFA
Omega−3
Omega−6
PUFA
SFA
Total−FA
Unsaturation
Citrate
Glucose
Lactate
Pyruvate
Acetate
Acetoacetate
Acetone
bOHbutyrate
HDL−L
LDL−L
Total−L
VLDL−L
Clinical LDL−C
HDL−C
LDL−C
non−HDL−C
Remnant−C
Total−C
VLDL−C
HDL−FC
LDL−FC
Total−FC
VLDL−FC
HDL−CE
LDL−CE
Total−CE
VLDL−CE
HDL−PL
LDL−PL
Total−PL
VLDL−PL
HDL−TG
LDL−TG
Total−TG
VLDL−TG
Cholines
Phosphatidylc
Phosphoglyc
Sphingomyelins
HDL size
LDL size
VLDL size
HDL−P
LDL−P
Total−P
VLDL−P
XXL−VLDL−C
XXL−VLDL−CE
XXL−VLDL−FC
XXL−VLDL−L
XXL−VLDL−P
XXL−VLDL−PL
XXL−VLDL−TG
XL−VLDL−C
XL−VLDL−CE
XL−VLDL−FC
XL−VLDL−L
XL−VLDL−P
XL−VLDL−PL
XL−VLDL−TG
L−VLDL−C
L−VLDL−CE
L−VLDL−FC
L−VLDL−L
L−VLDL−P
L−VLDL−PL
L−VLDL−TG
M−VLDL−C
M−VLDL−CE
M−VLDL−FC
M−VLDL−L
M−VLDL−P
M−VLDL−PL
M−VLDL−TG
S−VLDL−C
S−VLDL−CE
S−VLDL−FC
S−VLDL−L
S−VLDL−P
S−VLDL−PL
S−VLDL−TG
XS−VLDL−C
XS−VLDL−CE
XS−VLDL−FC
XS−VLDL−L
XS−VLDL−P
XS−VLDL−PL
XS−VLDL−TG
L−LDL−C
L−LDL−CE
L−LDL−FC
L−LDL−L
L−LDL−P
L−LDL−PL
L−LDL−TG
M−LDL−C
M−LDL−CE
M−LDL−FC
M−LDL−L
M−LDL−P
M−LDL−PL
M−LDL−TG
S−LDL−C
S−LDL−CE
S−LDL−FC
S−LDL−L
S−LDL−P
S−LDL−PL
S−LDL−TG
IDL−C
IDL−CE
IDL−FC
IDL−L
IDL−P
IDL−PL
IDL−TG
XL−HDL−CE
XL−HDL−C
XL−HDL−FC
XL−HDL−L
XL−HDL−P
XL−HDL−PL
XL−HDL−TG
L−HDL−C
L−HDL−CE
L−HDL−FC
L−HDL−L
L−HDL−P
L−HDL−PL
L−HDL−TG
M−HDL−C
M−HDL−CE
M−HDL−FC
M−HDL−L
M−HDL−P
M−HDL−PL
M−HDL−TG
S−HDL−C
S−HDL−CE
S−HDL−FC
S−HDL−L
S−HDL−P
S−HDL−PL
S−HDL−TG

u

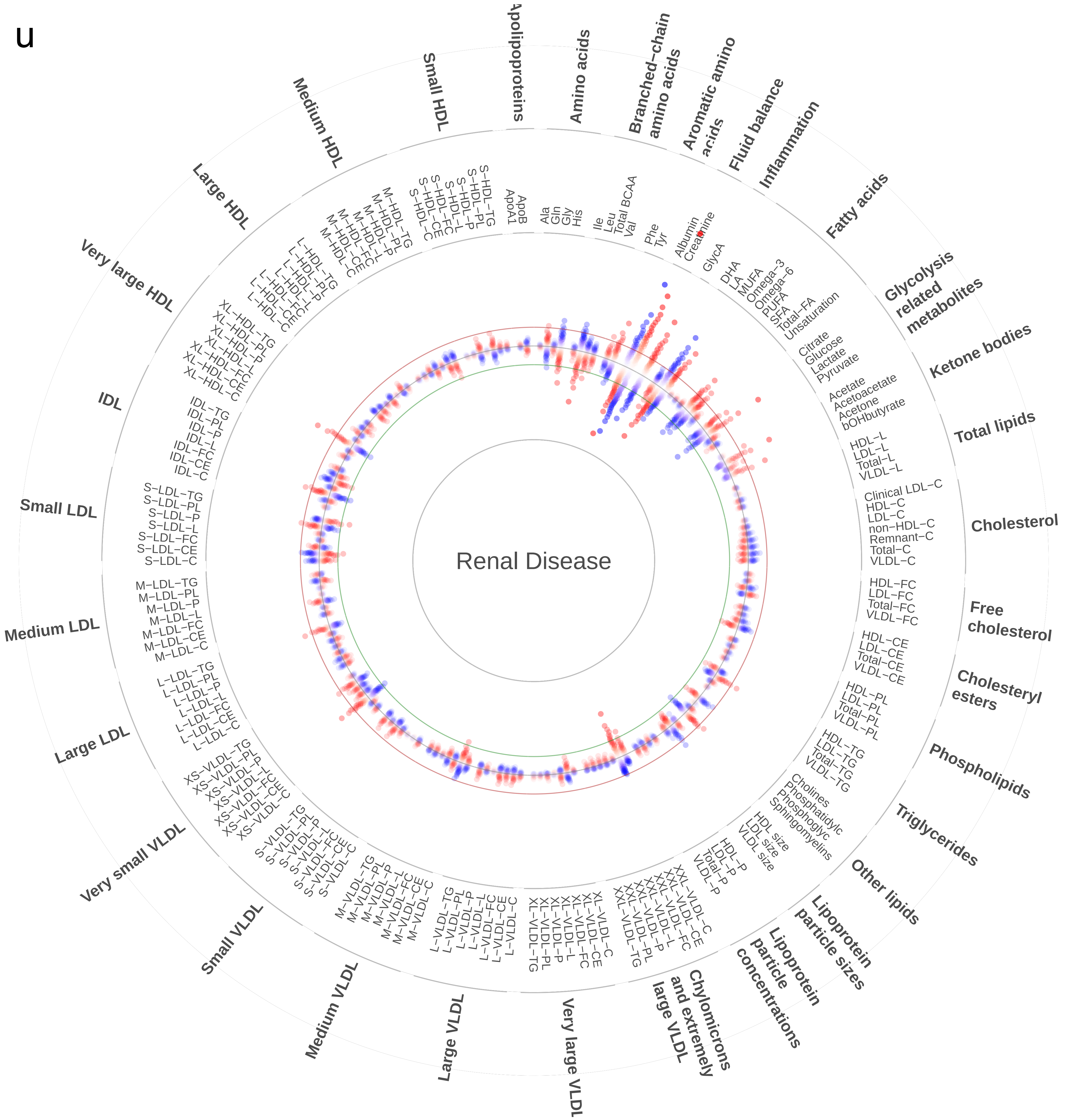

Renal Disease
Apolipoproteins
Amino acids
Branched−chain amino acids
Aromatic amino acids
Fluid balance
Inflammation
Fatty acids
Glycolysis related metabolites
Ketone bodies
Total lipids
Cholesterol
Free cholesterol
Cholesteryl esters
Phospholipids
Triglycerides
Other lipids
Lipoprotein particle sizes
Lipoprotein particle concentrations
Chylomicrons and extremely large VLDL
Very large VLDL
Large VLDL
Medium VLDL
Small VLDL
Very small VLDL
Large LDL
Medium LDL
Small LDL
IDL
Very large HDL
Large HDL
Medium HDL
Small HDL
ApoB
ApoA1
Ala
Gln
Gly
His
Ile
Leu
Total BCAA
Val
Phe
Tyr
Albumin
Creatinine
GlycA
DHA
LA
MUFA
Omega−3
Omega−6
PUFA
SFA
Total−FA
Unsaturation
Citrate
Glucose
Lactate
Pyruvate
Acetate
Acetoacetate
Acetone
bOHbutyrate
HDL−L
LDL−L
Total−L
VLDL−L
Clinical LDL−C
HDL−C
LDL−C
non−HDL−C
Remnant−C
Total−C
VLDL−C
HDL−FC
LDL−FC
Total−FC
VLDL−FC
HDL−CE
LDL−CE
Total−CE
VLDL−CE
HDL−PL
LDL−PL
Total−PL
VLDL−PL
HDL−TG
LDL−TG
Total−TG
VLDL−TG
Cholines
Phosphatidylc
Phosphoglyc
Sphingomyelins
HDL size
LDL size
VLDL size
HDL−P
LDL−P
Total−P
VLDL−P
XXL−VLDL−C
XXL−VLDL−CE
XXL−VLDL−FC
XXL−VLDL−L
XXL−VLDL−P
XXL−VLDL−PL
XXL−VLDL−TG
XL−VLDL−C
XL−VLDL−CE
XL−VLDL−FC
XL−VLDL−L
XL−VLDL−P
XL−VLDL−PL
XL−VLDL−TG
L−VLDL−C
L−VLDL−CE
L−VLDL−FC
L−VLDL−L
L−VLDL−P
L−VLDL−PL
L−VLDL−TG
M−VLDL−C
M−VLDL−CE
M−VLDL−FC
M−VLDL−L
M−VLDL−P
M−VLDL−PL
M−VLDL−TG
S−VLDL−C
S−VLDL−CE
S−VLDL−FC
S−VLDL−L
S−VLDL−P
S−VLDL−PL
S−VLDL−TG
XS−VLDL−C
XS−VLDL−CE
XS−VLDL−FC
XS−VLDL−L
XS−VLDL−P
XS−VLDL−PL
XS−VLDL−TG
L−LDL−C
L−LDL−CE
L−LDL−FC
L−LDL−L
L−LDL−P
L−LDL−PL
L−LDL−TG
M−LDL−C
M−LDL−CE
M−LDL−FC
M−LDL−L
M−LDL−P
M−LDL−PL
M−LDL−TG
S−LDL−C
S−LDL−CE
S−LDL−FC
S−LDL−L
S−LDL−P
S−LDL−PL
S−LDL−TG
IDL−C
IDL−CE
IDL−FC
IDL−L
IDL−P
IDL−PL
IDL−TG
XL−HDL−C
XL−HDL−CE
XL−HDL−FC
XL−HDL−L
XL−HDL−P
XL−HDL−PL
XL−HDL−TG
L−HDL−C
L−HDL−CE
L−HDL−FC
L−HDL−L
L−HDL−P
L−HDL−PL
L−HDL−TG
M−HDL−C
M−HDL−CE
M−HDL−FC
M−HDL−L
M−HDL−P
M−HDL−PL
M−HDL−TG
S−HDL−C
S−HDL−CE
S−HDL−FC
S−HDL−L
S−HDL−P
S−HDL−PL
S−HDL−TG

V

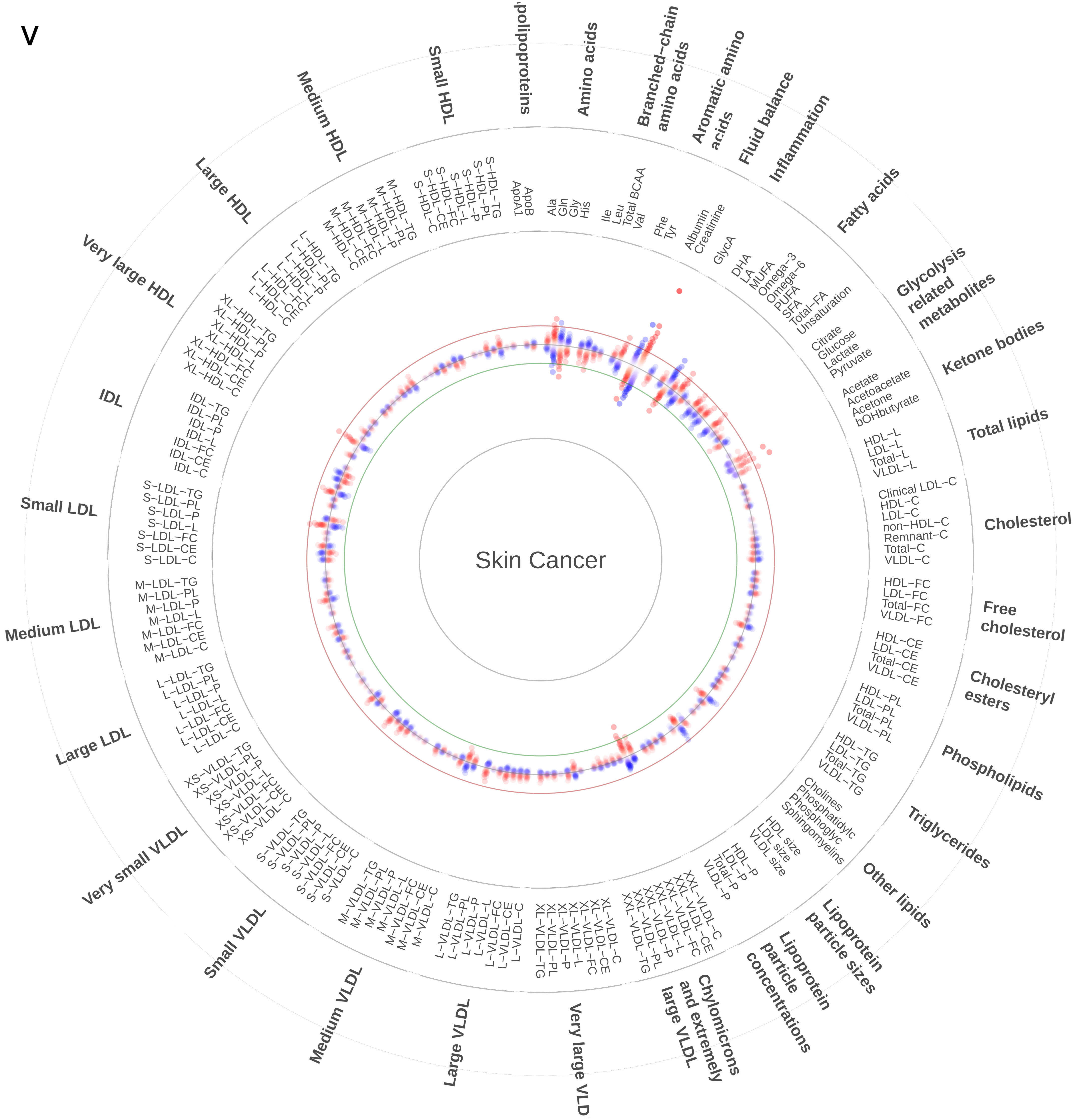

Skin Cancer
Amino acids
Branched−chain amino acids
Aromatic amino acids
Fluid balance
Inflammation
Fatty acids
Glycolysis related metabolites
Ketone bodies
Total lipids
Cholesterol
Free cholesterol
Cholesteryl esters
Phospholipids
Triglycerides
Other lipids
Lipoprotein particle sizes
Lipoprotein particle concentrations
Chylomicrons and extremely large VLDL
Very large VLDL
Large VLDL
Medium VLDL
Small VLDL
Very small VLDL
Large LDL
Medium LDL
Small LDL
IDL
Very large HDL
Large HDL
Medium HDL
Small HDL
Apolipoproteins
Ala
Gln
Gly
His
Ile
Leu
Total BCAA
Val
Phe
Tyr
Albumin
Creatinine
GlycA
DHA
LA
MUFA
Omega−3
Omega−6
PUFA
SFA
Total−FA
Unsaturation
Citrate
Glucose
Lactate
Pyruvate
Acetate
Acetoacetate
Acetone
bOHbutyrate
HDL−L
LDL−L
Total−L
VLDL−L
Clinical LDL−C
HDL−C
LDL−C
non−HDL−C
Remnant−C
Total−C
VLDL−C
HDL−FC
LDL−FC
Total−FC
VLDL−FC
HDL−CE
LDL−CE
Total−CE
VLDL−CE
HDL−PL
LDL−PL
Total−PL
VLDL−PL
HDL−TG
LDL−TG
Total−TG
VLDL−TG
Cholines
Phosphatidylc
Phosphoglyc
Sphingomyelins
HDL size
LDL size
VLDL size
HDL−P
LDL−P
Total−P
VLDL−P
XXL−VLDL−C
XXL−VLDL−CE
XXL−VLDL−FC
XXL−VLDL−L
XXL−VLDL−P
XXL−VLDL−PL
XXL−VLDL−TG
XL−VLDL−C
XL−VLDL−CE
XL−VLDL−FC
XL−VLDL−L
XL−VLDL−P
XL−VLDL−PL
XL−VLDL−TG
L−VLDL−C
L−VLDL−CE
L−VLDL−FC
L−VLDL−L
L−VLDL−P
L−VLDL−PL
L−VLDL−TG
M−VLDL−C
M−VLDL−CE
M−VLDL−FC
M−VLDL−L
M−VLDL−P
M−VLDL−PL
M−VLDL−TG
S−VLDL−C
S−VLDL−CE
S−VLDL−FC
S−VLDL−L
S−VLDL−P
S−VLDL−PL
S−VLDL−TG
XS−VLDL−C
XS−VLDL−CE
XS−VLDL−FC
XS−VLDL−L
XS−VLDL−P
XS−VLDL−PL
XS−VLDL−TG
L−LDL−C
L−LDL−CE
L−LDL−FC
L−LDL−L
L−LDL−P
L−LDL−PL
L−LDL−TG
M−LDL−C
M−LDL−CE
M−LDL−FC
M−LDL−L
M−LDL−P
M−LDL−PL
M−LDL−TG
S−LDL−C
S−LDL−CE
S−LDL−FC
S−LDL−L
S−LDL−P
S−LDL−PL
S−LDL−TG
IDL−C
IDL−CE
IDL−FC
IDL−L
IDL−P
IDL−PL
IDL−TG
XL−HDL−C
XL−HDL−CE
XL−HDL−FC
XL−HDL−L
XL−HDL−P
XL−HDL−PL
XL−HDL−TG
L−HDL−C
L−HDL−CE
L−HDL−FC
L−HDL−L
L−HDL−P
L−HDL−PL
L−HDL−TG
M−HDL−C
M−HDL−CE
M−HDL−FC
M−HDL−L
M−HDL−P
M−HDL−PL
M−HDL−TG
S−HDL−C
S−HDL−CE
S−HDL−FC
S−HDL−L
S−HDL−P
S−HDL−PL
S−HDL−TG
ApoB
ApoA1

W

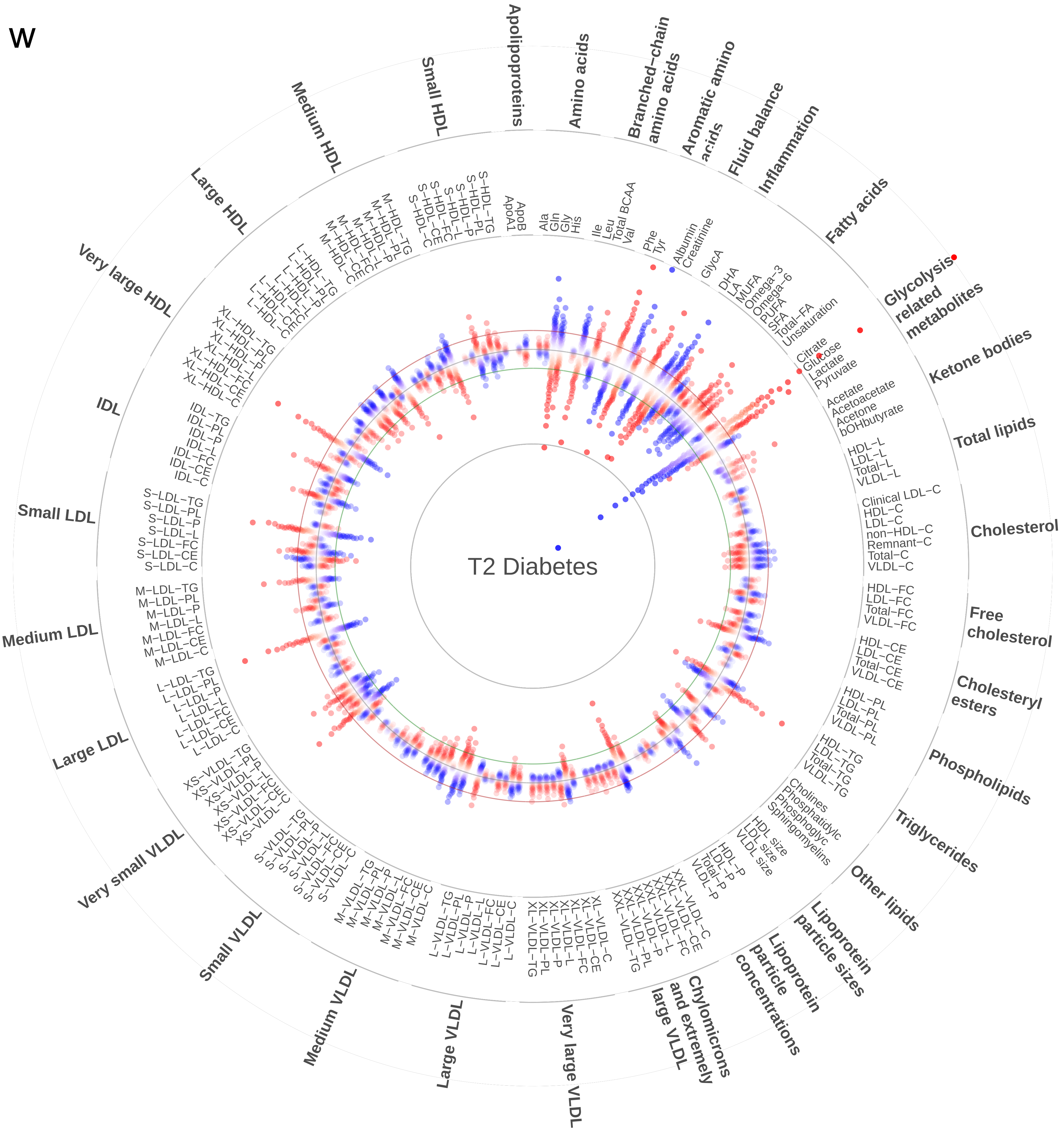

T2 Diabetes
Apolipoproteins
Amino acids
Branched–chain amino acids
Aromatic amino acids
Fluid balance
Inflammation
Fatty acids
Glycolysis related metabolites
Ketone bodies
Total lipids
Cholesterol
Free cholesterol
Cholesteryl esters
Phospholipids
Triglycerides
Other lipids
Lipoprotein particle sizes
Lipoprotein particle concentrations
Chylomicrons and extremely large VLDL
Very large VLDL
Large VLDL
Medium VLDL
Small VLDL
Very small VLDL
Large LDL
Medium LDL
Small LDL
IDL
Very large HDL
Large HDL
Medium HDL
Small HDL
ApoB
ApoA1
Ala
Gln
Gly
His
Ile
Leu
Total BCAA
Val
Phe
Tyr
Albumin
Creatinine
GlycA
DHA
LA
MUFA
Omega–3
Omega–6
PUFA
SFA
Total–FA
Unsaturation
Citrate
Glucose
Lactate
Pyruvate
Acetate
Acetoacetate
Acetone
bOHbutyrate
HDL–L
LDL–L
Total–L
VLDL–L
Clinical LDL–C
HDL–C
LDL–C
non–HDL–C
Remnant–C
Total–C
VLDL–C
HDL–FC
LDL–FC
Total–FC
VLDL–FC
HDL–CE
LDL–CE
Total–CE
VLDL–CE
HDL–PL
LDL–PL
Total–PL
VLDL–PL
HDL–TG
LDL–TG
Total–TG
VLDL–TG
Cholines
Phosphatidylc
Phosphoglyc
Sphingomyelins
HDL size
LDL size
VLDL size
HDL–P
LDL–P
Total–P
VLDL–P
XXL–VLDL–C
XXL–VLDL–CE
XXL–VLDL–FC
XXL–VLDL–L
XXL–VLDL–P
XXL–VLDL–PL
XXL–VLDL–TG
XL–VLDL–C
XL–VLDL–CE
XL–VLDL–FC
XL–VLDL–L
XL–VLDL–P
XL–VLDL–PL
XL–VLDL–TG
L–VLDL–C
L–VLDL–CE
L–VLDL–FC
L–VLDL–L
L–VLDL–P
L–VLDL–PL
L–VLDL–TG
M–VLDL–C
M–VLDL–CE
M–VLDL–FC
M–VLDL–L
M–VLDL–P
M–VLDL–PL
M–VLDL–TG
S–VLDL–C
S–VLDL–CE
S–VLDL–FC
S–VLDL–L
S–VLDL–P
S–VLDL–PL
S–VLDL–TG
XS–VLDL–C
XS–VLDL–CE
XS–VLDL–FC
XS–VLDL–L
XS–VLDL–P
XS–VLDL–PL
XS–VLDL–TG
L–LDL–C
L–LDL–CE
L–LDL–FC
L–LDL–L
L–LDL–P
L–LDL–PL
L–LDL–TG
M–LDL–C
M–LDL–CE
M–LDL–FC
M–LDL–L
M–LDL–P
M–LDL–PL
M–LDL–TG
S–LDL–C
S–LDL–CE
S–LDL–FC
S–LDL–L
S–LDL–P
S–LDL–PL
S–LDL–TG
IDL–C
IDL–CE
IDL–FC
IDL–L
IDL–P
IDL–PL
IDL–TG
XL–HDL–CE
XL–HDL–C
XL–HDL–FC
XL–HDL–L
XL–HDL–P
XL–HDL–PL
XL–HDL–TG
L–HDL–C
L–HDL–CE
L–HDL–FC
L–HDL–L
L–HDL–P
L–HDL–PL
L–HDL–TG
M–HDL–C
M–HDL–CE
M–HDL–FC
M–HDL–L
M–HDL–P
M–HDL–PL
M–HDL–TG
S–HDL–C
S–HDL–CE
S–HDL–FC
S–HDL–L
S–HDL–P
S–HDL–PL
S–HDL–TG

X

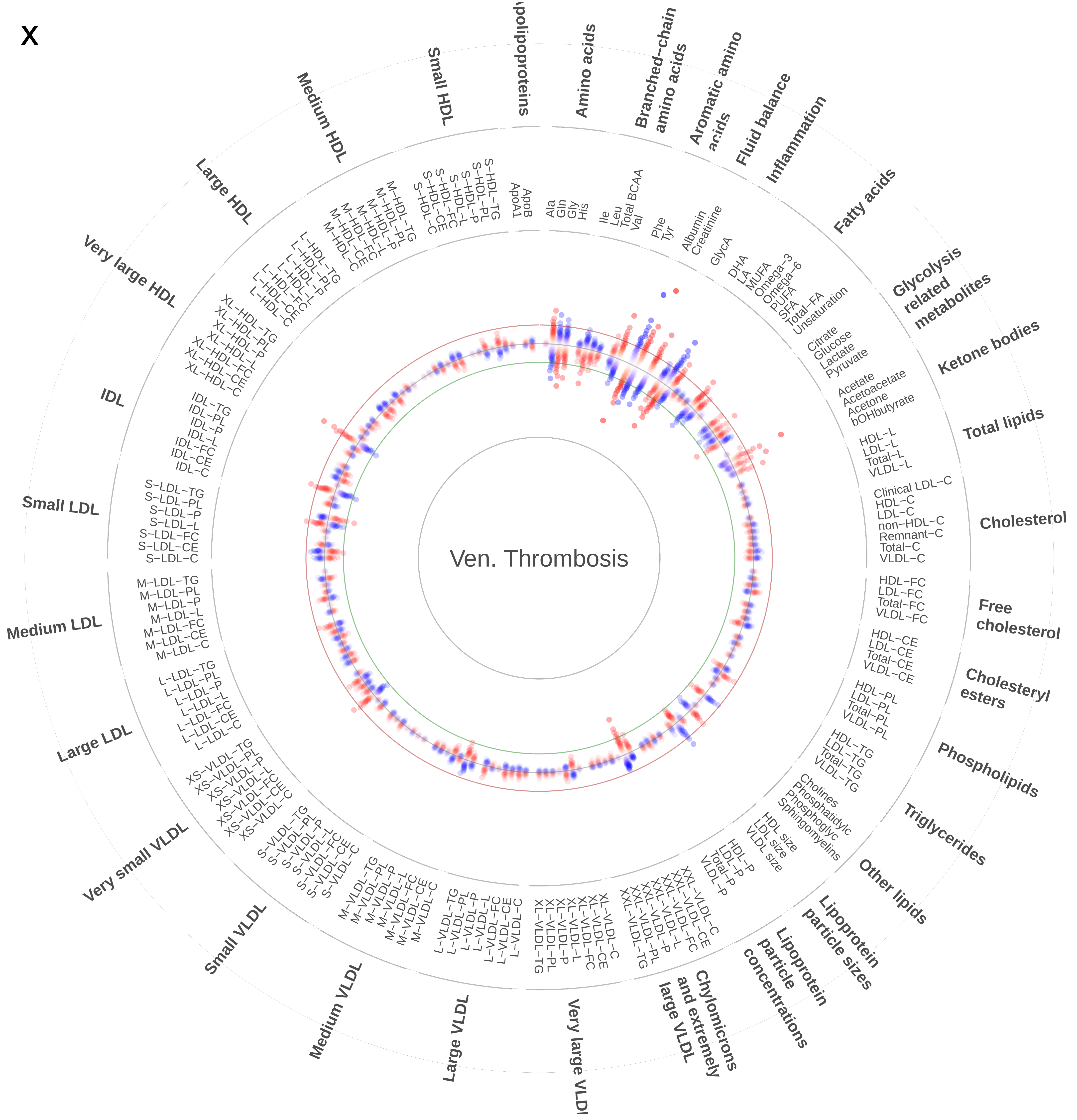

Ven. Thrombosis
Amino acids
Branched−chain amino acids
Aromatic amino acids
Fluid balance
Inflammation
Fatty acids
Glycolysis related metabolites
Ketone bodies
Total lipids
Cholesterol
Free cholesterol
Cholesteryl esters
Phospholipids
Triglycerides
Other lipids
Lipoprotein particle sizes
Lipoprotein particle concentrations
Chylomicrons and extremely large VLDL
Large VLDL
Medium VLDL
Small VLDL
Very small VLDL
Large LDL
Medium LDL
Small LDL
IDL
Very large HDL
Large HDL
Medium HDL
Small HDL
ApoB
ApoA1
Ala
Gln
Gly
His
Ile
Leu
Total BCAA
Val
Phe
Tyr
Albumin
Creatinine
GlycA
DHA
LA
MUFA
Omega−3
Omega−6
PUFA
SFA
Total−FA
Unsaturation
Citrate
Glucose
Lactate
Pyruvate
Acetate
Acetoacetate
Acetone
bOHbutyrate
HDL−L
LDL−L
Total−L
VLDL−L
Clinical LDL−C
HDL−C
LDL−C
non−HDL−C
Remnant−C
Total−C
VLDL−C
HDL−FC
LDL−FC
Total−FC
VLDL−FC
HDL−CE
LDL−CE
Total−CE
VLDL−CE
HDL−PL
LDL−PL
Total−PL
VLDL−PL
HDL−TG
LDL−TG
Total−TG
VLDL−TG
Cholines
Phosphatidylc
Phosphoglyc
Sphingomyelins
HDL size
LDL size
VLDL size
HDL−P
LDL−P
Total−P
VLDL−P
XXL−VLDL−C
XXL−VLDL−CE
XXL−VLDL−FC
XXL−VLDL−L
XXL−VLDL−P
XXL−VLDL−PL
XXL−VLDL−TG
XL−VLDL−C
XL−VLDL−CE
XL−VLDL−FC
XL−VLDL−L
XL−VLDL−P
XL−VLDL−PL
XL−VLDL−TG
L−VLDL−TG
L−VLDL−PL
L−VLDL−P
L−VLDL−L
L−VLDL−FC
L−VLDL−CE
L−VLDL−C
M−VLDL−TG
M−VLDL−PL
M−VLDL−P
M−VLDL−L
M−VLDL−FC
M−VLDL−CE
M−VLDL−C
S−VLDL−TG
S−VLDL−PL
S−VLDL−P
S−VLDL−L
S−VLDL−FC
S−VLDL−CE
S−VLDL−C
XS−VLDL−TG
XS−VLDL−PL
XS−VLDL−P
XS−VLDL−L
XS−VLDL−FC
XS−VLDL−CE
XS−VLDL−C
L−LDL−TG
L−LDL−PL
L−LDL−P
L−LDL−L
L−LDL−FC
L−LDL−CE
L−LDL−C
M−LDL−TG
M−LDL−PL
M−LDL−P
M−LDL−L
M−LDL−FC
M−LDL−CE
M−LDL−C
S−LDL−TG
S−LDL−PL
S−LDL−P
S−LDL−L
S−LDL−FC
S−LDL−CE
S−LDL−C
IDL−TG
IDL−PL
IDL−P
IDL−L
IDL−FC
IDL−CE
IDL−C
XL−HDL−TG
XL−HDL−PL
XL−HDL−P
XL−HDL−L
XL−HDL−FC
XL−HDL−CE
XL−HDL−C
L−HDL−TG
L−HDL−PL
L−HDL−P
L−HDL−L
L−HDL−FC
L−HDL−CE
L−HDL−C
M−HDL−TG
M−HDL−PL
M−HDL−P
M−HDL−L
M−HDL−FC
M−HDL−CE
M−HDL−C
S−HDL−TG
S−HDL−PL
S−HDL−P
S−HDL−L
S−HDL−FC
S−HDL−CE
S−HDL−C
