# Supplementary material for: Metabolomic profiles predict individual multidisease outcomes
Source: Nat Med. 2022 Sep 22;28(11):2309–20. doi: 10.1038/s41591-022-01980-3 (PMC9671812; doi:10.1038/s41591-022-01980-3)
Supplement: Supplementary file 2 — Reporting Summary [file 41591_2022_1980_MOESM2_ESM.pdf]

nature portfolio

Corresponding author(s): Roland Eils

Last updated by author(s): Jul 13, 2022

Reporting Summary

Nature Portfolio wishes to improve the reproducibility of the work that we publish. This form provides structure for consistency and transparency in reporting. For further information on Nature Portfolio policies, see our Editorial Policies and the Editorial Policy Checklist.

Statistics

For all statistical analyses, confirm that the following items are present in the figure legend, table legend, main text, or Methods section.

n/a	Confirmed	
☐	☒	The exact sample size (n) for each experimental group/condition, given as a discrete number and unit of measurement
☐	☒	A statement on whether measurements were taken from distinct samples or whether the same sample was measured repeatedly
☐	☒	The statistical test(s) used AND whether they are one- or two-sided<br>*Only common tests should be described solely by name; describe more complex techniques in the Methods section.*
☐	☒	A description of all covariates tested
☐	☒	A description of any assumptions or corrections, such as tests of normality and adjustment for multiple comparisons
☐	☒	A full description of the statistical parameters including central tendency (e.g. means) or other basic estimates (e.g. regression coefficient) AND variation (e.g. standard deviation) or associated estimates of uncertainty (e.g. confidence intervals)
☒	☐	For null hypothesis testing, the test statistic (e.g. F, t, r) with confidence intervals, effect sizes, degrees of freedom and P value noted<br>*Give P values as exact values whenever suitable.*
☒	☐	For Bayesian analysis, information on the choice of priors and Markov chain Monte Carlo settings
☒	☐	For hierarchical and complex designs, identification of the appropriate level for tests and full reporting of outcomes
☒	☐	Estimates of effect sizes (e.g. Cohen's d, Pearson's r), indicating how they were calculated

Our web collection on statistics for biologists contains articles on many of the points above.

Software and code

Policy information about availability of computer code

Data collection	No software was used for data collection. This study relied on data collected for the UK Biobank Cohort, the Whiltehall II Cohort and three cohorts from the BBMRI-NL consortium as stated in the manuscript.
Data analysis	Machine learning analysis was conducted in Python 3.7 with PyTorch 1.9.0, PyTorch Lightning 1.3.8 and lifelines 0.26.0, Statistical analysis and figures were produced in RStudio 4.0.2 (RStudio Inc., Boston, MA), as stated in the manuscript. All code developed and used throughout this study has been made open source and is available on Github. The code to train the metabolomic state model can be found here: github.com/thbuerg/MetabolomicsCommonDiseases while the code to run analysis on trained models can be found here: github.com/JakobSteinfeldt/MetabolomicsCommonDiseases.

For manuscripts utilizing custom algorithms or software that are central to the research but not yet described in published literature, software must be made available to editors and reviewers. We strongly encourage code deposition in a community repository (e.g. GitHub). See the Nature Portfolio guidelines for submitting code & software for further information.

Data

Policy information about availability of data

All manuscripts must include a data availability statement. This statement should provide the following information, where applicable:

- Accession codes, unique identifiers, or web links for publicly available datasets
- A description of any restrictions on data availability
- For clinical datasets or third party data, please ensure that the statement adheres to our policy

UK Biobank data, including NMR metabolomics, are publicly available to bona fide researchers upon application at http://www.ukbiobank.ac.uk/using-the-

resource/. Detailed information on predictors and endpoints used in this study are presented in Supplementary Tables 1, 2, and 3. The Whitehall II data are available for the scientific community. Researchers are invited to apply for data access at https://www.dementiasplatform.uk/. Data of the BBMRI-NL consortium is available upon application at https://www.bbmri.nl/Omics-metabolomics.

Field-specific reporting

Please select the one below that is the best fit for your research. If you are not sure, read the appropriate sections before making your selection.

☒ Life sciences ☐ Behavioural & social sciences ☐ Ecological, evolutionary & environmental sciences

For a reference copy of the document with all sections, see nature.com/documents/nr-reporting-summary-flat.pdf

Life sciences study design

All studies must disclose on these points even when the disclosure is negative.

Sample size	This study relied on data from the UK Biobank cohort. All 117,981 participants with available NMR metabolomics at baseline were included in this study. For validation data from 11,684 participants from four independent cohorts, the Whitehall II cohort (N=6117) and three BBRMI-NL cohorts, the Leiden Longevity Study (N=1655), the PROSPER cohort (N=960) and the Rotterdam study (N=2949) were utilized. As this study utilized all available data points, no specific procedure to determine the sample size was applied a priori. Sample size was considered sufficient, as the total sum of participants was larger than or in the range of similar studies in the scientific literature, see https://www.nature.com/articles/s41467-019-11311-9 and https://elifesciences.org/articles/63033.
Data exclusions	No data was excluded from the analysis.
Replication	Development and validation of machine learning models was performed over 22 spatially separated hold-out validation sets. Specifically, data was split by the location of the UK Biobank assessment center of the participants, resulting in 22 spatially separated sets. For each assessment center, the corresponding set was considered the test set, while the remaining 21 sets, were pooled and randomly split into training (90%) and validation (10%) sets. Models were subsequently developed individually on each of the 22 train sets, selected on the validation sets before predictions were obtained on the untouched test sets. The trained ensemble model was subsequently successfully validated in four independent external validation cohorts by infering predictions and replicating the downstream analysis.
Randomization	Allocation of participants to the respective training and validation sets was random. Clinical predictors were selected a priori, based on previous literature.
Blinding	This study did not require blinding as no subjective evaluation by an observer was involved.

Reporting for specific materials, systems and methods

We require information from authors about some types of materials, experimental systems and methods used in many studies. Here, indicate whether each material, system or method listed is relevant to your study. If you are not sure if a list item applies to your research, read the appropriate section before selecting a response.

Materials & experimental systems

n/a	Involved in the study	
☒	☐	Antibodies
☒	☐	Eukaryotic cell lines
☒	☐	Palaeontology and archaeology
☒	☐	Animals and other organisms
☒	☐	Human research participants
☒	☐	Clinical data
☒	☐	Dual use research of concern

Methods

n/a	Involved in the study	
☒	☐	ChIP-seq
☒	☐	Flow cytometry
☒	☐	MRI-based neuroimaging
